# Molecular pathogenesis of disease progression in *MLL*-rearranged AML

**DOI:** 10.1038/s41375-018-0253-3

**Published:** 2018-09-12

**Authors:** Shinichi Kotani, Akinori Yoda, Ayana Kon, Keisuke Kataoka, Yotaro Ochi, Yusuke Shiozawa, Cassandra Hirsch, June Takeda, Hiroo Ueno, Tetsuichi Yoshizato, Kenichi Yoshida, Masahiro M. Nakagawa, Yasuhito Nannya, Nobuyuki Kakiuchi, Takuji Yamauchi, Kosuke Aoki, Yuichi Shiraishi, Satoru Miyano, Takahiro Maeda, Jaroslaw P. Maciejewski, Akifumi Takaori-Kondo, Seishi Ogawa, Hideki Makishima

**Affiliations:** 1 Department of Pathology and Tumor Biology, Kyoto University, Kyoto, Japan; 2 Department of Hematology and Oncology, Kyoto University, Kyoto, Japan; 3 Department of Medical Oncology, Dana-Farber Cancer Institute, Boston, MA USA; 4 Department of Translational Hematology and Oncology Research, Cleveland Clinic, Taussig Cancer Institute, Cleveland, OH USA; 5 Division of Hematology, Department of Medicine, Brigham and Women’s Hospital, Harvard Medical School, Boston, MA USA; 6 Department of Medicine and Biosystemic Science, Kyushu University Graduate School of Medical Sciences, Fukuoka, Japan; 7 Laboratory of DNA Information Analysis, Human Genome Center, Institute of Medical Science, The University of Tokyo, Tokyo, Japan; 8 Center for Cellular and Molecular Medicine, Kyushu University Hospital, Fukuoka, Japan

**Keywords:** Cancer genetics, Acute myeloid leukaemia

## Abstract

Leukemic relapse is frequently accompanied by progressively aggressive clinical course. To understand the molecular mechanism of leukemic relapse, *MLL/AF9*-transformed mouse leukemia cells were serially transplanted in C57BL/6 mice (*N* = 96) by mimicking repeated recurrences, where mutations were monitored by exome sequencing (*N* = 42). The onset of leukemia was progressively promoted with advanced transplants, during which increasing numbers of somatic mutations were acquired (*P* < 0.005). Among these, mutations in *Ptpn11* (p.G60R) and *Braf* (p.V637E) corresponded to those identified in human *MLL*-AML, while recurrent mutations affecting *Msn* (p.R295C) were observed only in mouse but not in human *MLL*-AML. Another mutated gene of interest was *Gnb2* which was reported to be recurrently mutated in various hematological neoplasms. *Gnb2* mutations (p.G77R) were significantly increased in clone size (*P* = 0.007) and associated with earlier leukemia onset (*P* = 0.011). *GNB2* transcripts were significantly upregulated in human *MLL*-AML compared to *MLL*-negative AML (*P* < 0.05), which was supported by significantly increased *Gnb2* transcript induced by *MLL*/*AF9* overexpression (*P* < 0.001). In in vivo model, both mutation and overexpression of *GNB2* caused leukemogenesis, and downregulation of *GNB2* expression reduced proliferative potential and survival benefit, suggesting a driver role of *GNB2*. In conclusion, alterations of driver genes over time may play an important role in the progression of *MLL*-AML.

## Introduction

Despite advanced therapeutics, many leukemia patients become refractory to additional therapy, accounting for a major cause of leukemic deaths. Among major human acute myeloid leukemias (AMLs), *MLL*-rearranged AML (*MLL*-AML) is characteristic of poor prognosis due to refractoriness to chemotherapy and shorter period to relapse [1–3]. Multiple detailed and comprehensive studies in mouse models and human samples of *MLL*-AML have revealed an aberrant self-renewal of hematopoietic progenitors by constitutively activating self-renewal-related genes [4–7]. For maintenance of self-renewal potential in leukemia stem cells, chromatin modifications, including histone H3 lysine 79 (H3K79) and H3K4 methylation, play a critical role in *MLL*-AML [8–11]. MLL fusion proteins also activate transcription initiation by loading the TATA-binding protein (TBP) [12, 13]. According to these previous studies, epigenetic mechanisms downstream of MLL-fusion proteins are clearly involved in leukemogenesis in *MLL*-AML.

Recent advancements of sequencing technology and development of analytical tools allowed us to identify a whole spectrum of genetic events in various hematological neoplasms, as well as solid cancers. Multiple groups reported somatic mutations in human *MLL*-AML [14–17]. Among them, mutations involved in RAS pathway and receptor tyrosine kinase (RTK) genes were most frequently detected, suggesting that such signal transduction cascades might be activated in this leukemia. Even though less prevalent, mutations in the genes involved in DNA methylation and chromatin modification were also reported, indicating possible epigenetic synergy between *MLL* fusion genes and somatic mutations. However, it remains inconclusive whether these genetic events are associated with progression of the disease and short time to relapse. To clarify the molecular pathogenesis of progression in *MLL*-AML, we analyzed accumulation of genetic abnormalities in a mouse model of leukemia with human *MLL*/*AF9* fusion gene. Then, we validated the significance of driver mutations using both in-house and publicly available human mutation and gene-expression datasets in human samples. In addition, the further analysis of clonal architecture and prognostic significance of the driver mutations allowed us to identify a novel candidate gene, *Gnb2*. Finally we confirmed its functional relevance by in vitro and in vivo experiments of mouse *MLL*/*AF9* leukemia, a cytokine-dependent Ba/F3, and human hematopoietic cell lines.

## Materials and Methods

### Mouse model of *MLL*/*AF9* leukemia

**Fig. 1:**
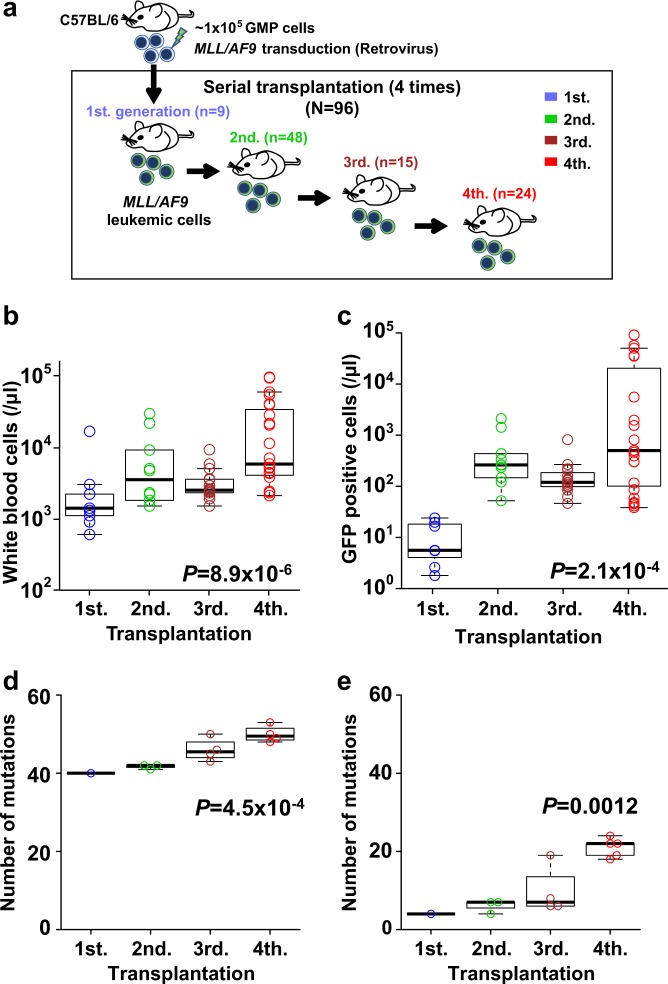
Serial transplantation of mouse *MLL*/*AF9*-acute myeloid leukemia (AML) (*N* = 96). **a** Serial transplantation of *MLL*/*AF9*-transduced granulocyte-monocyte progenitor (GMP) cells in C57BL/6 mice. **b** Total white blood cell (WBC) counts and **c** GFP-positive WBC counts on day 14. Numbers of mutations identified in strain A1 (**d**) and B1 (**e**)

We exploited a mouse model of recurrent leukemia generated by mouse progenitors transduced with human *MLL*/*AF9* as previously reported [7, 18]. Briefly, we isolated 1 × 10^5^ granulocyte macrophage progenitor (GMP) cells from C57BL/6 mice and transduced an *MLL*/*AF9* construct in them by a retrovirus system using a pGCDNsam-IRES-GFP vector. The GMP cells with *MLL*/*AF9* were transplanted into nine irradiated syngeneic mice (first generation), from which bone marrow mononuclear cells (BMMNCs) including GFP-positive mouse leukemic cells were collected. Then, 1 × 10^6^ BMMNCs per mouse were serially transplanted into 48 (second generation), 15 (third), and 24 (fourth) mice (Fig. 1a and Supplementary Figure 1). To assess disease status, we tested peripheral blood on day 14 for complete blood counts, white blood cell (WBC) phenotype (T-cell (CD4 and CD8α), B-cell (CD45R (B220)), and myeloid cell (CD11b and/or Gr-1)), and GFP positivity by flow cytometry (BD LSRFortessa X-20). In total, 96 mice were evaluated for survival periods and 57 for complete blood counts. All in vivo experiments in animals were approved by the Kyoto University Animal Care and Use Committee. To further confirm a pathogenic effect of *MLL*/*AF9* in mouse leukemic cells, we transduced this fusion gene in Ba/F3 by the same retrovirus system as above.

### Whole exome sequencing and confirmatory deep sequencing

We analyzed BMMNCs obtained from the *MLL*/*AF9* mouse model by whole exome sequencing (WES) (SureSelect XT Mouse All Exon V2). In total, we sequenced 42 mice in 8 primary AMLs (strains) from 5 donors as shown in Supplementary Figure 1. In three out of five donor mice, two strains were analyzed per mouse to compare mutation profiles between different strains from the same donors. We used DNA of mouse tails as germline control. Sequencing reads were mapped on GRCm38/mm10 by Burrows-Wheeler Aligner (BWA) [19] and candidate variants were called by Genomon pipeline [20]. Source BAM files of WES analysis were deposited under accession number PRJEB25881 in the European Nucleotide Archive. Then, we validated somatic variants by deep sequencing or Sanger sequencing of PCR amplicons as previously described [21]. To confirm relevance of somatic mutations, we translated the mutations in the mouse reference genome (GRCm38/mm10) into the corresponding protein substitutions in the human genome (GRCh38/hg38), in which driver mutations were already reported in human hematological malignancies. We also analyzed human *MLL*-AML samples (*N* = 53) by targeted sequencing as previously described [21], integrated the results with those from previously published datasets [14, 15, 22], and then compared mutations in human *MLL*-AML (*N* = 168) with those in mouse *MLL*-leukemia cells. Informed consent for sample collection was obtained according to a protocol approved by each Institutional Review Board in accordance with the Declaration of Helsinki.

### Gene-expression analysis

mRNA transcription levels of *GNB2* (NM_005273; 340 amino acids (AAs)), *Gnb1*, (NM_001160017; 340 AAs), and *Gnb2* (NM_010312; 340 AAs) were measured by SYBR green real-time RT-PCR using a primer set indicated in Supplementary Table 1 (Roche Molecular Systems, Inc.). Expression levels of 18S rRNA were used as internal control. RNA sequencing and expression array results of *GNB1* and *GNB2* in human AMLs and controls were downloaded from publicly available database (BioGPS; http://biogps.org) and previously published datasets [14].

### Transduction of wild type and mutant *GNB2*

Ba/F3 was stably transduced with Flag-wild-type and mutated *GNB2* (p.G77R) (pMIG vector) as described previously [23]. Protein levels were evaluated by immunoblot. Cells were lysed, subjected to SDS-PAGE, and transferred to PVDF membrane (Millipore). Each blot was incubated with anti-Flag antibody (#1E6, Wako Pure Chemicals) or anti-Actin antibody (#I-19, Santa Cruz Biotechnology), and its signal was visualized with Immobilon Western Chemiluminescent HRP Substrate (Millipore). Phosphorylation profile in the downstream signaling pathway of *GNB2* was assessed by immunoblot with anti-total AKT (#9272) and anti-pAKT (#9271) antibodies purchased from Cell Signaling. To investigate an effect of *GNB2* on leukemogenesis, Ba/F3 cells transduced with wild-type *GNB2*, *GNB2* mutant, and mock as above, were transplanted in immunodeficient mice. To assess *GNB2*-associated leukemic involvement, 2 × 10^6^ cells per mouse were intravenously transplanted into NOD/SCID/γc null (NOG) mice (*N* = 21 in total) after 2.5 Gy irradiation. GFP positivity was measured in bone marrow cells. To further study *GNB2*-associated tumorigenesis, 1 × 10^7^ cells per experiment were subcutaneously injected into nude mice (*N* = 30 in total) and the tumor size was measured every seven days.

### Suppression of *GNB2* by shRNA and depletion of *Gnb2* by CRISPR-Cas9

*MLL*-AML cell lines (NOMO1, MOLM13, and THP1) were infected with pLKO.1 lentivirus containing shRNAs targeting *GNB2* or a negative control scramble shRNA. shRNA sequences were designed according to the algorithm previously reported [24]. Targeted sequences are shown in Supplementary Table 2. Knockdown effects were measured by real-time RT-PCR as described above. To validate oncogenic potential of *GNB2* and *GNB1*, genome-wide CRISPR-Cas9 screens using Cas9-expressing mouse *MLL*/*AF9-*AML cells were performed as previously described [25].

### Statistics

Statistical analysis of means between two populations was performed using two-tailed, unpaired and/or paired Student’s *t*-test or nonparametric Mann–Whitney *U* test/Wilcoxon signed-rank test. For multiple testing, the false discovery rate (*q*-value) was calculated using the Benjamini–Hochberg procedure. Survival curves were assessed by Log-rank test. Correlation between the rank values of two variables was tested by Spearman rank correlation coefficient. Trends of categorical variables were assessed by the Jonckheere–Terpstra test. Statistical analyses and generation of the fishplot (https://github.com/chrisamiller/fishplot/blob/master/tests/test.R) were performed with R (https://www.r-project.org) and/or JMP9 software (SAS). A *P*-value and *q*-value of less than 0.05 was considered significant. Error bars in all graphs represent the standard error of the mean.

## Results

### Leukemic transformation and onset in serial transplantation

To confirm the disease progression of *MLL*/*AF9* mouse leukemia, we evaluated 57 serially transplanted mice for peripheral blood counts and WBC phenotypes on day 14, BMMNC phenotypes, and time to leukemic evolution. By trend test (Jonckheere–Terpstra test) for the later transplant, numbers of WBC and GFP-positive WBC increased significantly (*P* = 8.9 × 10^−6^ and *P* = 2.1 × 10^−4^) (Fig. 1b, c), while hemoglobin levels and platelet counts significantly decreased (*P* = 3.1 × 10^−4^ and *P* = 0.0034, respectively) (Supplementary Figure 2a, b). Flow cytometry analysis of the WBC revealed that myeloid cells were significantly more dominant than T-cells and B-cells (*P* < 1.0 × 10^−15^ and *P* < 1.0 × 10^−15^) (Supplementary Figure 3). BMMNCs showed that GMP and megakaryocyte-erythroid progenitor (MEP) populations were positive for GFP (Supplementary Figure 4). Median periods to leukemia onset were 57 and 16 days in the first and the fourth transplants, respectively. Statistically, the onset of leukemia became progressively earlier in the later transplant (*P* = 3.6 × 10^−10^) (Supplementary Figure 2c). These results indicate that serial transplantation of *MLL*/*AF9*-transduced mouse leukemic cells resulted in shorter time to repopulate the bone marrow, which showed an aggressive phenotype similar to that of relapsed human *MLL*-AML.

### Accumulation of somatic mutations in mouse *MLL*-AML

**Fig. 2:**
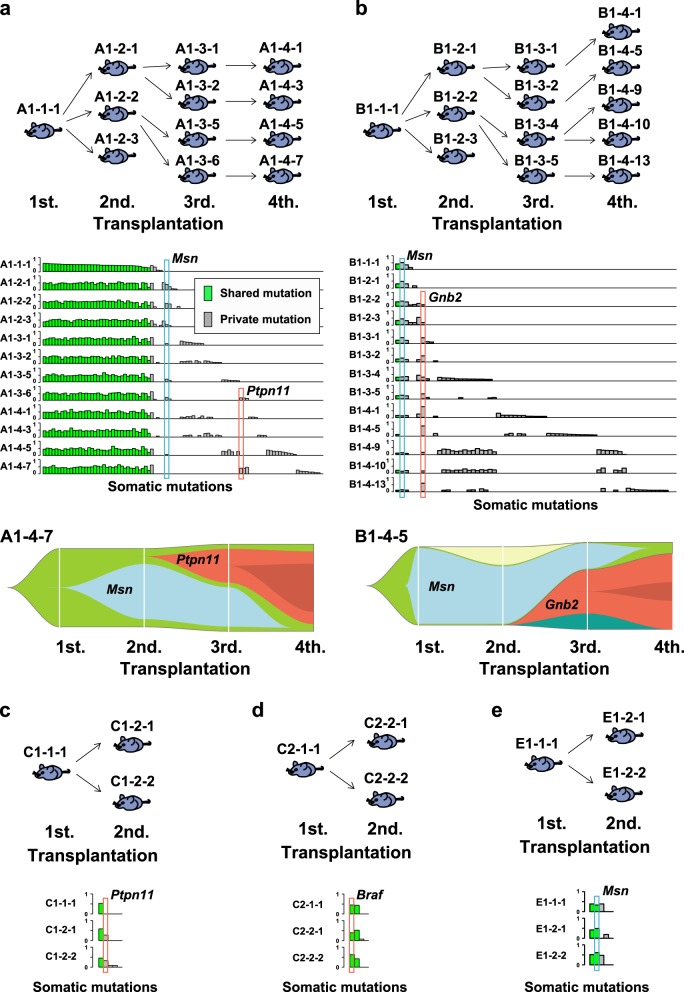
Whole exome sequencing (WES) of mouse *MLL*/*AF9*-AML. **a**, **b** Upper panels showed 12 and 13 mice analyzed by WES in strain A1 and B1, respectively. Middle panels demonstrated landscape of somatic mutations in each strain. Green and gray indicated shared and private mutations, respectively. Driver mutations were represented in bold italic font. In lower panels, fishplots displayed clonal architecture in A1-4-7 and B1-4-5 mice according to the results of mutational landscape in their own and their ancestry transplant generations. **c**–**e** Upper panels showed each 3 mice per strain from strain C1, C2, and E1, respectively. Lower panels demonstrated landscape of somatic mutations in each strain

To identify somatic mutations acquired during serial transplantation, we analyzed eight strains originated from five donor mice with transduced *MLL*/*AF9* (Supplementary Figure 1), where two strains per donor mice were analyzed for donor A, C, and D. In total, leukemic cells obtained from 42 recipient mice in the first (*N* = 8), second (*N* = 17), third (*N* = 8), and fourth (*N* = 9) transplants were analyzed by whole exome sequencing. The mean sequence depth for all tumor and normal samples was 126.3 × (Supplementary Figure 5). By BWA alignment and Genomon pipeline as previously described [21, 26, 27], 1084 candidate variants (the cumulative total number) were extracted, out of which we randomly selected 33 variants and 97% (32/33) of them were confirmed as somatic by deep sequencing. We applied this default filter to call the rest of mutations. As a result, 968 missense, 7 frameshift (6 deletion and 1 insertion), 67 nonsense, and 41 splice site mutations were identified in 42 mice (25.8 mutations per mouse) (Supplementary Table 3). Out of these, in the experiments of two mouse strains (strain A1 and B1), the cells obtained from different donor mice were serially transplanted four times (Supplementary Figure 1). During the serial transplantation, somatic mutations were significantly accumulated, demonstrating significant trends of increasing number of mutations in both strains (A1; *P* = 4.5 × 10^−4^ and B1; *P* = 0.0012, respectively) (Fig. 1d, e). To assess variation of mutations in each strain, we removed overlaps and calculated actual number of mutations; 93 and 65 mutations were identified in these strains, respectively (Fig. 2a, b), and no mutations except for one (*Msn* mutation) were shared between the strains (Supplementary Table 3). To further investigate the cause of the difference in acquired mutations, we compared mutational spectra between each set of two strains from the same donors (A1 *vs.* A2, C1 *vs.* C2, and D1 *vs.* D2). No shared mutation was identified in any set from the same donor (Supplementary Table 3), suggesting that the variation of the mutations were not derived from different donor cells but from different primary AML cells after the first transplantation.

### Candidate pathogenic mutations in mouse *MLL*/*AF9*-AML

**Fig. 3:**
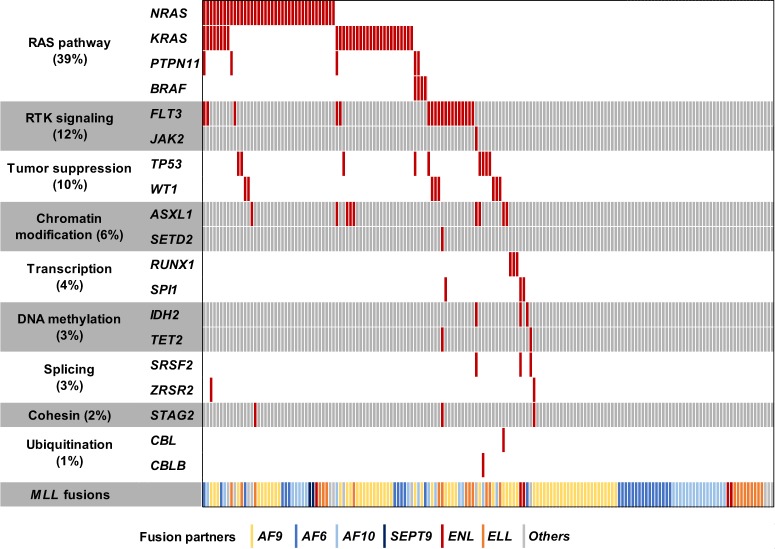
Mutational spectrum in human *MLL*-AML (*N* = 168). A frequency of mutations (%) was shown in each affected leukemogenic pathway

**Fig. 4:**
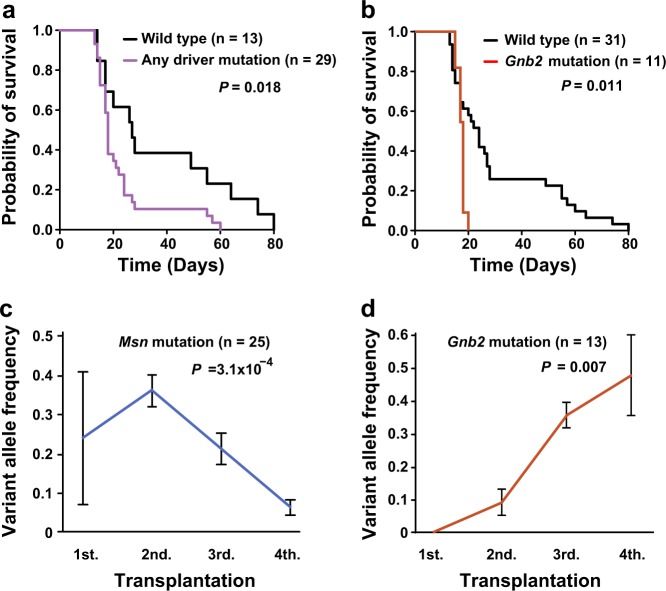
Impact of driver mutations on leukemia onset and clonal expansion in *MLL*/*AF9*-AML. **a**, **b** Comparison of leukemia onset time between cases with and without driver mutations (**a**) or *Gnb2* mutations (**b**). **c**, **d** Assessment of trend in variant allele frequencies of *Msn* (**c**) or *Gnb2* (**d**) mutations

Among whole spectrum of somatic mutations in multiple generations of mouse *MLL*/*AF9*-AML transplantation from eight different strains, we extracted candidate driver mutations according to two criteria; (1) mutations recurrently identified in the multiple independent strains or (2) mutations reported in human hematological neoplasms. Mutations of *Ptpn11* (p.G60R) were found in two independent strains (A1 and C1) (Fig. 2a, c) and also in human *MLL*-AML samples (Fig. 3). *Msn* mutations (p.R295C) were also recurrent in sequenced mouse *MLL*/*AF9*-AML from 3 strains (Fig. 2) and an identical mutation was reported in mouse *CALM*/*AF10* leukemia models [28]. In addition, *Braf* mutations (p.V637E) detected in all three mice of strain C2 (Fig. 2d) corresponded to those identified in human *MLL*-AML (*BRAF* p.V600E) (Fig. 3). Another mutated gene of interest was *Gnb2* in Strain B1 mice (Fig. 2b) which was reported to be recurrently mutated in various human hematological neoplasms [23]. Of note is that these mutations were located at the well-conserved residues among species. If all of them were combined, these candidate driver mutations were significantly associated with earlier leukemia onset in our mouse *MLL*-AML model (*P* = 0.018) (Fig. 4a). Among them, while *Msn*, *Ptpn11*, or *Braf* mutation had no significant impact (Supplementary Figure 6a-c), *Gnb2* mutation was a significant poor prognostic factor compared to wild type (*P* = 0.011) (Fig. 4b). In contrast, the number of whole mutations wasn’t significantly associated with time of leukemia onset (Supplementary Figure 2d). These results suggest that acquisition of mutations in driver genes especially *Gnb2* plays a more important role in shortening of time to leukemia onset in mouse *MLL*/*AF9*-AML than mutational accumulation itself.

### Clonal architecture in mouse *MLL*/*AF9*-AML

To further investigate clonal dynamics of the driver mutations, landscape of the mutations was demonstrated in every strain, where all the mutations were divided into either shared or private ones (Fig. 2). In strain A1 where 12 mice from 4 generations were analyzed, 36 were shared mutations and 57 were private ones (Fig. 2a). In strain B1, 1 mutation was shared and 64 were private (Fig. 2b). If all the sequenced strains were included (Fig. 2 and Supplementary Figure 7), shared and private mutations were 15.7 and 19 per strain on average, respectively. Based on these results of mutational landscape and variant allele frequency, clonal dynamics was displayed by the fishplots, showing that complex clonal architecture consisted of multiple decreased and newly emerged clones. While clones with *Msn* mutations were dominant in the first and second generations, new clones with *Ptpn11* and *Gnb2* mutations emerged in the second or third generations, and swept the initial *Msn* mutant clones out in the fourth generations (Fig. 2a, b). Integrating all the mutations identified in strains A1 and B1, we found that variant allele frequency significantly increased in size during serial transplantation (*P* = 1.7 × 10^−14^) (Supplementary Figure 6d). Next, we were able to conclude clonal dynamics of driver mutations. While the clone size of *Gnb2* mutations significantly elevated during serial transplantation (*P* = 0.007) (Fig. 4d), those of *Msn*, *Ptpn11*, or *Braf* mutations did not significantly increase (Fig. 4c and Supplementary Figure 6e, f). Though *GNB2* mutations were not identified in human *MLL-*AML (Fig. 3), *Gnb2* mutation had a significant impact on poor prognosis in mouse *MLL*/*AF9*-AML, increased in clone size during the serial transplantation, and existed in other human hematological neoplasms [23]. Therefore, we assumed that *GNB2* might be upregulated by previously unknown mechanisms as a new oncogene in human *MLL-*AML.

### *GNB2* expression in human *MLL*-AML

**Fig. 5:**
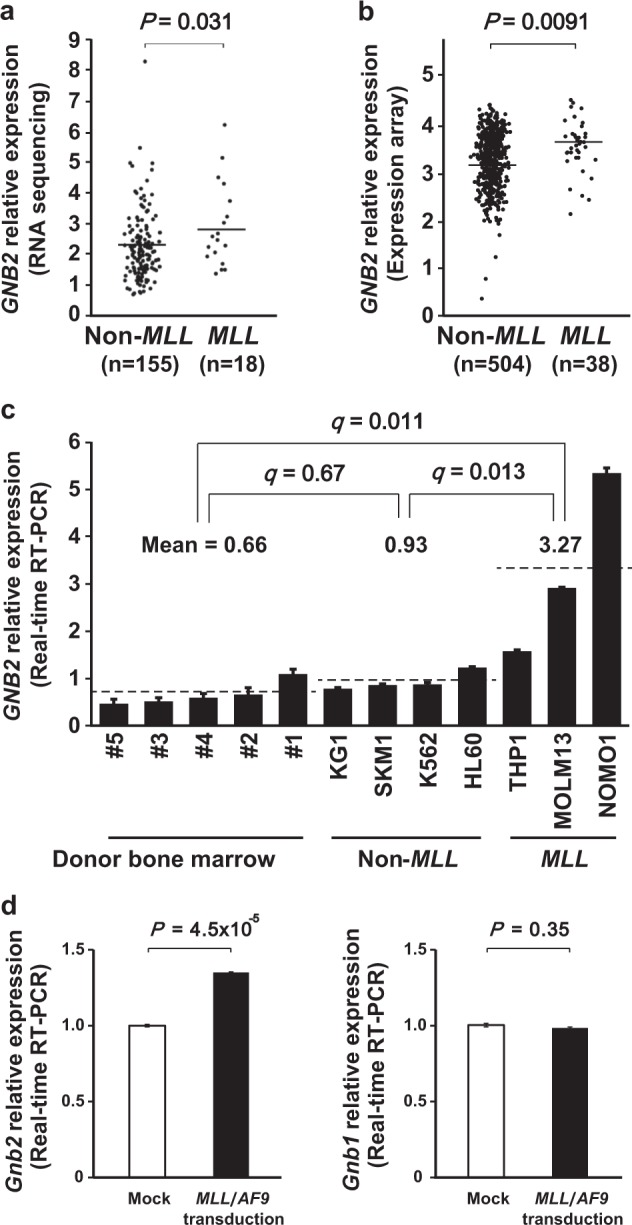
*GNB2* expression in human *MLL*-AML. **a**, **b** *GNB2* relative expression in human AML with and without *MLL*-fusion gene (**a** RNA sequencing and **b** expression array). **c** *GNB2* relative expression in bone marrow mononuclear cells (BMMNCs) from healthy donors (*N* = 5), and AML cell lines with (*N* = 3) and without *MLL*-fusion gene (*N* = 4) (real-time RT-PCR). Each experiment was performed in triplicate. The Benjamini–Hochberg procedure was applied to the correction of multiple testing. **d** *Gnb1* and *Gnb2* expression levels were assessed in comparison between empty- and *MLL*/*AF9*-transduced Ba/F3 cells. Each experiment was performed in triplicate

To further investigate pathological significance of *GNB2*, we analyzed the gene expression of *GNB2* in human *MLL*-AML. In normal tissues, *GNB2* is expressed in whole blood and bone marrow cells (Supplementary Figure 8). When compared between human *MLL*-rearrangement ( + ) (*N* = 18) and *MLL*-rearrangement (−) (*N* = 155) AML cases, mRNA levels of *GNB2* extracted from RNA sequencing dataset were significantly upregulated in human *MLL*-rearrangement ( + ) cases (*P* *=* 0.031) (Fig. 5a). In the same cohort, we also compared *GNB1* expression but identified no significant difference in contrast to the result of *MLL/AF9*-dependent upregulation of *GNB2* (Supplementary Figure 9). To confirm this finding, we analyzed *GNB2* expression by microarray in a larger cohort of AML. As a result, we again showed significantly higher expression of *GNB2* in human *MLL-*AML (*N* = 38) than in non-*MLL* AML (*N* = 504) (*P* *=* 0.0091) (Fig. 5b). Finally, we assessed *GNB2* expression by real-time RT-PCR in healthy donors (*N* = 5), human non-*MLL* AML cell lines (*N* = 4), and *MLL*-AML cell lines (*N* = 3). *GNB2* expression was not significantly different between healthy donor BM cells and non-*MLL* AML cell lines (*q* *=* 0.67). However, *MLL*/*AF9*-positive cell lines, THP1, MOLM13, and NOMO1 significantly highly expressed this gene compared to healthy donor BM cells (*q* *=* 0.011) and non-*MLL* AML cell lines (*q* = 0.013) (Fig. 5c). To further validate these findings, we assessed *Gnb1* and *Gnb2* expression levels in comparison between empty- and *MLL*/*AF9*-transduced Ba/F3 cells and identified *MLL*/*AF9*-dependent upregulation of *Gnb2* (*P* = 4.5 × 10^−5^) (Fig. 5d). These findings suggest the potential status of *GNB2* as a candidate oncogene in human *MLL*-AML as well as in mouse model, and therefore we further performed confirmatory functional experiments.

### Oncogenic potential of mutant and wild-type *GNB2*

**Fig. 6:**
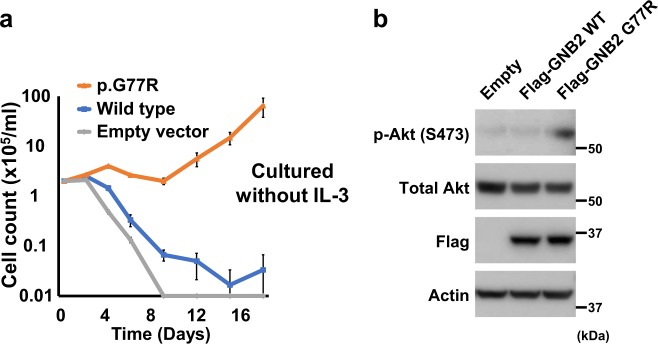
Cytokine independency due to *GNB2*. **a** Growth curves of Ba/F3 cells cultured without IL-3 after transduction of wild type or mutated (p.G77R) *GNB2*. Each experiment was performed in triplicate. **b** Protein levels of total Akt and phosphorylated Akt (p-Akt) were evaluated by immunoblot

**Fig. 7:**
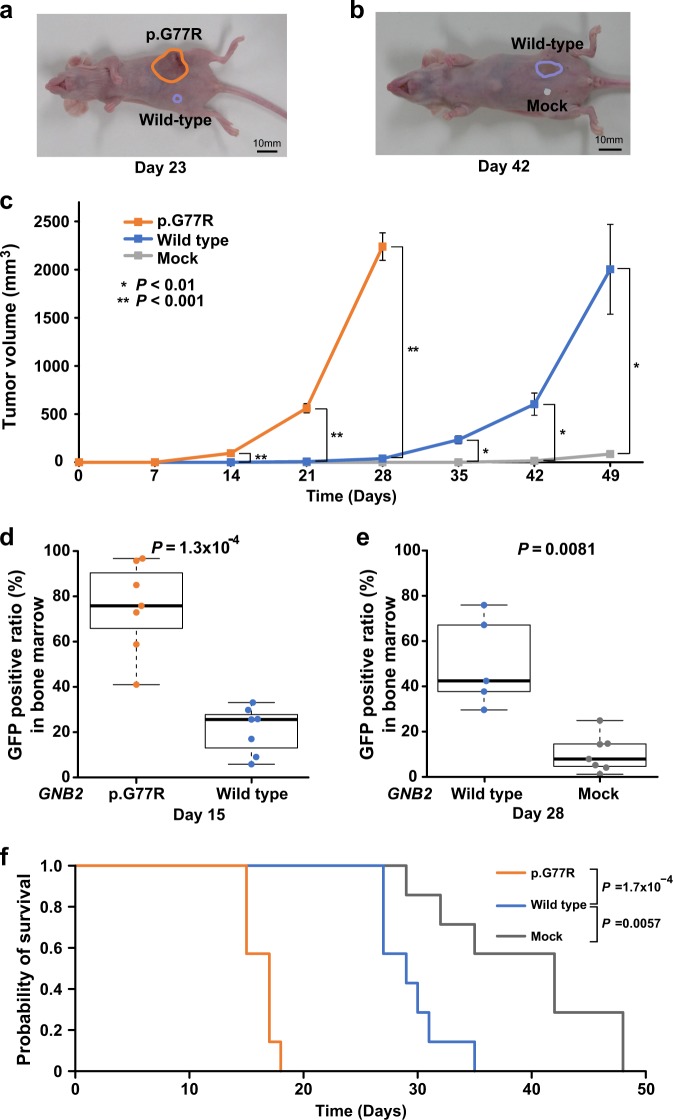
Oncogenic potential of mutant and wild-type *GNB2*. Ba/F3 cells transduced with wild-type *GNB2*, *GNB2* mutant, and mock were transplanted in immunodeficient mice. **a**, **b**, **c** To study *GNB2*-associated tumorigenesis, 1 × 10^7^ cells per experiment were subcutaneously injected into nude mice (*N* = 30 in total). The tumor size was measured (**a** on day 23 and **b** on day 42) and compared between 3 groups (**c**). **d**, **e**, **f** To assess *GNB2*-associated leukemic involvement, 2 × 10^6^ cells per mouse were intravenously transplanted into NOD/SCID/γc null (NOG) mice (*N* = 21 in total). GFP positivity was measured in bone marrow cells (**d** on day 15 and **e** on day 28). Survival time was compared between 3 groups (**f**)

We transduced wild type and mutant (p.G77R) *GNB2* in an interleukin-3 (IL-3) dependent leukemic cell line Ba/F3. Overexpression of mutant *GNB2* conferred cytokine-independent proliferation (Fig. 6a). To further investigate an oncogenic effect of the *GNB2* mutation on cytokine independency, we assessed activation of downstream signaling pathway in Ba/F3 cells transduced with the mutation. Phosphorylation of AKT was upregulated in mutant Ba/F3 (Fig. 6b), suggesting activation of PI3K/Akt/mTOR pathway. Overexpressed wild-type *GNB2* also resulted in maintaining proliferation even after IL-3 withdrawal, while mock cells cultured without IL-3 showed cell death (Fig. 6a). To further investigate tumor formation associated with *GNB2*, we performed in vivo experiments in which Ba/F3 cells with mock, wild-type *GNB2*, and mutant *GNB2* were transplanted subcutaneously to nude mice (Fig. 7a–c) and intravenously to NOG mice (Fig. 7d–f). Compared between wild type *vs.* mutant *GNB2* experiments in nude mice, tumor volumes were significantly larger in mutant than in wild type (*P* < 0.001 on day 14, 21, and 28) (Fig. 7a, c). Next compared between mock *vs.* wild-type *GNB2* groups, tumor volumes were significantly larger in wild-type experiments than in mock (*P* < 0.01 on day 35, 42, and 49) (Fig. 7b, c). We further assessed leukemogenesis due to *GNB2* alterations (both mutation and overexpression) in NOG mice. GFP-positive ratio in bone marrow cells was significantly higher in mutant experiments ( > 40%) than in wild-type ones on day 15 (*P* *=* 1.3 × 10^−4^) (Fig. 7d), and also significantly elevated in wild type ( > 30%) than in mock on day 28 (*P* *=* 0.0081) (Fig. 7e). Therefore, all NOG mice with mutant or wild-type *GNB2* cells were proved to be affected by leukemic evolution. When compared between mutant, wild type, and mock experiments, survival period was significantly shorter in mutants than in wild type (*P* = 1.7 × 10^−4^) and also in wild type than in mock (*P* = 0.0057) (Fig. 7f). These findings suggest that upregulation of wild-type *GNB2* had a significant leukemogenic effect, although it is less potent than that of mutant *GNB2*.

### Effect of decreased expression of wild-type *GNB2*

**Fig. 8:**
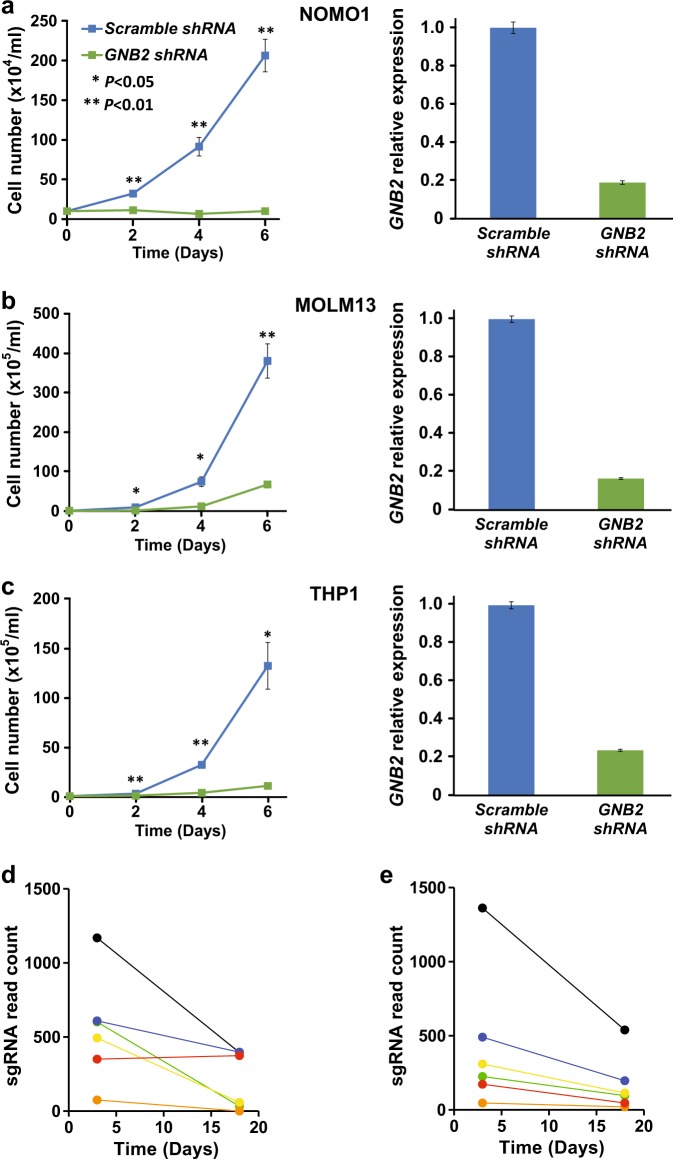
Impact of decreased *GNB2* expression or depleted *Gnb2* genome in *MLL*-AML. **a**, **b**, **c** Growth curves (cell numbers) and *GNB2* expression values of 3 human *MLL*-AML cell lines (**a** NOMO1, **b** MOLM13, and **c** THP1) treated with *GNB2* shRNA and control scramble shRNA. Each experiment was performed in triplicate. **d**, **e** By genome-wide CRISPR-Cas9 screens, read counts of six sgRNAs targeting per exon (exon 1 (**d**) and 2 (**e**)) in *Gnb2* were measured after a 16-day incubation period to confirm potential oncogenic function of *Gnb2* in *MLL*/*AF9-*AML

As mentioned, wild-type *GNB2* expression was elevated in human *MLL*-AML, which is consistent with the remarkable in vivo leukemogenesis due to overexpression of wild-type *GNB2*. To further confirm these findings, we reduced *GNB2* expression by shRNA and depleted *Gnb2* by CRISPR-Cas9 system. First, knocked down wild-type *GNB2* in *MLL*-AML cell lines; NOMO1, MOLM13, and THP1, which showed high expression of wild-type *GNB2* (Fig. 5c), resulted in significantly less proliferation compared to mock experiments (Fig. 8a–c). In agreement with these findings, *Gnb2* was found to be essential for mouse *MLL*/*AF9*-AML, revealed by genome-wide CRISPR-Cas9 screens [25]. sgRNAs targeting *Gnb2* were significantly depleted over a 16-day incubation period, confirming a potential oncogenic function of wild-type *Gnb2* in *MLL*-AML (*P* = 0.00366) (Fig. 8d, e). In contrast, sgRNAs targeting *Gnb1* showed no positive impact on cell survival in the CRISPR-Cas9 screen (Supplementary Figure 10), which confirmed the results of normal *GNB1* expression in human *MLL*-AML (Supplementary Figure 9).

## Discussion

Our results from whole exome sequencing of serially transplanted mouse *MLL*/*AF9*-AML cells and confirmatory genetic and functional analyses of human *MLL*-AML provided insights into the molecular mechanism of leukemic progression in *MLL*-AML. Accumulation of somatic mutations in addition to *MLL*-fusion gene was common in the sequencing experiments of our mouse model and clinical samples. As well as well-known driver mutations (*Ptpn11* and *Braf*), we found novel *Msn* and *Gnb2* mutations in our mouse model. Out of these candidate mutations, clone size of *Gnb2* mutation significantly elevated during serial transplantation and this mutation was significantly associated with earlier leukemia onset. These results suggest that *GNB2* is a candidate pathogenic gene in the human disease, which was convincingly supported by high expression of this gene in human *MLL-*AML. The pathologic role of high expression of *GNB2* was confirmed by its knockdown and knockout experiments in in vitro and in vivo disease models. Accordingly, additional mutational and transcriptome events co-occuring with *MLL*-fusion genes might play a significant role in the pathogenesis of progressive clinical course in *MLL-*AML.

As previously reported in myelodysplastic syndromes, accumulation of multiple mutations are seen at disease presentation and closely related to secondary AML evolution [21, 29, 30]. In parallel, leukemic cells of human core binding factor (CBF) leukemia frequently acquired additional mutations after initial hits of leukemogenic fusion genes [31]. CBF leukemia and *MLL*-AML showed similar types of genes mutated in secondary fashion, involving signal transduction (RAS pathway and RTK cascade), IDH family, and gene transcription, of which all are essential for progressive clinical phenotypes in AML. In this study of *MLL*/*AF9*-AML, we showed that somatic mutations remarkably accumulated during shortening leukemic onset and that driver mutations in the mouse sequencing reproduced human mutational profiles, including RAS pathway mutations. Of note, the presence of driver mutations were more associated with *MLL*/*AF9*-AML onset than the number of whole mutations. Analysis of complex clonal dynamics revealed distinct behaviors of clones with *Msn* mutations which were swept out by those with other driver mutations. Especially, clone size of mutations in *Gnb2*, which is involved in signal transduction, increased during the reduced clone size of *Msn* mutations and the shortened leukemic onset. These findings suggest that clonal dynamics of specific genetic events in *MLL*/*AF9* AML might be associated with relapse and aggressiveness of this disease.

In general, mutations and overexpression of oncogenes provide oncogenic potential due to increased function and quantity of the coding proteins, respectively. In AML, oncogenes sometimes display activating mutations and high expression simultaneously, both of which are supposed to have a leukemogenic effect on the affected hematopoietic stem cells and/or myeloid progenitor cells. In this study of *MLL*/*AF9*-AML, we showed that a novel activating mutation of *Gnb2* were clearly pathogenic in a mouse leukemia model and that high expression of *GNB2* may play a significant role in human *MLL*-AML. Hence, our study posits the oncogenic candidacy of *GNB2* in *MLL*-AML. *GNB2* mutations were also identified in several cancers, including diffuse large B-cell lymphoma, colorectal adenocarcinoma, and lung small cell carcinoma [32–34]. After exploring and elucidating the role of *GNB2*, our study finds it reasonable to suggest *GNB2* as an oncogene presenting its distinct phenotype of clinical progression in the synergistic manner with *MLL*-fusion gene.

As previously reported, expression of various oncogenes, which are responsible for leukemogenesis in *MLL*-AML, are regulated by *MLL*-fusion genes through histone modifications. For example, highly expressed Hox genes are associated with altered functions of histone methyltransferases and demethylases which directly or secondarily regulate methylation and acetylation status of histone H3K4, H3K36, and H3K79 [7, 9, 10, 35]. As well as such reported driver genes, *GNB2* was consistently expressed to serve as an oncogene in human *MLL-*AML, although no somatic mutations in this gene were identified. In fact, according to ChIP sequencing of multiple hematopoietic cell lines, promoter sites of *GNB2* were remarkably identified as binding regions of tri-methylated histone H3K4, which serves a major role of oncogenic potential in *MLL*-associated leukemia [10]. Therefore, expression of *GNB2*, whose activating mutation was acquired during mouse *MLL*/*AF9* leukemia, might be epigenetically regulated by MLL fusion protein.

*GNB2* encodes a β subunit of heterotrimeric G proteins, which consist of Gα, Gβ, and Gγ components that mediate signal transduction downstream of G protein-coupled receptors (GPCR). Our study and a recent paper demonstrated that GNB family proteins may also take part in non-GPCR signaling, for instance, all other types of receptor-mediated signaling, including various cytokine receptors and JAK/STAT pathways [23]. According to these concepts, RTKs and RAS pathway, which are most frequently mutated in *MLL*-AML, are in the downstream of the sequential signaling cascades activated by GNB2. To develop the therapeutic approach for *GNB2* activation, we could further focus our attention on RTK inhibitors and RAS pathway inhibitors targeting molecules up- and downstream of this gene, suggesting that *GNB2* could be a novel therapeutic target to already FDA-approved drugs.

In conclusion, this is the first comprehensive study of somatic mutations in *MLL*/*AF9* mouse model by whole exome sequencing. Overall, we postulate that *MLL*-fusion genes as initial drivers may cooperate subsequently with various somatic genetic events, resulting in aggressive clinical course due to leukemic progression.

## Electronic supplementary material

Supplemental Material
