# Supplementary material for: Molecular pathogenesis of disease progression in MLL-rearranged AML
Source: Leukemia. 2018 Sep 12;33(3):612–24. doi: 10.1038/s41375-018-0253-3 (PMC6462875; doi:10.1038/s41375-018-0253-3)
Supplement: Supplementary file 1 — Supplemental Material [file 41375_2018_253_MOESM1_ESM.pdf]

Supplementary Figure 1

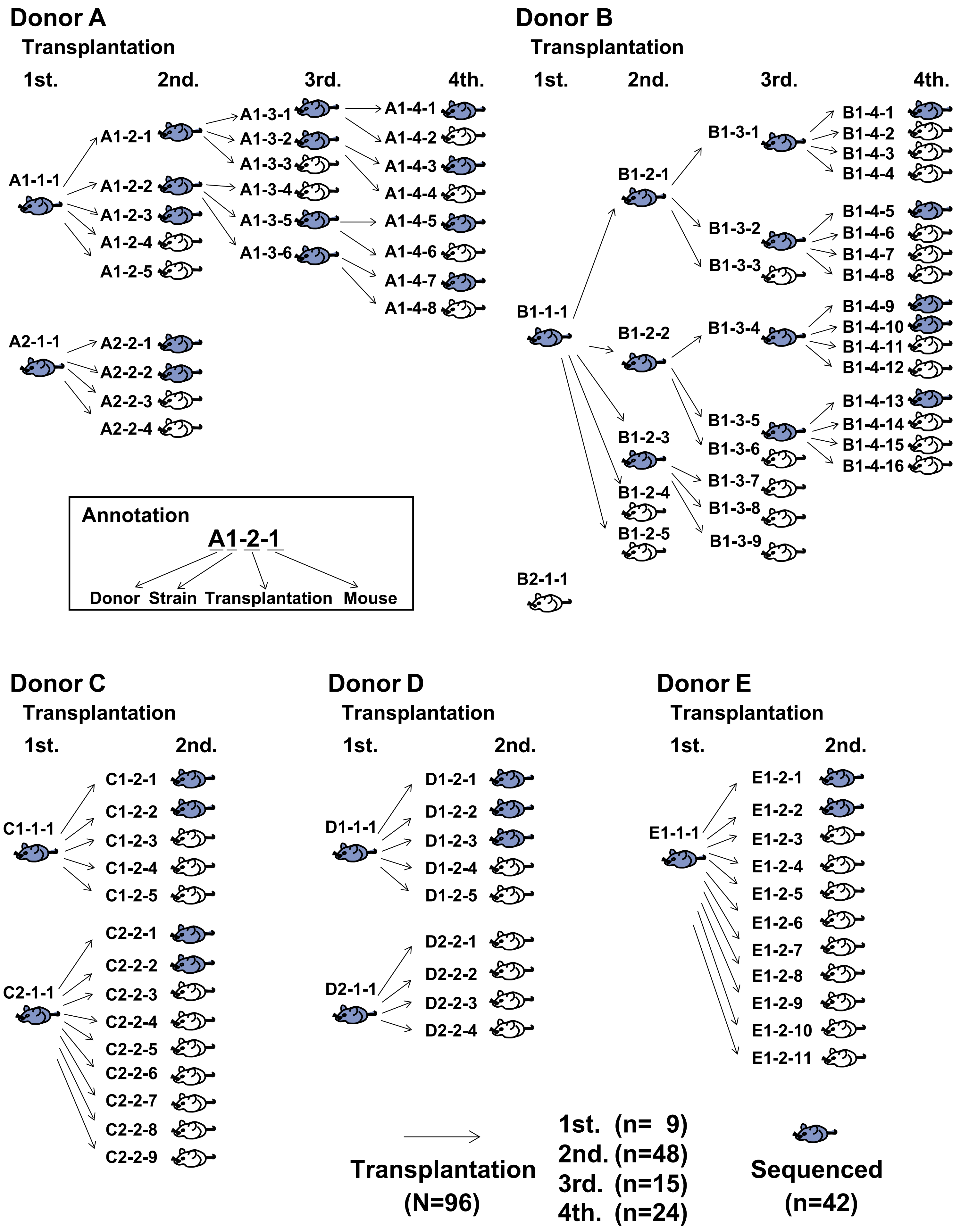

Supplementary Figure 1. Transplantation of mouse *MLL/AF9* acute myeloid leukemia (*MLL/AF9*-AML).
Serial transplantation of mouse *MLL/AF9*-AML was performed in C57BL/6 mice (N=96).
Blue mice were analyzed by whole exome sequencing but white ones were not.

Supplementary Figure 2

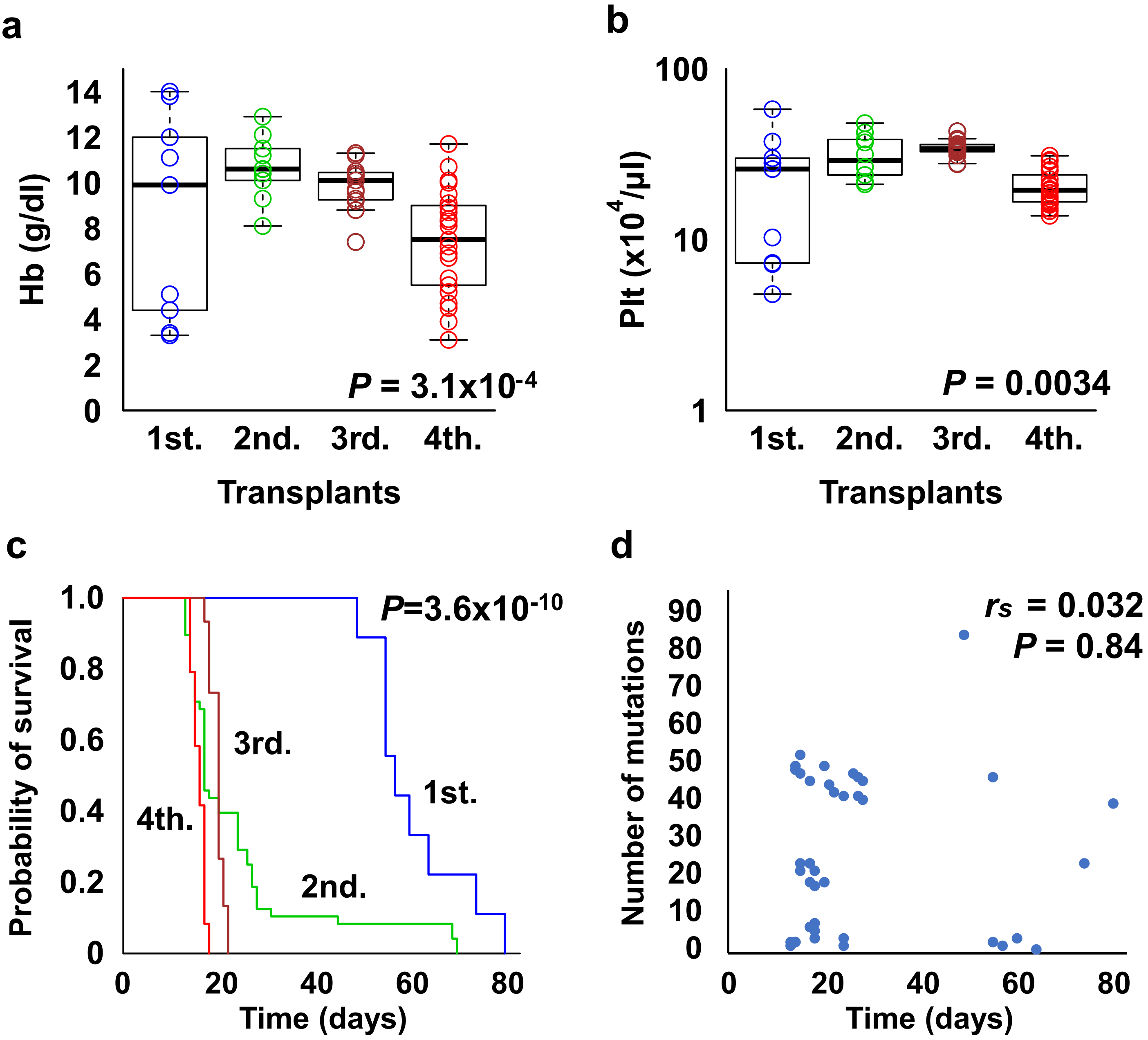

Supplementary Figure 2. Blood counts, relapse time, and number of mutations during serial transplantation of mouse *MLL/AF9* acute myeloid leukemia.
Hemoglobin levels (Hb) (**a**) and platelet counts (Plt) (**b**) were measured on day 14 after transplantation. Trends of changes in the blood counts (**a, b**) and time to leukemia onset after transplantation (**c**) were assessed by the Jonckheere-Terpstra test. The correlation between number of mutations and time of leukemia onset was assessed by Spearman's rank correlation coefficient (**d**).

Supplementary Figure 3

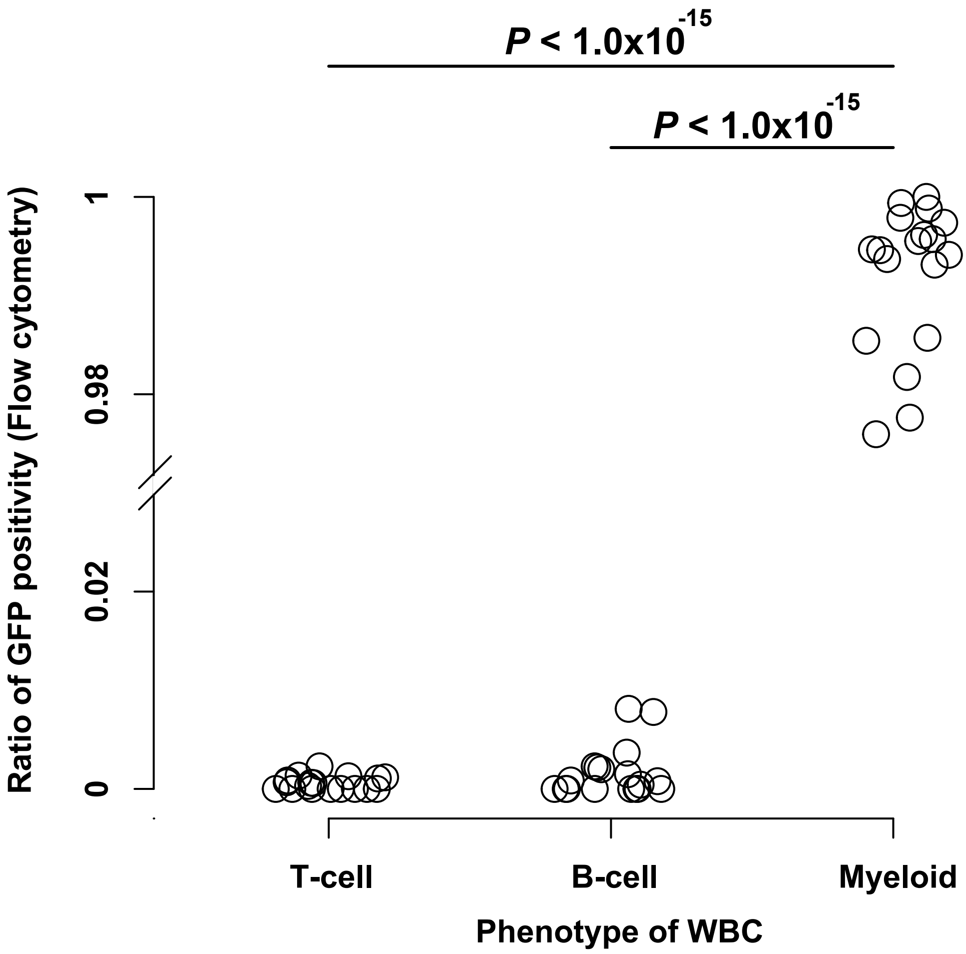

Supplementary Figure 3. Phenotype of WBC in peripheral blood in *MLL/AF9*-AML mouse model. In serial transplantation of mouse *MLL/AF9* acute myeloid leukemia, GFP positivity was examined by flowcytometry in T-cell, B-cell, and myeloid populations.

Supplementary Figure 4

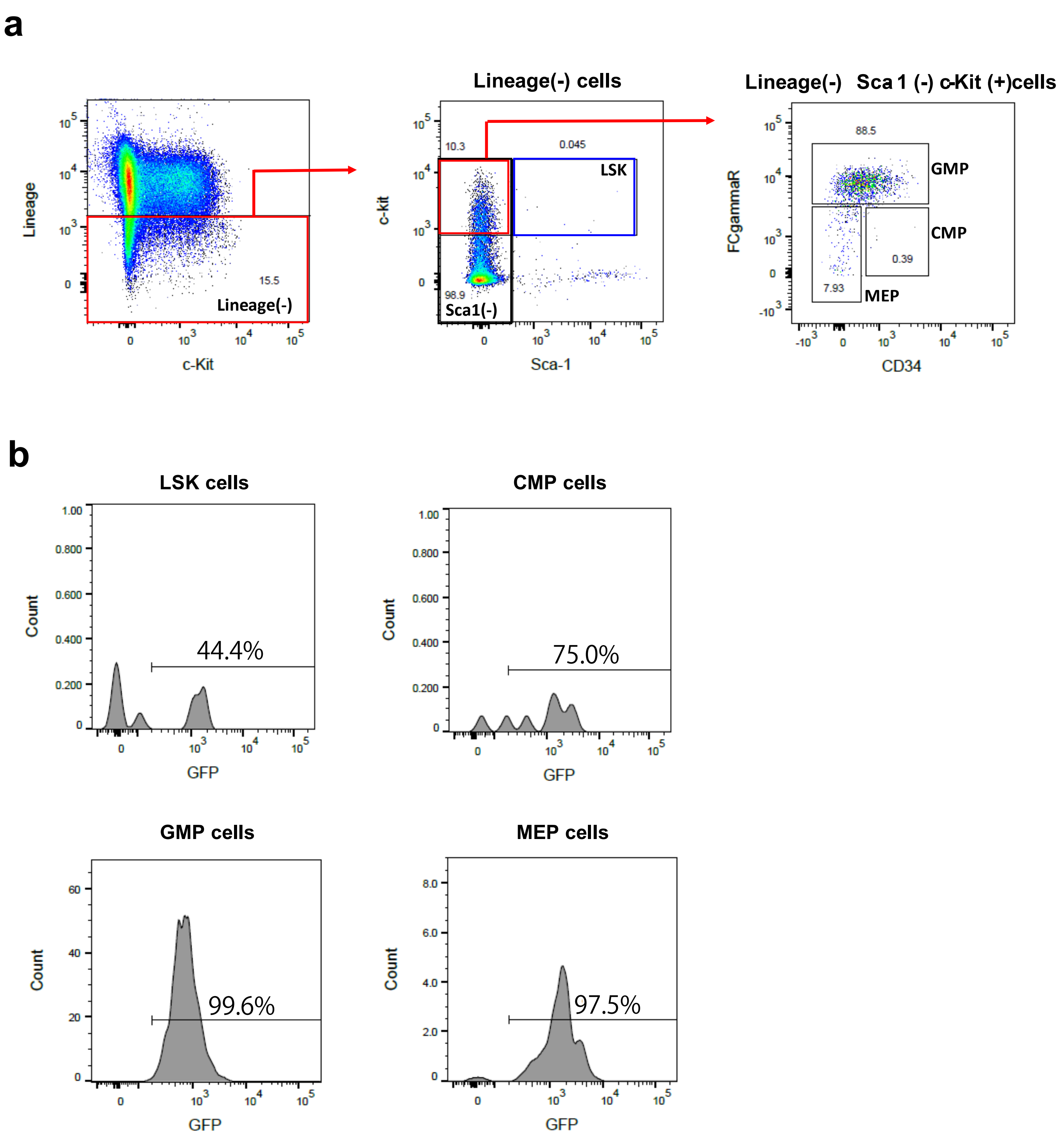

Supplementary Figure 4. Phenotype of mouse *MLL/AF9* acute myeloid leukemia.
Phenotype of mouse *MLL/AF9* acute myeloid leukemia cells was examined by flowcytometry using fluorescence-conjugated anti-lineage, Sca1, c-Kit, FcR gamma II/III, and CD34 antibodies (**a**). GFP positivity was measured in LSK, CMP, GMP, and MEP populations by flowcytometry (**b**). Representative results were shown in the second transplantation.

Supplementary Figure 5

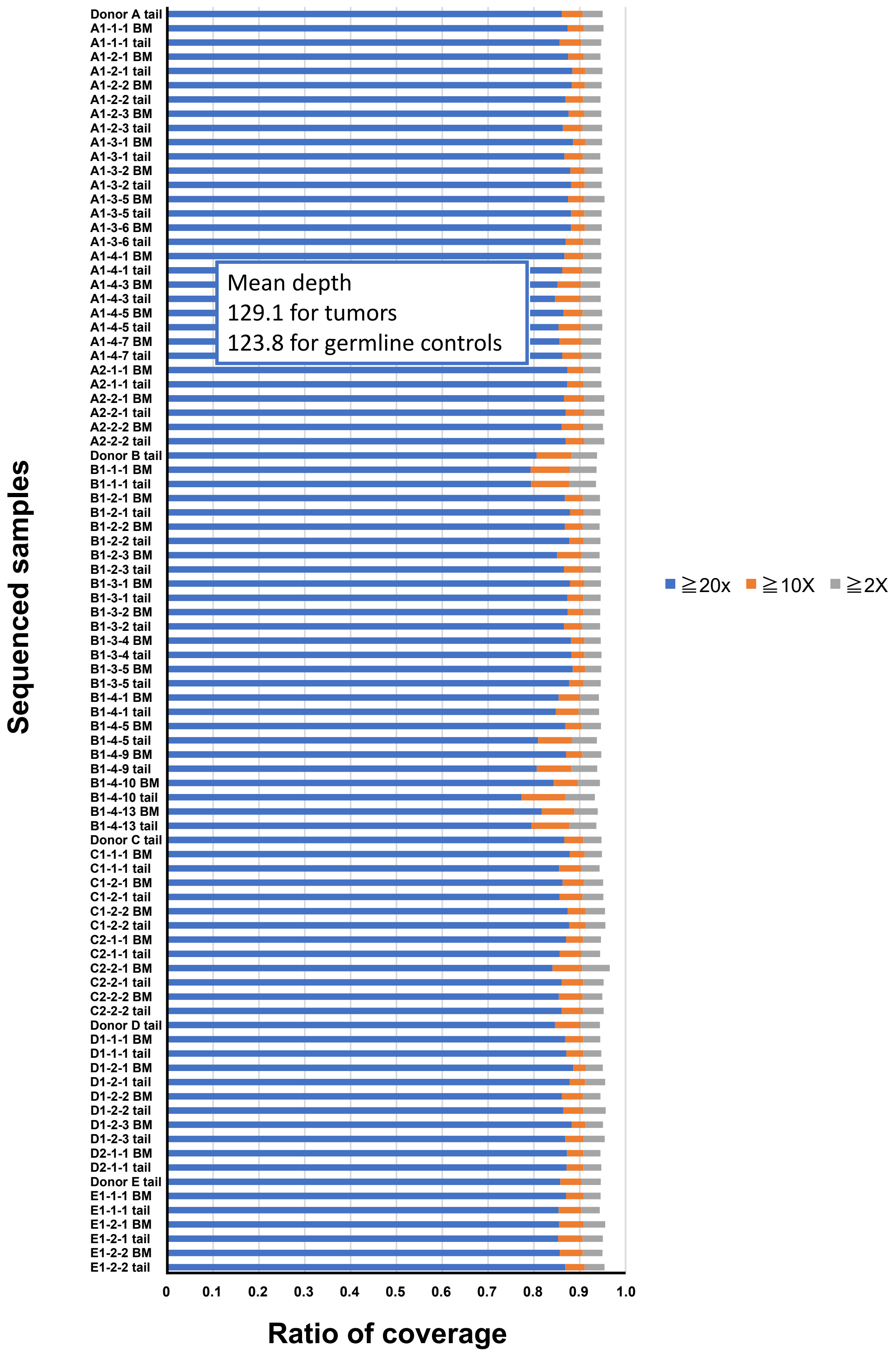

Supplementary Figure 5. Mean coverage of whole exome sequencing.
The coverage of the targeted region is plotted for 42 paired tumor/germline samples from transplanted mice and germline samples from 5 donor mice. The mean depth for all tumor (bone marrow: BM) and germline (tail) samples is also indicated.

Supplementary Figure 6

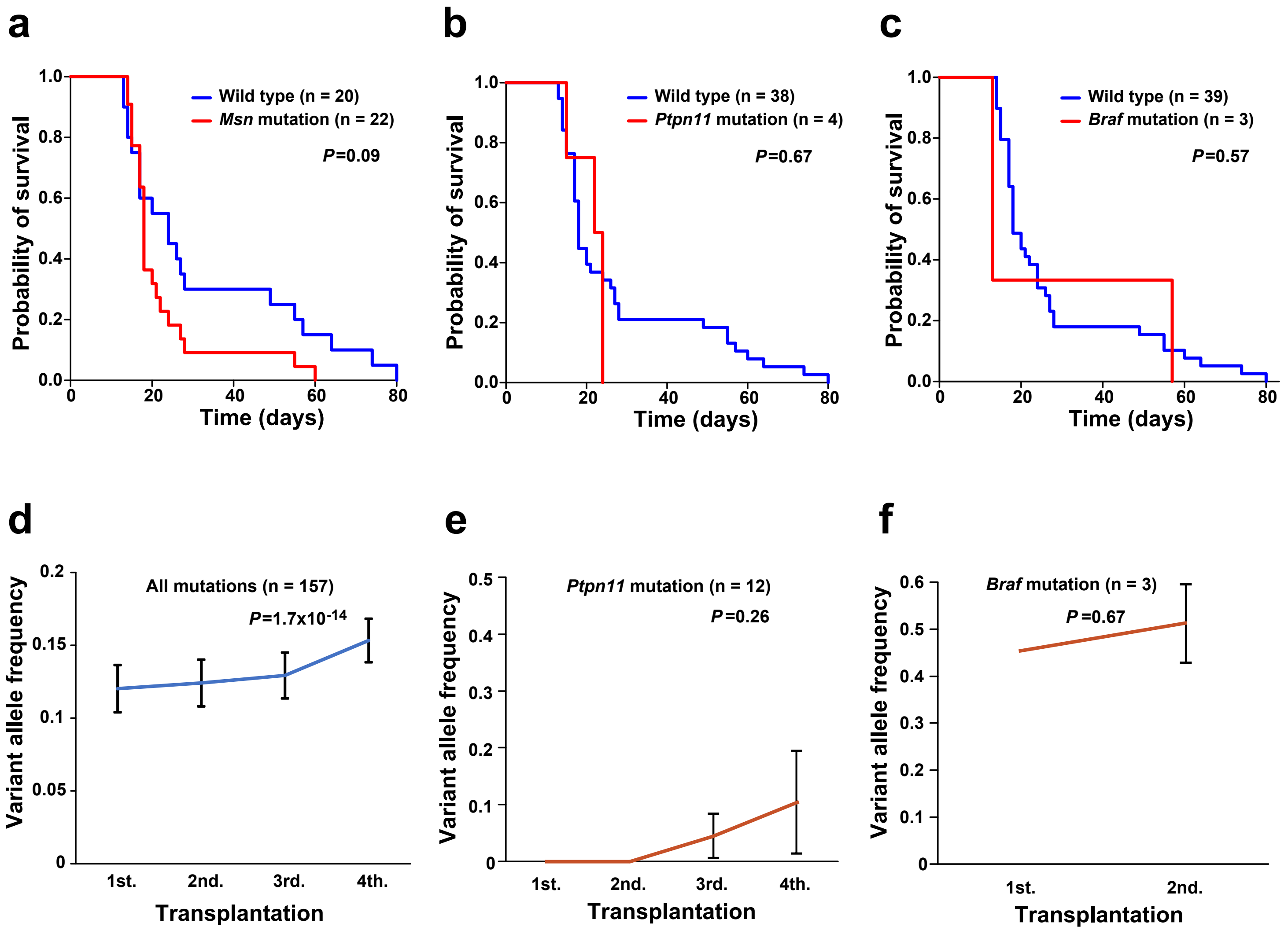

Supplementary Figure 6. Prognostic impact and allelic burden of identified mutations.
(**a-c**) Comparison of leukemia-onset time between cases with and without mutations in *Msn* (**a**), *Ptpn11* (**b**), and *Braf* (**c**). (**d-f**) Assessment of trend in variant allele frequencies of all mutations (**d**), *Ptpn11* (**e**), and *Braf* (**f**).

Supplementary Figure 7

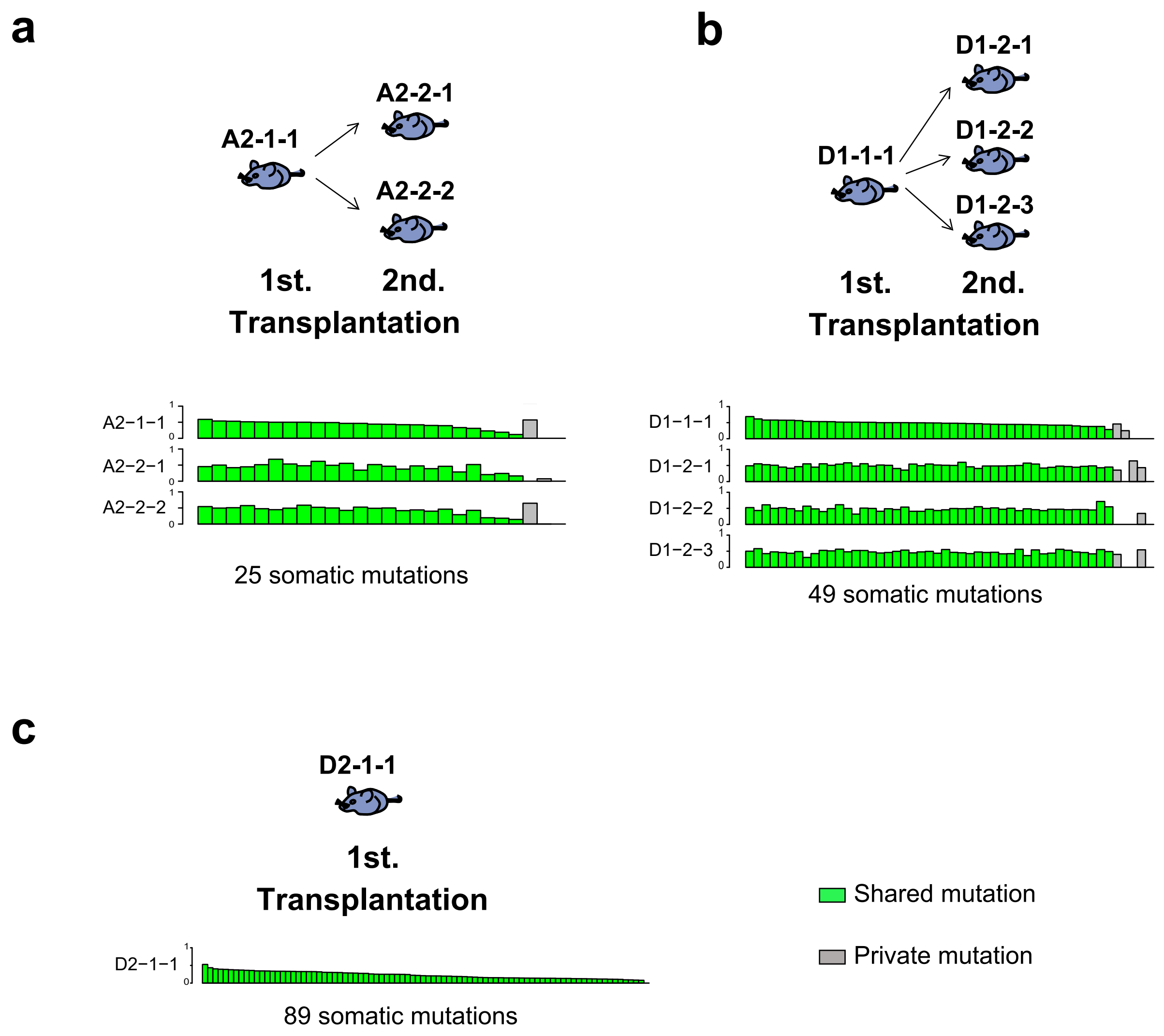

Supplementary Figure 7. Landscape of somatic mutations in transplantation model of mouse *MLL/AF9*-AML.

Upper panels show mice analyzed by WES in strain A2 (**a**), D1 (**b**), and D2 (**c**). Lower panels demonstrate landscape and number of somatic mutations in each strain. Green and gray indicate shared and private mutations, respectively.

Supplementary Figure 8

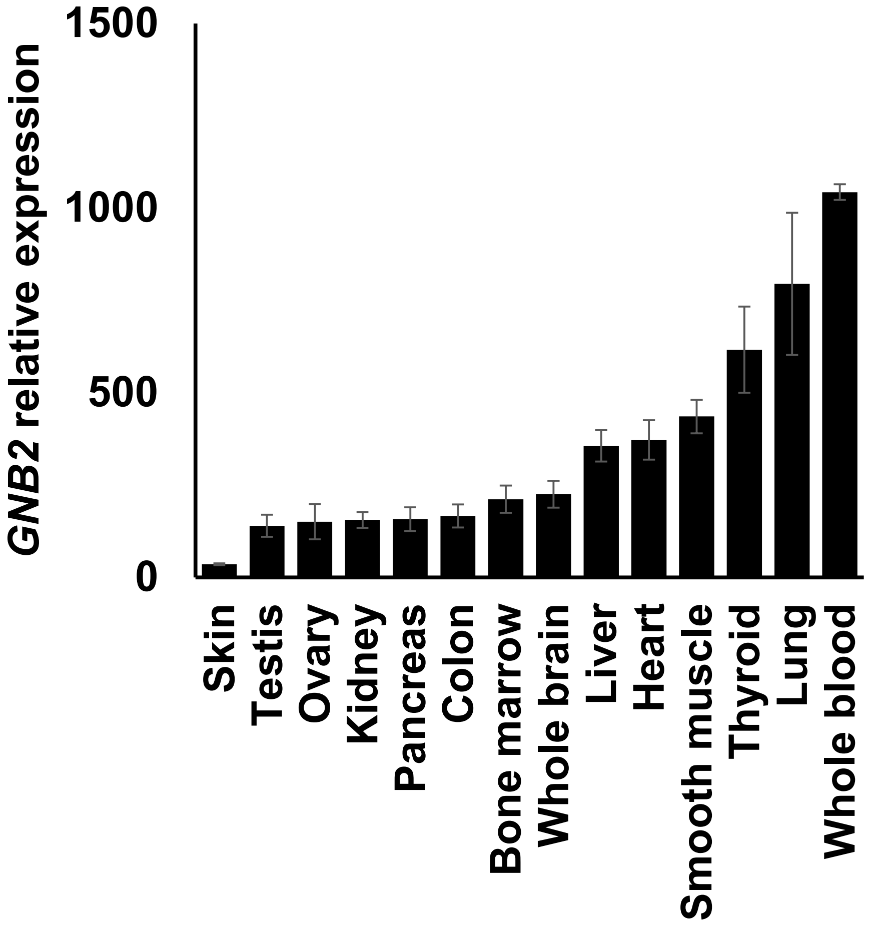

Supplementary Figure 8. *GNB2* relative expression in human normal tissues.
Relative expressions of *GNB2* mRNA in various human normal tissues were extracted from the global gene expression data set analyzed by GeneAtlas U133A expression array, which was downloaded from BioGPS (http://biogps.org/).

Supplementary Figure 9

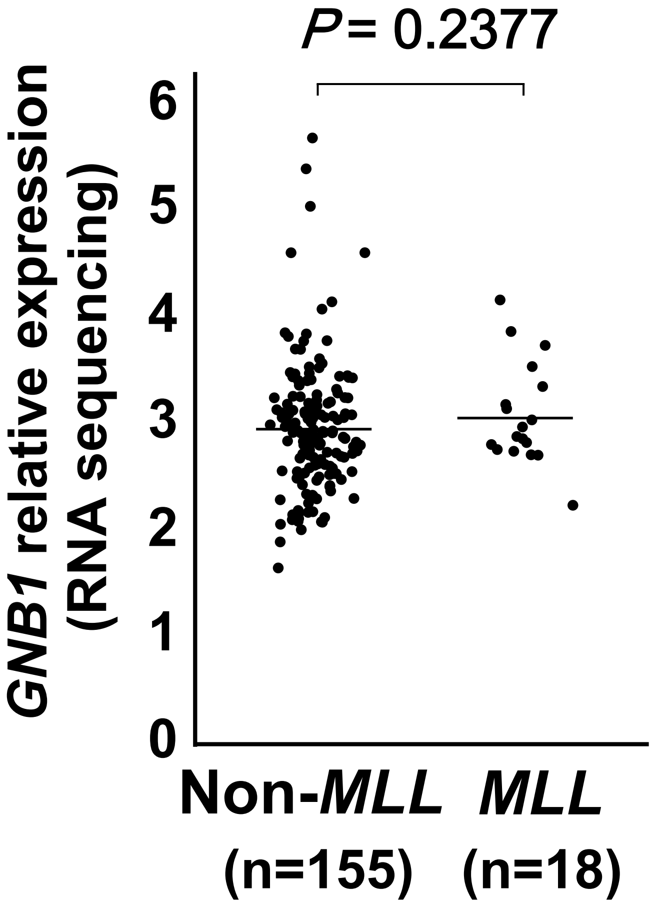

Supplementary Figure 9. *GNB1* relative expression in human MLL-AML.
GNB1 relative expression in human AML with and without *MLL*-fusion gene (RNA sequencing).

Supplementary Figure 10

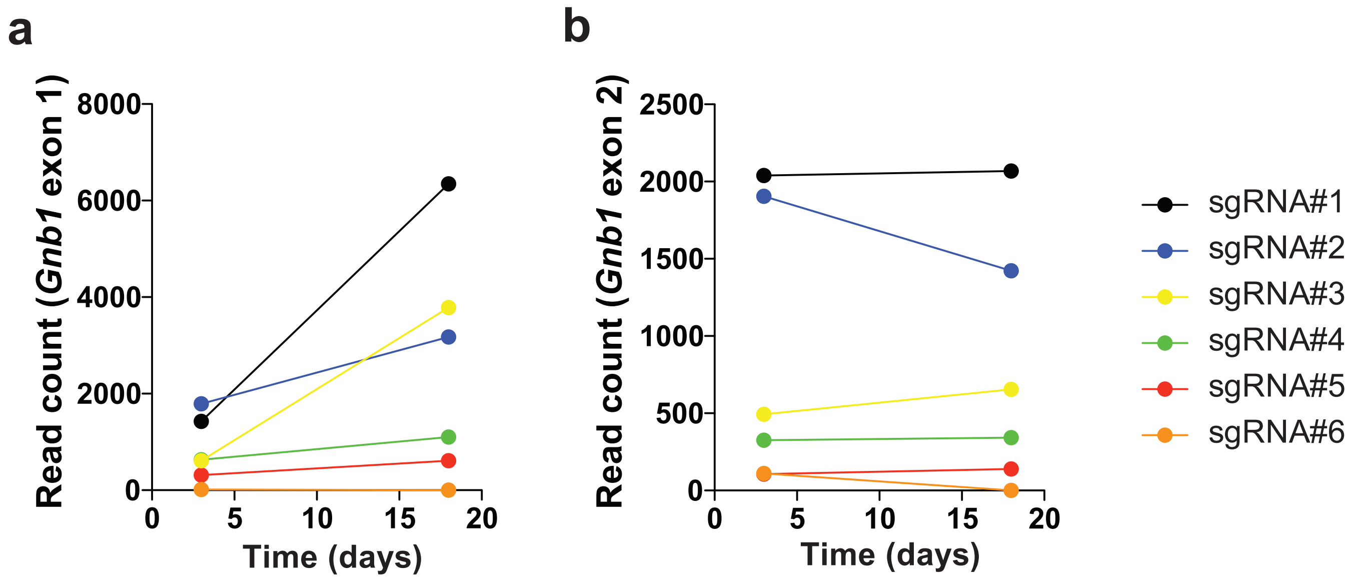

Supplementary Figure 10. Impact of depletion of *Gnb1* in mouse *MLL*/*AF9* acute myeloid leukemia. By genome-wide CRISPR-Cas9 screens, read counts of six sgRNAs targeting per exon (exon 1 (a) and 2 (b)) in *Gnb1* were measured after a 16-day incubation period to confirm potential oncogenic function of Gnb1 in *MLL*-AML.

Supplementary Table 1. Primer sets for real time RT-PCR

**Primer annotation**	Primer sequence
GNB2 (NM_005273)	
Forward primer	TGAATCCGACATCAATGCAG
Reverse primer	TCAGAGCCGGTGGTGAAG
Gnb2 (NM_010312)	
Forward primer	GGCAAAAATCTATGCCATGC
Reverse primer	TGATGAGCTTTCCGTCCTG
Gnb1 (NM_001160017)	
Forward primer	CCAGTTCTGGAGACACCACAT
Reverse primer	GGAGCAAGAGACAGGCTCA
18S	
Forward primer	GTAACCCGTTGAACCCCATT
Reverse primer	CCATCCAATCGGTAGTAGCG

Supplementary Table 2. Targeted sequences of shRNA for *GNB2* knockdown

Target gene	shRNA #	Targeted sequence
GNB2	#2	CCTGGATGACAACCAAATCAT
GNB2	#5	CGACGACTTCAACTGCAACAT
GNB2	#6	GCCAATTCCAACTCTGTAAAA
Scramble	-	CCTAAGGTTAAGTCGCCCTCG

Effect of shRNA #6 was shown in **Figure 8a-c**. Results of #2 and #5 were not shown.

Supplementary Table 3. Somatic mutations identified by whole exome sequencing in mouse *MLL/AF9* leukemic cells (n = 42).

Strain	Mouse	Chromosome	Start	End	Reference	Alteration	Gene	Mutation type	Accession number	Nucleotide	Amino acid	Variant allele frequency
A1	A1-1-1	chr1	110925165	110925165	G	A	Cdh19	Missense	NM_001081386	c.C1039T	p.H347Y	0.302
A1	A1-1-1	chr1	88215102	88215102	G	A	Ugt1a1	Missense	NM_201645	c.G929A	p.G310E	0.099
A1	A1-1-1	chr1	63309706	63309706	G	A	Zdbf2	Missense	NM_001267872	c.G7243A	p.A2415T	0.095
A1	A1-1-1	chr2	93696152	93696152	G	A	Ext2	Missense	NM_010163	c.C2138T	p.P713L	0.517
A1	A1-1-1	chr2	155421512	155421512	G	A	Ncoa6	Missense	NM_019825	c.C1001T	p.P334L	0.593
A1	A1-1-1	chr2	124664020	124664020	G	A	Sema6d	Splice site	NM_199241	c.1934-1G>A	NA	0.426
A1	A1-1-1	chr2	59838975	59838975	G	A	Tanc1	Missense	NM_198294	c.G3803A	p.G1268E	0.452
A1	A1-1-1	chr3	106149750	106149750	T	C	Chi3l3	Missense	NM_009892	c.A877G	p.K293E	0.266
A1	A1-1-1	chr4	63225586	63225586	T	G	Col27a1	Missense	NM_025685	c.T1510G	p.L504V	0.461
A1	A1-1-1	chr5	120609628	120609628	G	A	1110008J03Rik	Missense	NM_029096	c.C604T	p.P202S	0.491
A1	A1-1-1	chr5	14539711	14539711	C	T	Pclo	Missense	NM_011995	c.C2024T	p.A675V	0.321
A1	A1-1-1	chr6	38196439	38196439	G	A	D630045J12Rik	Missense	NM_194061	c.C793T	p.L265F	0.448
A1	A1-1-1	chr6	85337030	85337030	G	A	Rab11fip5	Missense	NM_001003955	c.C3950T	p.P1317L	0.416
A1	A1-1-1	chr7	128133275	128133275	T	C	Itgax	Missense	NM_021334	c.T572C	p.L191P	0.435
A1	A1-1-1	chr7	102752989	102752989	T	A	Olfr560	Missense	NM_147113	c.A939T	p.E313D	0.554
A1	A1-1-1	chr7	25081644	25081644	C	T	Zfp574	Missense	NM_001168506	c.C2090T	p.P697L	0.506
A1	A1-1-1	chr8	27154308	27154308	G	A	Rab11fip1	Missense	NM_001080813	c.C1448T	p.P483L	0.544
A1	A1-1-1	chr9	14572677	14572677	C	T	Amotl1	Missense	NM_001081395	c.G1589A	p.R530Q	0.382
A1	A1-1-1	chr9	54335830	54335830	T	A	Gldn	Nonsense	NM_177350	c.T1095A	p.Y365X	0.488
A1	A1-1-1	chr10	11295240	11295240	T	G	Fbxo30	Missense	NM_027968	c.T2061G	p.F687L	0.515
A1	A1-1-1	chr10	129912132	129912132	C	T	Olfr816	Missense	NM_146672	c.G145A	p.V49M	0.525
A1	A1-1-1	chr10	94794675	94794675	C	A	Plxnc1	Nonsense	NM_018797	c.G4456T	p.E1486X	0.5
A1	A1-1-1	chr10	51723856	51723856	C	T	Rfx6	Missense	NM_001159389	c.C1799T	p.P600L	0.552
A1	A1-1-1	chr10	26245602	26245602	T	C	Samd3	Missense	NM_001115154	c.T616C	p.F206L	0.456
A1	A1-1-1	chr10	33200204	33200204	C	A	Trdn	Missense	NM_029726	c.C829A	p.H277N	0.5
A1	A1-1-1	chr11	120273314	120273314	G	A	Bahcc1	Splice site	NM_198423	c.2228-1G>A	NA	0.48
A1	A1-1-1	chr11	106813834	106813834	G	A	Cep95	Missense	NM_177088	c.G1514A	p.R505K	0.439
A1	A1-1-1	chr11	54916905	54916905	G	A	Tnip1	Missense	NM_021327	c.C1709T	p.A570V	0.505
A1	A1-1-1	chr13	73268858	73268858	G	A	Irx4	Missense	NM_018885	c.G1372A	p.G458S	0.517
A1	A1-1-1	chr14	37057443	37057443	A	T	Lrit1	Missense	NM_146245	c.A332T	p.E111V	0.44
A1	A1-1-1	chr14	79770248	79770248	C	T	Pcdh8	Missense	NM_021543	c.G874A	p.E292K	0.504
A1	A1-1-1	chr14	57643200	57643200	G	A	Xpo4	Missense	NM_020506	c.C199T	p.L67F	0.319
A1	A1-1-1	chr15	76106727	76106727	C	T	Eppk1	Missense	NM_144848	c.G5953A	p.G1985S	0.397
A1	A1-1-1	chr15	52335160	52335160	G	T	Slc30a8	Missense	NM_172816	c.G1000T	p.A334S	0.429
A1	A1-1-1	chr17	65256583	65256583	C	T	Tmem232	Missense	NM_001008973	c.G1814A	p.R605Q	0.583
A1	A1-1-1	chr18	34863349	34863349	G	T	Egr1	Missense	NM_007913	c.G1183T	p.A395S	0.565
A1	A1-1-1	chr19	29717713	29717713	G	A	9930021J03Rik	Missense	NM_172836	c.C4580T	p.P1527L	0.424
A1	A1-1-1	chr19	54047310	54047310	G	A	Adra2a	Missense	NM_007417	c.G1096A	p.E366K	0.503
A1	A1-1-1	chrX	94538510	94538510	T	C	Maged1	Splice site	NM_019791	c.1414-2A>G	NA	0.434
A1	A1-1-1	chrX	139824102	139824102	C	T	Morc4	Missense	NM_001193309	c.G2213A	p.G738E	0.444
A1	A1-2-1	chr1	110925165	110925165	G	A	Cdh19	Missense	NM_001081386	c.C1039T	p.H347Y	0.489
A1	A1-2-1	chr2	93696152	93696152	G	A	Ext2	Missense	NM_010163	c.C2138T	p.P713L	0.449
A1	A1-2-1	chr2	155421512	155421512	G	A	Ncoa6	Missense	NM_019825	c.C1001T	p.P334L	0.516
A1	A1-2-1	chr2	124664020	124664020	G	A	Sema6d	Splice site	NM_199241	c.1934-1G>A	NA	0.486
A1	A1-2-1	chr2	59838975	59838975	G	A	Tanc1	Missense	NM_198294	c.G3803A	p.G1268E	0.303
A1	A1-2-1	chr3	106149750	106149750	T	C	Chi3l3	Missense	NM_009892	c.A877G	p.K293E	0.193
A1	A1-2-1	chr3	158161735	158161735	C	T	Lrrc7	Missense	NM_001081358	c.G2368A	p.A790T	0.086
A1	A1-2-1	chr4	63225586	63225586	T	G	Col27a1	Missense	NM_025685	c.T1510G	p.L504V	0.557
A1	A1-2-1	chr4	113177004	113177004	G	A	Skint6	Nonsense	NM_001103199	c.C925T	p.Q309X	0.537
A1	A1-2-1	chr5	120609628	120609628	G	A	1110008J03Rik	Missense	NM_029096	c.C604T	p.P202S	0.552
A1	A1-2-1	chr5	35043862	35043880	GTGGGCGACCACTGGGCCC	-	Hgfac	Nonsense	NM_019447	c.628_646del	p.G211X	0.242
A1	A1-2-1	chr6	38196439	38196439	G	A	D630045J12Rik	Missense	NM_194061	c.C793T	p.L265F	0.493
A1	A1-2-1	chr6	85337030	85337030	G	A	Rab11fip5	Missense	NM_001003955	c.C3950T	p.P1317L	0.5
A1	A1-2-1	chr7	128133275	128133275	T	C	Itgax	Missense	NM_021334	c.T572C	p.L191P	0.545
A1	A1-2-1	chr7	102752989	102752989	T	A	Olfr560	Missense	NM_147113	c.A939T	p.E313D	0.366
A1	A1-2-1	chr7	25081644	25081644	C	T	Zfp574	Missense	NM_001168506	c.C2090T	p.P697L	0.482
A1	A1-2-1	chr8	27154308	27154308	G	A	Rab11fip1	Missense	NM_001080813	c.C1448T	p.P483L	0.489
A1	A1-2-1	chr9	14572677	14572677	C	T	Amotl1	Missense	NM_001081395	c.G1589A	p.R530Q	0.089
A1	A1-2-1	chr9	54335830	54335830	T	A	Gldn	Nonsense	NM_177350	c.T1095A	p.Y365X	0.404
A1	A1-2-1	chr10	11295240	11295240	T	G	Fbxo30	Missense	NM_027968	c.T2061G	p.F687L	0.447
A1	A1-2-1	chr10	129912132	129912132	C	T	Olfr816	Missense	NM_146672	c.G145A	p.V49M	0.514
A1	A1-2-1	chr10	94794675	94794675	C	A	Plxnc1	Nonsense	NM_018797	c.G4456T	p.E1486X	0.574
A1	A1-2-1	chr10	51723856	51723856	C	T	Rfx6	Missense	NM_001159389	c.C1799T	p.P600L	0.423
A1	A1-2-1	chr10	26245602	26245602	T	C	Samd3	Missense	NM_001115154	c.T616C	p.F206L	0.473
A1	A1-2-1	chr10	33200204	33200204	C	A	Trdn	Missense	NM_029726	c.C829A	p.H277N	0.5
A1	A1-2-1	chr11	120273314	120273314	G	A	Bahcc1	Splice site	NM_198423	c.2228-1G>A	NA	0.483
A1	A1-2-1	chr11	106813834	106813834	G	A	Cep95	Missense	NM_177088	c.G1514A	p.R505K	0.585
A1	A1-2-1	chr11	54916905	54916905	G	A	Tnip1	Missense	NM_021327	c.C1709T	p.A570V	0.494
A1	A1-2-1	chr13	73268858	73268858	G	A	Irx4	Missense	NM_018885	c.G1372A	p.G458S	0.498
A1	A1-2-1	chr14	37057443	37057443	A	T	Lrit1	Missense	NM_146245	c.A332T	p.E111V	0.482
A1	A1-2-1	chr14	79770248	79770248	C	T	Pcdh8	Missense	NM_021543	c.G874A	p.E292K	0.527
A1	A1-2-1	chr14	57643200	57643200	G	A	Xpo4	Missense	NM_020506	c.C199T	p.L67F	0.528
A1	A1-2-1	chr15	76106727	76106727	C	T	Eppk1	Missense	NM_144848	c.G5953A	p.G1985S	0.475
A1	A1-2-1	chr15	52335160	52335160	G	T	Slc30a8	Missense	NM_172816	c.G1000T	p.A334S	0.5
A1	A1-2-1	chr15	89116046	89116046	C	T	Tubgcp6	Missense	NM_001163319	c.G1115A	p.R372Q	0.07
A1	A1-2-1	chr17	65256583	65256583	C	T	Tmem232	Missense	NM_001008973	c.G1814A	p.R605Q	0.462
A1	A1-2-1	chr18	34863349	34863349	G	T	Egr1	Missense	NM_007913	c.G1183T	p.A395S	0.526
A1	A1-2-1	chr19	29717713	29717713	G	A	9930021J03Rik	Missense	NM_172836	c.C4580T	p.P1527L	0.497
A1	A1-2-1	chr19	54047310	54047310	G	A	Adra2a	Missense	NM_007417	c.G1096A	p.E366K	0.483
A1	A1-2-1	chrX	94538510	94538510	T	C	Maged1	Splice site	NM_019791	c.1414-2A>G	NA	0.431
A1	A1-2-1	chrX	139824102	139824102	C	T	Morc4	Missense	NM_001193309	c.G2213A	p.G738E	0.481
A1	A1-2-1	chrX	96160175	96160175	C	T	Msn	Missense	NM_010833	c.C883T	p.R295C	0.405
A1	A1-2-2	chr1	110925165	110925165	G	A	Cdh19	Missense	NM_001081386	c.C1039T	p.H347Y	0.478
A1	A1-2-2	chr2	93696152	93696152	G	A	Ext2	Missense	NM_010163	c.C2138T	p.P713L	0.495
A1	A1-2-2	chr2	155421512	155421512	G	A	Ncoa6	Missense	NM_019825	c.C1001T	p.P334L	0.5
A1	A1-2-2	chr2	124664020	124664020	G	A	Sema6d	Splice site	NM_199241	c.1934-1G>A	NA	0.456
A1	A1-2-2	chr2	59838975	59838975	G	A	Tanc1	Missense	NM_198294	c.G3803A	p.G1268E	0.5
A1	A1-2-2	chr3	106149750	106149750	T	C	Chi3l3	Missense	NM_009892	c.A877G	p.K293E	0.207
A1	A1-2-2	chr4	63225586	63225586	T	G	Col27a1	Missense	NM_025685	c.T1510G	p.L504V	0.52
A1	A1-2-2	chr5	120609628	120609628	G	A	1110008J03Rik	Missense	NM_029096	c.C604T	p.P202S	0.485
A1	A1-2-2	chr5	35043862	35043880	GTGGGCGACCACTGGGCCC	-	Hgfac	Nonsense	NM_019447	c.628_646del	p.G211X	0.314
A1	A1-2-2	chr5	14539711	14539711	C	T	Pclo	Missense	NM_011995	c.C2024T	p.A675V	0.11
A1	A1-2-2	chr6	38196439	38196439	G	A	D630045J12Rik	Missense	NM_194061	c.C793T	p.L265F	0.514
A1	A1-2-2	chr6	85337030	85337030	G	A	Rab11fip5	Missense	NM_001003955	c.C3950T	p.P1317L	0.393
A1	A1-2-2	chr7	128133275	128133275	T	C	Itgax	Missense	NM_021334	c.T572C	p.L191P	0.497
A1	A1-2-2	chr7	102752989	102752989	T	A	Olfr560	Missense	NM_147113	c.A939T	p.E313D	0.483
A1	A1-2-2	chr7	25081644	25081644	C	T	Zfp574	Missense	NM_001168506	c.C2090T	p.P697L	0.502
A1	A1-2-2	chr8	27154308	27154308	G	A	Rab11fip1	Missense	NM_001080813	c.C1448T	p.P483L	0.45
A1	A1-2-2	chr9	14572677	14572677	C	T	Amotl1	Missense	NM_001081395	c.G1589A	p.R530Q	0.125
A1	A1-2-2	chr9	54335830	54335830	T	A	Gldn	Nonsense	NM_177350	c.T1095A	p.Y365X	0.5
A1	A1-2-2	chr10	11295240	11295240	T	G	Fbxo30	Missense	NM_027968	c.T2061G	p.F687L	0.525
A1	A1-2-2	chr10	129912132	129912132	C	T	Olfr816	Missense	NM_146672	c.G145A	p.V49M	0.344
A1	A1-2-2	chr10	94794675	94794675	C	A	Plxnc1	Nonsense	NM_018797	c.G4456T	p.E1486X	0.5
A1	A1-2-2	chr10	51723856	51723856	C	T	Rfx6	Missense	NM_001159389	c.C1799T	p.P600L	0.54
A1	A1-2-2	chr10	26245602	26245602	T	C	Samd3	Missense	NM_001115154	c.T616C	p.F206L	0.556
A1	A1-2-2	chr10	33200204	33200204	C	A	Trdn	Missense	NM_029726	c.C829A	p.H277N	0.478
A1	A1-2-2	chr11	120273314	120273314	G	A	Bahcc1	Splice site	NM_198423	c.2228-1G>A	NA	0.525
A1	A1-2-2	chr11	106813834	106813834	G	A	Cep95	Missense	NM_177088	c.G1514A	p.R505K	0.609
A1	A1-2-2	chr11	54916905	54916905	G	A	Tnip1	Missense	NM_021327	c.C1709T	p.A570V	0.47
A1	A1-2-2	chr13	73268858	73268858	G	A	Irx4	Missense	NM_018885	c.G1372A	p.G458S	0.529
A1	A1-2-2	chr13	73540922	73540922	C	A	Slc6a3	Missense	NM_010020	c.C364A	p.Q122K	0.131
A1	A1-2-2	chr14	37057443	37057443	A	T	Lrit1	Missense	NM_146245	c.A332T	p.E111V	0.461
A1	A1-2-2	chr14	79770248	79770248	C	T	Pcdh8	Missense	NM_021543	c.G874A	p.E292K	0.473
A1	A1-2-2	chr14	57643200	57643200	G	A	Xpo4	Missense	NM_020506	c.C199T	p.L67F	0.345
A1	A1-2-2	chr15	76106727	76106727	C	T	Eppk1	Missense	NM_144848	c.G5953A	p.G1985S	0.424
A1	A1-2-2	chr15	52335160	52335160	G	T	Slc30a8	Missense	NM_172816	c.G1000T	p.A334S	0.546
A1	A1-2-2	chr17	65256583	65256583	C	T	Tmem232	Missense	NM_001008973	c.G1814A	p.R605Q	0.425
A1	A1-2-2	chr18	34863349	34863349	G	T	Egr1	Missense	NM_007913	c.G1183T	p.A395S	0.498
A1	A1-2-2	chr19	29717713	29717713	G	A	9930021J03Rik	Missense	NM_172836	c.C4580T	p.P1527L	0.451
A1	A1-2-2	chr19	54047310	54047310	G	A	Adra2a	Missense	NM_007417	c.G1096A	p.E366K	0.482
A1	A1-2-2	chrX	94538510	94538510	T	C	Maged1	Splice site	NM_019791	c.1414-2A>G	NA	0.391
A1	A1-2-2	chrX	139824102	139824102	C	T	Morc4	Missense	NM_001193309	c.G2213A	p.G738E	0.509

A1	A1-2-2	chrX	96160175	96160175	C	T	Msn	Missense	NM_010833	c.C883T	p.R295C	0.351
A1	A1-2-3	chr1	110925165	110925165	G	A	Cdh19	Missense	NM_001081386	c.C1039T	p.H347Y	0.591
A1	A1-2-3	chr1	63309706	63309706	G	A	Zdbf2	Missense	NM_001267872	c.G7243A	p.A2415T	0.102
A1	A1-2-3	chr2	93696152	93696152	G	A	Ext2	Missense	NM_010163	c.C2138T	p.P713L	0.418
A1	A1-2-3	chr2	155421512	155421512	G	A	Ncoa6	Missense	NM_019825	c.C1001T	p.P334L	0.355
A1	A1-2-3	chr2	124664020	124664020	G	A	Sema6d	Splice site	NM_199241	c.1934-1G>A	NA	0.431
A1	A1-2-3	chr2	59838975	59838975	G	A	Tanc1	Missense	NM_198294	c.G3803A	p.G1268E	0.514
A1	A1-2-3	chr3	106149750	106149750	T	C	Chi3l3	Missense	NM_009892	c.A877G	p.K293E	0.254
A1	A1-2-3	chr4	63225586	63225586	T	G	Col27a1	Missense	NM_025685	c.T1510G	p.L504V	0.538
A1	A1-2-3	chr4	113177004	113177004	G	A	Skint6	Nonsense	NM_001103199	c.C925T	p.Q309X	0.415
A1	A1-2-3	chr5	120609628	120609628	G	A	1110008J03Rik	Missense	NM_029096	c.C604T	p.P202S	0.479
A1	A1-2-3	chr5	35043862	35043880	GTGGGCGACCACTGGGCCC	-	Hgfac	Nonsense	NM_019447	c.628_646del	p.G211X	0.077
A1	A1-2-3	chr5	14539711	14539711	C	T	Pclo	Missense	NM_011995	c.C2024T	p.A675V	0.195
A1	A1-2-3	chr6	38196439	38196439	G	A	D630045J12Rik	Missense	NM_194061	c.C793T	p.L265F	0.459
A1	A1-2-3	chr6	85337030	85337030	G	A	Rab11fip5	Missense	NM_001003955	c.C3950T	p.P1317L	0.42
A1	A1-2-3	chr7	128133275	128133275	T	C	Itgax	Missense	NM_021334	c.T572C	p.L191P	0.441
A1	A1-2-3	chr7	102752989	102752989	T	A	Olfr560	Missense	NM_147113	c.A939T	p.E313D	0.52
A1	A1-2-3	chr7	25081644	25081644	C	T	Zfp574	Missense	NM_001168506	c.C2090T	p.P697L	0.438
A1	A1-2-3	chr8	27154308	27154308	G	A	Rab11fip1	Missense	NM_001080813	c.C1448T	p.P483L	0.549
A1	A1-2-3	chr9	14572677	14572677	C	T	Amotl1	Missense	NM_001081395	c.G1589A	p.R530Q	0.305
A1	A1-2-3	chr9	54335830	54335830	T	A	Gldn	Nonsense	NM_177350	c.T1095A	p.Y365X	0.43
A1	A1-2-3	chr10	11295240	11295240	T	G	Fbxo30	Missense	NM_027968	c.T2061G	p.F687L	0.302
A1	A1-2-3	chr10	129912132	129912132	C	T	Olfr816	Missense	NM_146672	c.G145A	p.V49M	0.438
A1	A1-2-3	chr10	94794675	94794675	C	A	Plxnc1	Nonsense	NM_018797	c.G4456T	p.E1486X	0.52
A1	A1-2-3	chr10	51723856	51723856	C	T	Rfx6	Missense	NM_001159389	c.C1799T	p.P600L	0.51
A1	A1-2-3	chr10	26245602	26245602	T	C	Samd3	Missense	NM_001115154	c.T616C	p.F206L	0.533
A1	A1-2-3	chr10	33200204	33200204	C	A	Trdn	Missense	NM_029726	c.C829A	p.H277N	0.457
A1	A1-2-3	chr11	120273314	120273314	G	A	Bahcc1	Splice site	NM_198423	c.2228-1G>A	NA	0.484
A1	A1-2-3	chr11	106813834	106813834	G	A	Cep95	Missense	NM_177088	c.G1514A	p.R505K	0.452
A1	A1-2-3	chr11	54916905	54916905	G	A	Tnip1	Missense	NM_021327	c.C1709T	p.A570V	0.508
A1	A1-2-3	chr13	73268858	73268858	G	A	Irx4	Missense	NM_018885	c.G1372A	p.G458S	0.465
A1	A1-2-3	chr14	37057443	37057443	A	T	Lrit1	Missense	NM_146245	c.A332T	p.E111V	0.524
A1	A1-2-3	chr14	79770248	79770248	C	T	Pcdh8	Missense	NM_021543	c.G874A	p.E292K	0.532
A1	A1-2-3	chr14	57643200	57643200	G	A	Xpo4	Missense	NM_020506	c.C199T	p.L67F	0.5
A1	A1-2-3	chr15	76106727	76106727	C	T	Eppk1	Missense	NM_144848	c.G5953A	p.G1985S	0.487
A1	A1-2-3	chr15	52335160	52335160	G	T	Slc30a8	Missense	NM_172816	c.G1000T	p.A334S	0.514
A1	A1-2-3	chr17	65256583	65256583	C	T	Tmem232	Missense	NM_001008973	c.G1814A	p.R605Q	0.406
A1	A1-2-3	chr18	34863349	34863349	G	T	Egr1	Missense	NM_007913	c.G1183T	p.A395S	0.483
A1	A1-2-3	chr19	29717713	29717713	G	A	9930021J03Rik	Missense	NM_172836	c.C4580T	p.P1527L	0.452
A1	A1-2-3	chr19	54047310	54047310	G	A	Adra2a	Missense	NM_007417	c.G1096A	p.E366K	0.459
A1	A1-2-3	chrX	94538510	94538510	T	C	Maged1	Splice site	NM_019791	c.1414-2A>G	NA	0.463
A1	A1-2-3	chrX	139824102	139824102	C	T	Morc4	Missense	NM_001193309	c.G2213A	p.G738E	0.517
A1	A1-2-3	chrX	96160175	96160175	C	T	Msn	Missense	NM_010833	c.C883T	p.R295C	0.151
A1	A1-3-1	chr1	154472253	154472253	G	A	Cacna1e	Nonsense	NM_009782	c.C2761T	p.R921X	0.195
A1	A1-3-1	chr1	110925165	110925165	G	A	Cdh19	Missense	NM_001081386	c.C1039T	p.H347Y	0.475
A1	A1-3-1	chr2	93696152	93696152	G	A	Ext2	Missense	NM_010163	c.C2138T	p.P713L	0.354
A1	A1-3-1	chr2	40596939	40596939	C	T	Lrp1b	Missense	NM_053011	c.G13795A	p.A4599T	0.239
A1	A1-3-1	chr2	155421512	155421512	G	A	Ncoa6	Missense	NM_019825	c.C1001T	p.P334L	0.385
A1	A1-3-1	chr2	124664020	124664020	G	A	Sema6d	Splice site	NM_199241	c.1934-1G>A	NA	0.549
A1	A1-3-1	chr2	59838975	59838975	G	A	Tanc1	Missense	NM_198294	c.G3803A	p.G1268E	0.429
A1	A1-3-1	chr3	106149750	106149750	T	C	Chi3l3	Missense	NM_009892	c.A877G	p.K293E	0.246
A1	A1-3-1	chr4	63225586	63225586	T	G	Col27a1	Missense	NM_025685	c.T1510G	p.L504V	0.446
A1	A1-3-1	chr5	120609628	120609628	G	A	1110008J03Rik	Missense	NM_029096	c.C604T	p.P202S	0.479
A1	A1-3-1	chr6	38196439	38196439	G	A	D630045J12Rik	Missense	NM_194061	c.C793T	p.L265F	0.527
A1	A1-3-1	chr6	85337030	85337030	G	A	Rab11fip5	Missense	NM_001003955	c.C3950T	p.P1317L	0.639
A1	A1-3-1	chr7	128133275	128133275	T	C	Itgax	Missense	NM_021334	c.T572C	p.L191P	0.492
A1	A1-3-1	chr7	102752989	102752989	T	A	Olfr560	Missense	NM_147113	c.A939T	p.E313D	0.533
A1	A1-3-1	chr7	25081644	25081644	C	T	Zfp574	Missense	NM_001168506	c.C2090T	p.P697L	0.52
A1	A1-3-1	chr8	94941666	94941666	C	T	Gpr114	Missense	NM_001145972	c.C1358T	p.A453V	0.133
A1	A1-3-1	chr8	27154308	27154308	G	A	Rab11fip1	Missense	NM_001080813	c.C1448T	p.P483L	0.458
A1	A1-3-1	chr9	14572677	14572677	C	T	Amotl1	Missense	NM_001081395	c.G1589A	p.R530Q	0.202
A1	A1-3-1	chr9	62432454	62432454	G	A	Coro2b	Missense	NM_175484	c.C386T	p.A129V	0.107
A1	A1-3-1	chr9	54335830	54335830	T	A	Gldn	Nonsense	NM_177350	c.T1095A	p.Y365X	0.417
A1	A1-3-1	chr10	11295240	11295240	T	G	Fbxo30	Missense	NM_027968	c.T2061G	p.F687L	0.483
A1	A1-3-1	chr10	129912132	129912132	C	T	Olfr816	Missense	NM_146672	c.G145A	p.V49M	0.389
A1	A1-3-1	chr10	94794675	94794675	C	A	Plxnc1	Nonsense	NM_018797	c.G4456T	p.E1486X	0.536
A1	A1-3-1	chr10	51723856	51723856	C	T	Rfx6	Missense	NM_001159389	c.C1799T	p.P600L	0.438
A1	A1-3-1	chr10	26245602	26245602	T	C	Samd3	Missense	NM_001115154	c.T616C	p.F206L	0.558
A1	A1-3-1	chr10	33200204	33200204	C	A	Trdn	Missense	NM_029726	c.C829A	p.H277N	0.533
A1	A1-3-1	chr11	120273314	120273314	G	A	Bahcc1	Splice site	NM_198423	c.2228-1G>A	NA	0.476
A1	A1-3-1	chr11	106813834	106813834	G	A	Cep95	Missense	NM_177088	c.G1514A	p.R505K	0.438
A1	A1-3-1	chr11	73847215	73847216	TC	AT	Olfr393	Missense	NM_147008	c.GA908_909AT	p.R303N	0.151
A1	A1-3-1	chr11	54916905	54916905	G	A	Tnip1	Missense	NM_021327	c.C1709T	p.A570V	0.47
A1	A1-3-1	chr13	73268858	73268858	G	A	Irx4	Missense	NM_018885	c.G1372A	p.G458S	0.531
A1	A1-3-1	chr14	49774037	49774037	G	C	3632451O06Rik	Missense	NM_026142	c.C212G	p.T71S	0.136
A1	A1-3-1	chr14	37057443	37057443	A	T	Lrit1	Missense	NM_146245	c.A332T	p.E111V	0.451
A1	A1-3-1	chr14	79770248	79770248	C	T	Pcdh8	Missense	NM_021543	c.G874A	p.E292K	0.529
A1	A1-3-1	chr14	70440016	70440016	G	A	Polr3d	Missense	NM_001164082	c.C919T	p.R307W	0.292
A1	A1-3-1	chr14	57643200	57643200	G	A	Xpo4	Missense	NM_020506	c.C199T	p.L67F	0.42
A1	A1-3-1	chr14	55063007	55063007	C	T	Zfhx2	Missense	NM_001039198	c.G7286A	p.S2429N	0.216
A1	A1-3-1	chr15	76106727	76106727	C	T	Eppk1	Missense	NM_144848	c.G5953A	p.G1985S	0.489
A1	A1-3-1	chr15	52335160	52335160	G	T	Slc30a8	Missense	NM_172816	c.G1000T	p.A334S	0.47
A1	A1-3-1	chr17	65256583	65256583	C	T	Tmem232	Missense	NM_001008973	c.G1814A	p.R605Q	0.452
A1	A1-3-1	chr18	34863349	34863349	G	T	Egr1	Missense	NM_007913	c.G1183T	p.A395S	0.522
A1	A1-3-1	chr19	29717713	29717713	G	A	9930021J03Rik	Missense	NM_172836	c.C4580T	p.P1527L	0.455
A1	A1-3-1	chr19	54047310	54047310	G	A	Adra2a	Missense	NM_007417	c.G1096A	p.E366K	0.501
A1	A1-3-1	chrX	94538510	94538510	T	C	Maged1	Splice site	NM_019791	c.1414-2A>G	NA	0.326
A1	A1-3-1	chrX	139824102	139824102	C	T	Morc4	Missense	NM_001193309	c.G2213A	p.G738E	0.466
A1	A1-3-1	chrX	96160175	96160175	C	T	Msn	Missense	NM_010833	c.C883T	p.R295C	0.105
A1	A1-3-2	chr1	154472253	154472253	G	A	Cacna1e	Nonsense	NM_009782	c.C2761T	p.R921X	0.175
A1	A1-3-2	chr1	110925165	110925165	G	A	Cdh19	Missense	NM_001081386	c.C1039T	p.H347Y	0.508
A1	A1-3-2	chr1	88215102	88215102	G	A	Ugt1a1	Missense	NM_201645	c.G929A	p.G310E	0.078
A1	A1-3-2	chr2	93696152	93696152	G	A	Ext2	Missense	NM_010163	c.C2138T	p.P713L	0.416
A1	A1-3-2	chr2	151970755	151970755	G	A	Fam110a	Missense	NM_028666	c.C94T	p.P32S	0.17
A1	A1-3-2	chr2	40596939	40596939	C	T	Lrp1b	Missense	NM_053011	c.G13795A	p.A4599T	0.129
A1	A1-3-2	chr2	155421512	155421512	G	A	Ncoa6	Missense	NM_019825	c.C1001T	p.P334L	0.5
A1	A1-3-2	chr2	124664020	124664020	G	A	Sema6d	Splice site	NM_199241	c.1934-1G>A	NA	0.536
A1	A1-3-2	chr2	59838975	59838975	G	A	Tanc1	Missense	NM_198294	c.G3803A	p.G1268E	0.478
A1	A1-3-2	chr3	106149750	106149750	T	C	Chi3l3	Missense	NM_009892	c.A877G	p.K293E	0.227
A1	A1-3-2	chr4	63225586	63225586	T	G	Col27a1	Missense	NM_025685	c.T1510G	p.L504V	0.46
A1	A1-3-2	chr4	56790899	56790899	C	A	Ikbkap	Missense	NM_026079	c.G853T	p.D285Y	0.19
A1	A1-3-2	chr5	120609628	120609628	G	A	1110008J03Rik	Missense	NM_029096	c.C604T	p.P202S	0.532
A1	A1-3-2	chr6	38196439	38196439	G	A	D630045J12Rik	Missense	NM_194061	c.C793T	p.L265F	0.38
A1	A1-3-2	chr6	85337030	85337030	G	A	Rab11fip5	Missense	NM_001003955	c.C3950T	p.P1317L	0.507
A1	A1-3-2	chr6	83151912	83151912	G	A	Rtkn	Missense	NM_001136227	c.G1387A	p.A463T	0.082
A1	A1-3-2	chr7	128133275	128133275	T	C	Itgax	Missense	NM_021334	c.T572C	p.L191P	0.513
A1	A1-3-2	chr7	102752989	102752989	T	A	Olfr560	Missense	NM_147113	c.A939T	p.E313D	0.555
A1	A1-3-2	chr7	25081644	25081644	C	T	Zfp574	Missense	NM_001168506	c.C2090T	p.P697L	0.476
A1	A1-3-2	chr8	94941666	94941666	C	T	Gpr114	Missense	NM_001145972	c.C1358T	p.A453V	0.118
A1	A1-3-2	chr8	27154308	27154308	G	A	Rab11fip1	Missense	NM_001080813	c.C1448T	p.P483L	0.485
A1	A1-3-2	chr9	14572677	14572677	C	T	Amotl1	Missense	NM_001081395	c.G1589A	p.R530Q	0.284
A1	A1-3-2	chr9	54335830	54335830	T	A	Gldn	Nonsense	NM_177350	c.T1095A	p.Y365X	0.5
A1	A1-3-2	chr9	44388846	44388846	C	T	Hyou1	Missense	NM_021395	c.C2431T	p.R811W	0.076
A1	A1-3-2	chr10	11295240	11295240	T	G	Fbxo30	Missense	NM_027968	c.T2061G	p.F687L	0.513
A1	A1-3-2	chr10	129912132	129912132	C	T	Olfr816	Missense	NM_146672	c.G145A	p.V49M	0.306
A1	A1-3-2	chr10	94794675	94794675	C	A	Plxnc1	Nonsense	NM_018797	c.G4456T	p.E1486X	0.461
A1	A1-3-2	chr10	51723856	51723856	C	T	Rfx6	Missense	NM_001159389	c.C1799T	p.P600L	0.543
A1	A1-3-2	chr10	26245602	26245602	T	C	Samd3	Missense	NM_001115154	c.T616C	p.F206L	0.356
A1	A1-3-2	chr10	33200204	33200204	C	A	Trdn	Missense	NM_029726	c.C829A	p.H277N	0.441
A1	A1-3-2	chr11	120273314	120273314	G	A	Bahcc1	Splice site	NM_198423	c.2228-1G>A	NA	0.508
A1	A1-3-2	chr11	106813834	106813834	G	A	Cep95	Missense	NM_177088	c.G1514A	p.R505K	0.558
A1	A1-3-2	chr11	54916905	54916905	G	A	Tnip1	Missense	NM_021327	c.C1709T	p.A570V	0.468
A1	A1-3-2	chr13	73268858	73268858	G	A	Irx4	Missense	NM_018885	c.G1372A	p.G458S	0.482
A1	A1-3-2	chr14	49774037	49774037	G	C	3632451O06Rik	Missense	NM_026142	c.C212G	p.T71S	0.203

A1	A1-3-2	chr14	37057443	37057443	A	T	Lrit1	Missense	NM_146245	c.A332T	p.E111V	0.394
A1	A1-3-2	chr14	79770248	79770248	C	T	Pcdh8	Missense	NM_021543	c.G874A	p.E292K	0.532
A1	A1-3-2	chr14	70440016	70440016	G	A	Polr3d	Missense	NM_001164082	c.C919T	p.R307W	0.138
A1	A1-3-2	chr14	57643200	57643200	G	A	Xpo4	Missense	NM_020506	c.C199T	p.L67F	0.538
A1	A1-3-2	chr14	55063007	55063007	C	T	Zfhx2	Missense	NM_001039198	c.G7286A	p.S2429N	0.169
A1	A1-3-2	chr15	76106727	76106727	C	T	Eppk1	Missense	NM_144848	c.G5953A	p.G1985S	0.47
A1	A1-3-2	chr15	52335160	52335160	G	T	Slc30a8	Missense	NM_172816	c.G1000T	p.A334S	0.458
A1	A1-3-2	chr16	16673840	16673840	A	C	Olfr19	Missense	NM_146335	c.T140G	p.I47S	0.084
A1	A1-3-2	chr17	65256583	65256583	C	T	Tmem232	Missense	NM_001008973	c.G1814A	p.R605Q	0.409
A1	A1-3-2	chr18	61849326	61849326	C	T	Ablim3	Missense	NM_001164491	c.G547A	p.V183I	0.107
A1	A1-3-2	chr18	34863349	34863349	G	T	Egr1	Missense	NM_007913	c.G1183T	p.A395S	0.511
A1	A1-3-2	chr19	29717713	29717713	G	A	9930021J03Rik	Missense	NM_172836	c.C4580T	p.P1527L	0.512
A1	A1-3-2	chr19	54047310	54047310	G	A	Adra2a	Missense	NM_007417	c.G1096A	p.E366K	0.47
A1	A1-3-2	chrX	94538510	94538510	T	C	Maged1	Splice site	NM_019791	c.1414-2A>G	NA	0.414
A1	A1-3-2	chrX	139824102	139824102	C	T	Morc4	Missense	NM_001193309	c.G2213A	p.G738E	0.556
A1	A1-3-5	chr1	110925165	110925165	G	A	Cdh19	Missense	NM_001081386	c.C1039T	p.H347Y	0.361
A1	A1-3-5	chr2	93696152	93696152	G	A	Ext2	Missense	NM_010163	c.C2138T	p.P713L	0.447
A1	A1-3-5	chr2	155421512	155421512	G	A	Ncoa6	Missense	NM_019825	c.C1001T	p.P334L	0.376
A1	A1-3-5	chr2	124664020	124664020	G	A	Sema6d	Splice site	NM_199241	c.1934-1G>A	NA	0.551
A1	A1-3-5	chr2	59838975	59838975	G	A	Tanc1	Missense	NM_198294	c.G3803A	p.G1268E	0.526
A1	A1-3-5	chr3	106149750	106149750	T	C	Chi3l3	Missense	NM_009892	c.A877G	p.K293E	0.28
A1	A1-3-5	chr4	63225586	63225586	T	G	Col27a1	Missense	NM_025685	c.T1510G	p.L504V	0.447
A1	A1-3-5	chr5	120609628	120609628	G	A	1110008J03Rik	Missense	NM_029096	c.C604T	p.P202S	0.481
A1	A1-3-5	chr5	35043862	35043880	GTGGGCGACCACTGGGCCC	-	Hgfac	Nonsense	NM_019447	c.628_646del	p.G211X	0.102
A1	A1-3-5	chr6	38196439	38196439	G	A	D630045J12Rik	Missense	NM_194061	c.C793T	p.L265F	0.449
A1	A1-3-5	chr6	60988306	60988306	A	C	Mmrn1	Missense	NM_027613	c.A3320C	p.N1107T	0.094
A1	A1-3-5	chr6	85337030	85337030	G	A	Rab11fip5	Missense	NM_001003955	c.C3950T	p.P1317L	0.5
A1	A1-3-5	chr7	128133275	128133275	T	C	Itgax	Missense	NM_021334	c.T572C	p.L191P	0.438
A1	A1-3-5	chr7	102752989	102752989	T	A	Olfr560	Missense	NM_147113	c.A939T	p.E313D	0.584
A1	A1-3-5	chr7	25081644	25081644	C	T	Zfp574	Missense	NM_001168506	c.C2090T	p.P697L	0.464
A1	A1-3-5	chr8	27154308	27154308	G	A	Rab11fip1	Missense	NM_001080813	c.C1448T	p.P483L	0.432
A1	A1-3-5	chr9	14572677	14572677	C	T	Amotl1	Missense	NM_001081395	c.G1589A	p.R530Q	0.318
A1	A1-3-5	chr9	54335830	54335830	T	A	Gldn	Nonsense	NM_177350	c.T1095A	p.Y365X	0.541
A1	A1-3-5	chr10	11295240	11295240	T	G	Fbxo30	Missense	NM_027968	c.T2061G	p.F687L	0.444
A1	A1-3-5	chr10	129912132	129912132	C	T	Olfr816	Missense	NM_146672	c.G145A	p.V49M	0.524
A1	A1-3-5	chr10	94794675	94794675	C	A	Plxnc1	Nonsense	NM_018797	c.G4456T	p.E1486X	0.481
A1	A1-3-5	chr10	51723856	51723856	C	T	Rfx6	Missense	NM_001159389	c.C1799T	p.P600L	0.508
A1	A1-3-5	chr10	26245602	26245602	T	C	Samd3	Missense	NM_001115154	c.T616C	p.F206L	0.673
A1	A1-3-5	chr10	33200204	33200204	C	A	Trdn	Missense	NM_029726	c.C829A	p.H277N	0.484
A1	A1-3-5	chr11	120273314	120273314	G	A	Bahcc1	Splice site	NM_198423	c.2228-1G>A	NA	0.448
A1	A1-3-5	chr11	106813834	106813834	G	A	Cep95	Missense	NM_177088	c.G1514A	p.R505K	0.387
A1	A1-3-5	chr11	54916905	54916905	G	A	Tnip1	Missense	NM_021327	c.C1709T	p.A570V	0.479
A1	A1-3-5	chr13	58382465	58382465	G	A	2210016F16Rik	Missense	NM_027335	c.C730T	p.L244F	0.138
A1	A1-3-5	chr13	73268858	73268858	G	A	Irx4	Missense	NM_018885	c.G1372A	p.G458S	0.432
A1	A1-3-5	chr14	123980462	123980462	A	G	Fgf14	Missense	NM_010201	c.T628C	p.S210P	0.119
A1	A1-3-5	chr14	37057443	37057443	A	T	Lrit1	Missense	NM_146245	c.A332T	p.E111V	0.45
A1	A1-3-5	chr14	79770248	79770248	C	T	Pcdh8	Missense	NM_021543	c.G874A	p.E292K	0.518
A1	A1-3-5	chr14	57643200	57643200	G	A	Xpo4	Missense	NM_020506	c.C199T	p.L67F	0.609
A1	A1-3-5	chr15	76106727	76106727	C	T	Eppk1	Missense	NM_144848	c.G5953A	p.G1985S	0.489
A1	A1-3-5	chr15	83119999	83119999	G	A	Rrp7a	Missense	NM_029101	c.C235T	p.L79F	0.147
A1	A1-3-5	chr15	101039945	101039945	G	A	Scn8a	Missense	NM_001077499	c.G5194A	p.G1732R	0.1
A1	A1-3-5	chr15	52335160	52335160	G	T	Slc30a8	Missense	NM_172816	c.G1000T	p.A334S	0.415
A1	A1-3-5	chr17	65256583	65256583	C	T	Tmem232	Missense	NM_001008973	c.G1814A	p.R605Q	0.529
A1	A1-3-5	chr18	34863349	34863349	G	T	Egr1	Missense	NM_007913	c.G1183T	p.A395S	0.493
A1	A1-3-5	chr19	29717713	29717713	G	A	9930021J03Rik	Missense	NM_172836	c.C4580T	p.P1527L	0.505
A1	A1-3-5	chr19	54047310	54047310	G	A	Adra2a	Missense	NM_007417	c.G1096A	p.E366K	0.471
A1	A1-3-5	chrX	94538510	94538510	T	C	Maged1	Splice site	NM_019791	c.1414-2A>G	NA	0.56
A1	A1-3-5	chrX	139824102	139824102	C	T	Morc4	Missense	NM_001193309	c.G2213A	p.G738E	0.534
A1	A1-3-5	chrX	96160175	96160175	C	T	Msn	Missense	NM_010833	c.C883T	p.R295C	0.2
A1	A1-3-5	chrX	37217843	37217843	C	T	Rhox1	Missense	NM_001025084	c.G388A	p.E130K	0.082
A1	A1-3-6	chr1	110925165	110925165	G	A	Cdh19	Missense	NM_001081386	c.C1039T	p.H347Y	0.489
A1	A1-3-6	chr2	93696152	93696152	G	A	Ext2	Missense	NM_010163	c.C2138T	p.P713L	0.333
A1	A1-3-6	chr2	155421512	155421512	G	A	Ncoa6	Missense	NM_019825	c.C1001T	p.P334L	0.464
A1	A1-3-6	chr2	124664020	124664020	G	A	Sema6d	Splice site	NM_199241	c.1934-1G>A	NA	0.619
A1	A1-3-6	chr2	59838975	59838975	G	A	Tanc1	Missense	NM_198294	c.G3803A	p.G1268E	0.372
A1	A1-3-6	chr3	106149750	106149750	T	C	Chi3l3	Missense	NM_009892	c.A877G	p.K293E	0.263
A1	A1-3-6	chr3	5401735	5401735	A	G	Zfhx4	Missense	NM_030708	c.A6952G	p.S2318G	0.162
A1	A1-3-6	chr4	63225586	63225586	T	G	Col27a1	Missense	NM_025685	c.T1510G	p.L504V	0.451
A1	A1-3-6	chr5	120609628	120609628	G	A	1110008J03Rik	Missense	NM_029096	c.C604T	p.P202S	0.487
A1	A1-3-6	chr5	35043862	35043880	GTGGGCGACCACTGGGCCC	-	Hgfac	Nonsense	NM_019447	c.628_646del	p.G211X	0.099
A1	A1-3-6	chr5	14539711	14539711	C	T	Pclo	Missense	NM_011995	c.C2024T	p.A675V	0.074
A1	A1-3-6	chr5	121168003	121168003	C	T	Ptpn11	Missense	NM_011202	c.G178A	p.G60R	0.18
A1	A1-3-6	chr6	38196439	38196439	G	A	D630045J12Rik	Missense	NM_194061	c.C793T	p.L265F	0.507
A1	A1-3-6	chr6	85337030	85337030	G	A	Rab11fip5	Missense	NM_001003955	c.C3950T	p.P1317L	0.517
A1	A1-3-6	chr7	128133275	128133275	T	C	Itgax	Missense	NM_021334	c.T572C	p.L191P	0.467
A1	A1-3-6	chr7	102752989	102752989	T	A	Olfr560	Missense	NM_147113	c.A939T	p.E313D	0.529
A1	A1-3-6	chr7	25081644	25081644	C	T	Zfp574	Missense	NM_001168506	c.C2090T	p.P697L	0.474
A1	A1-3-6	chr8	27154308	27154308	G	A	Rab11fip1	Missense	NM_001080813	c.C1448T	p.P483L	0.438
A1	A1-3-6	chr9	14572677	14572677	C	T	Amotl1	Missense	NM_001081395	c.G1589A	p.R530Q	0.289
A1	A1-3-6	chr9	15959858	15959858	G	T	Fat3	Missense	NM_001080814	c.C11236A	p.Q3746K	0.137
A1	A1-3-6	chr9	54335830	54335830	T	A	Gldn	Nonsense	NM_177350	c.T1095A	p.Y365X	0.485
A1	A1-3-6	chr10	11295240	11295240	T	G	Fbxo30	Missense	NM_027968	c.T2061G	p.F687L	0.483
A1	A1-3-6	chr10	129912132	129912132	C	T	Olfr816	Missense	NM_146672	c.G145A	p.V49M	0.471
A1	A1-3-6	chr10	94794675	94794675	C	A	Plxnc1	Nonsense	NM_018797	c.G4456T	p.E1486X	0.515
A1	A1-3-6	chr10	51723856	51723856	C	T	Rfx6	Missense	NM_001159389	c.C1799T	p.P600L	0.528
A1	A1-3-6	chr10	26245602	26245602	T	C	Samd3	Missense	NM_001115154	c.T616C	p.F206L	0.382
A1	A1-3-6	chr10	33200204	33200204	C	A	Trdn	Missense	NM_029726	c.C829A	p.H277N	0.478
A1	A1-3-6	chr11	120273314	120273314	G	A	Bahcc1	Splice site	NM_198423	c.2228-1G>A	NA	0.531
A1	A1-3-6	chr11	106813834	106813834	G	A	Cep95	Missense	NM_177088	c.G1514A	p.R505K	0.571
A1	A1-3-6	chr11	54916905	54916905	G	A	Tnip1	Missense	NM_021327	c.C1709T	p.A570V	0.476
A1	A1-3-6	chr13	73268858	73268858	G	A	Irx4	Missense	NM_018885	c.G1372A	p.G458S	0.523
A1	A1-3-6	chr14	37057443	37057443	A	T	Lrit1	Missense	NM_146245	c.A332T	p.E111V	0.546
A1	A1-3-6	chr14	79770248	79770248	C	T	Pcdh8	Missense	NM_021543	c.G874A	p.E292K	0.543
A1	A1-3-6	chr14	57643200	57643200	G	A	Xpo4	Missense	NM_020506	c.C199T	p.L67F	0.525
A1	A1-3-6	chr15	76106727	76106727	C	T	Eppk1	Missense	NM_144848	c.G5953A	p.G1985S	0.445
A1	A1-3-6	chr15	52335160	52335160	G	T	Slc30a8	Missense	NM_172816	c.G1000T	p.A334S	0.523
A1	A1-3-6	chr17	65256583	65256583	C	T	Tmem232	Missense	NM_001008973	c.G1814A	p.R605Q	0.591
A1	A1-3-6	chr18	34863349	34863349	G	T	Egr1	Missense	NM_007913	c.G1183T	p.A395S	0.5
A1	A1-3-6	chr19	29717713	29717713	G	A	9930021J03Rik	Missense	NM_172836	c.C4580T	p.P1527L	0.512
A1	A1-3-6	chr19	54047310	54047310	G	A	Adra2a	Missense	NM_007417	c.G1096A	p.E366K	0.498
A1	A1-3-6	chrX	94538510	94538510	T	C	Maged1	Splice site	NM_019791	c.1414-2A>G	NA	0.422
A1	A1-3-6	chrX	139824102	139824102	C	T	Morc4	Missense	NM_001193309	c.G2213A	p.G738E	0.529
A1	A1-3-6	chrX	96160175	96160175	C	T	Msn	Missense	NM_010833	c.C883T	p.R295C	0.209
A1	A1-4-1	chr1	154472253	154472253	G	A	Cacna1e	Nonsense	NM_009782	c.C2761T	p.R921X	0.137
A1	A1-4-1	chr1	110925165	110925165	G	A	Cdh19	Missense	NM_001081386	c.C1039T	p.H347Y	0.406
A1	A1-4-1	chr1	88215102	88215102	G	A	Ugt1a1	Missense	NM_201645	c.G929A	p.G310E	0.08
A1	A1-4-1	chr2	93696152	93696152	G	A	Ext2	Missense	NM_010163	c.C2138T	p.P713L	0.432
A1	A1-4-1	chr2	151970755	151970755	G	A	Fam110a	Missense	NM_028666	c.C94T	p.P32S	0.111
A1	A1-4-1	chr2	121573617	121573617	T	C	Frmd5	Missense	NM_172673	c.A479G	p.K160R	0.116
A1	A1-4-1	chr2	74692005	74692005	C	T	Hoxd10	Missense	NM_013554	c.C26T	p.A9V	0.121
A1	A1-4-1	chr2	40596939	40596939	C	T	Lrp1b	Missense	NM_053011	c.G13795A	p.A4599T	0.083
A1	A1-4-1	chr2	155421512	155421512	G	A	Ncoa6	Missense	NM_019825	c.C1001T	p.P334L	0.475
A1	A1-4-1	chr2	124664020	124664020	G	A	Sema6d	Splice site	NM_199241	c.1934-1G>A	NA	0.429
A1	A1-4-1	chr2	59838975	59838975	G	A	Tanc1	Missense	NM_198294	c.G3803A	p.G1268E	0.643
A1	A1-4-1	chr3	106149750	106149750	T	C	Chi3l3	Missense	NM_009892	c.A877G	p.K293E	0.216
A1	A1-4-1	chr4	63225586	63225586	T	G	Col27a1	Missense	NM_025685	c.T1510G	p.L504V	0.473
A1	A1-4-1	chr5	120609628	120609628	G	A	1110008J03Rik	Missense	NM_029096	c.C604T	p.P202S	0.483
A1	A1-4-1	chr6	38196439	38196439	G	A	D630045J12Rik	Missense	NM_194061	c.C793T	p.L265F	0.431
A1	A1-4-1	chr6	85337030	85337030	G	A	Rab11fip5	Missense	NM_001003955	c.C3950T	p.P1317L	0.538
A1	A1-4-1	chr7	128133275	128133275	T	C	Itgax	Missense	NM_021334	c.T572C	p.L191P	0.495
A1	A1-4-1	chr7	102752989	102752989	T	A	Olfr560	Missense	NM_147113	c.A939T	p.E313D	0.471
A1	A1-4-1	chr7	64240615	64240615	G	A	Trpm1	Missense	NM_001039104	c.G2965A	p.D989N	0.092
A1	A1-4-1	chr7	25081644	25081644	C	T	Zfp574	Missense	NM_001168506	c.C2090T	p.P697L	0.48
A1	A1-4-1	chr8	94941666	94941666	C	T	Gpr114	Missense	NM_001145972	c.C1358T	p.A453V	0.071

A1	A1-4-1	chr8	27154308	27154308	G	A	Rab11fip1	Missense	NM_001080813	c.C1448T	p.P483L	0.356
A1	A1-4-1	chr9	14572677	14572677	C	T	Amotl1	Missense	NM_001081395	c.G1589A	p.R530Q	0.427
A1	A1-4-1	chr9	62432454	62432454	G	A	Coro2b	Missense	NM_175484	c.C386T	p.A129V	0.198
A1	A1-4-1	chr9	54335830	54335830	T	A	Gldn	Nonsense	NM_177350	c.T1095A	p.Y365X	0.495
A1	A1-4-1	chr10	11295240	11295240	T	G	Fbxo30	Missense	NM_027968	c.T2061G	p.F687L	0.678
A1	A1-4-1	chr10	129912132	129912132	C	T	Olfr816	Missense	NM_146672	c.G145A	p.V49M	0.531
A1	A1-4-1	chr10	94794675	94794675	C	A	Plxnc1	Nonsense	NM_018797	c.G4456T	p.E1486X	0.578
A1	A1-4-1	chr10	51723856	51723856	C	T	Rfx6	Missense	NM_001159389	c.C1799T	p.P600L	0.566
A1	A1-4-1	chr10	26245602	26245602	T	C	Samd3	Missense	NM_001115154	c.T616C	p.F206L	0.411
A1	A1-4-1	chr10	33200204	33200204	C	A	Trdn	Missense	NM_029726	c.C829A	p.H277N	0.584
A1	A1-4-1	chr11	120273314	120273314	G	A	Bahcc1	Splice site	NM_198423	c.2228-1G>A	NA	0.54
A1	A1-4-1	chr11	106813834	106813834	G	A	Cep95	Missense	NM_177088	c.G1514A	p.R505K	0.571
A1	A1-4-1	chr11	73847215	73847216	TC	AT	Olfr393	Missense	NM_147008	c.GA908_909AT	p.R303N	0.21
A1	A1-4-1	chr11	54916905	54916905	G	A	Tnip1	Missense	NM_021327	c.C1709T	p.A570V	0.47
A1	A1-4-1	chr13	73268858	73268858	G	A	Irx4	Missense	NM_018885	c.G1372A	p.G458S	0.566
A1	A1-4-1	chr14	37057443	37057443	A	T	Lrit1	Missense	NM_146245	c.A332T	p.E111V	0.446
A1	A1-4-1	chr14	79770248	79770248	C	T	Pcdh8	Missense	NM_021543	c.G874A	p.E292K	0.534
A1	A1-4-1	chr14	70440016	70440016	G	A	Polr3d	Missense	NM_001164082	c.C919T	p.R307W	0.115
A1	A1-4-1	chr14	57643200	57643200	G	A	Xpo4	Missense	NM_020506	c.C199T	p.L67F	0.645
A1	A1-4-1	chr14	55063007	55063007	C	T	Zfhx2	Missense	NM_001039198	c.G7286A	p.S2429N	0.156
A1	A1-4-1	chr15	76106727	76106727	C	T	Eppk1	Missense	NM_144848	c.G5953A	p.G1985S	0.457
A1	A1-4-1	chr15	52335160	52335160	G	T	Slc30a8	Missense	NM_172816	c.G1000T	p.A334S	0.368
A1	A1-4-1	chr17	65256583	65256583	C	T	Tmem232	Missense	NM_001008973	c.G1814A	p.R605Q	0.475
A1	A1-4-1	chr18	61849326	61849326	C	T	Ablim3	Missense	NM_001164491	c.G547A	p.V183I	0.105
A1	A1-4-1	chr18	34863349	34863349	G	T	Egr1	Missense	NM_007913	c.G1183T	p.A395S	0.468
A1	A1-4-1	chr19	29717713	29717713	G	A	9930021J03Rik	Missense	NM_172836	c.C4580T	p.P1527L	0.518
A1	A1-4-1	chr19	54047310	54047310	G	A	Adra2a	Missense	NM_007417	c.G1096A	p.E366K	0.493
A1	A1-4-1	chrX	94538510	94538510	T	C	Maged1	Splice site	NM_019791	c.1414-2A>G	NA	0.42
A1	A1-4-1	chrX	139824102	139824102	C	T	Morc4	Missense	NM_001193309	c.G2213A	p.G738E	0.46
A1	A1-4-3	chr1	154472253	154472253	G	A	Cacna1e	Nonsense	NM_009782	c.C2761T	p.R921X	0.144
A1	A1-4-3	chr1	110925165	110925165	G	A	Cdh19	Missense	NM_001081386	c.C1039T	p.H347Y	0.579
A1	A1-4-3	chr1	88215102	88215102	G	A	Ugt1a1	Missense	NM_201645	c.G929A	p.G310E	0.074
A1	A1-4-3	chr2	93696152	93696152	G	A	Ext2	Missense	NM_010163	c.C2138T	p.P713L	0.443
A1	A1-4-3	chr2	151970755	151970755	G	A	Fam110a	Missense	NM_028666	c.C94T	p.P32S	0.282
A1	A1-4-3	chr2	40596939	40596939	C	T	Lrp1b	Missense	NM_053011	c.G13795A	p.A4599T	0.102
A1	A1-4-3	chr2	155421512	155421512	G	A	Ncoa6	Missense	NM_019825	c.C1001T	p.P334L	0.456
A1	A1-4-3	chr2	38740483	38740483	G	A	Nr6a1	Missense	NM_001159548	c.C595T	p.P199S	0.123
A1	A1-4-3	chr2	59838975	59838975	G	A	Tanc1	Missense	NM_198294	c.G3803A	p.G1268E	0.541
A1	A1-4-3	chr3	106149750	106149750	T	C	Chi3l3	Missense	NM_009892	c.A877G	p.K293E	0.281
A1	A1-4-3	chr4	63225586	63225586	T	G	Col27a1	Missense	NM_025685	c.T1510G	p.L504V	0.494
A1	A1-4-3	chr4	56790899	56790899	C	A	Ikbkap	Missense	NM_026079	c.G853T	p.D285Y	0.255
A1	A1-4-3	chr5	120609628	120609628	G	A	1110008J03Rik	Missense	NM_029096	c.C604T	p.P202S	0.469
A1	A1-4-3	chr6	38196439	38196439	G	A	D630045J12Rik	Missense	NM_194061	c.C793T	p.L265F	0.434
A1	A1-4-3	chr6	85337030	85337030	G	A	Rab11fip5	Missense	NM_001003955	c.C3950T	p.P1317L	0.609
A1	A1-4-3	chr6	83151912	83151912	G	A	Rtkn	Missense	NM_001136227	c.G1387A	p.A463T	0.147
A1	A1-4-3	chr7	128133275	128133275	T	C	Itgax	Missense	NM_021334	c.T572C	p.L191P	0.49
A1	A1-4-3	chr7	102752989	102752989	T	A	Olfr560	Missense	NM_147113	c.A939T	p.E313D	0.597
A1	A1-4-3	chr7	25081644	25081644	C	T	Zfp574	Missense	NM_001168506	c.C2090T	p.P697L	0.495
A1	A1-4-3	chr8	94941666	94941666	C	T	Gpr114	Missense	NM_001145972	c.C1358T	p.A453V	0.118
A1	A1-4-3	chr8	27154308	27154308	G	A	Rab11fip1	Missense	NM_001080813	c.C1448T	p.P483L	0.494
A1	A1-4-3	chr9	14572677	14572677	C	T	Amotl1	Missense	NM_001081395	c.G1589A	p.R530Q	0.333
A1	A1-4-3	chr9	54335830	54335830	T	A	Gldn	Nonsense	NM_177350	c.T1095A	p.Y365X	0.391
A1	A1-4-3	chr9	44388846	44388846	C	T	Hyou1	Missense	NM_021395	c.C2431T	p.R811W	0.094
A1	A1-4-3	chr10	11295240	11295240	T	G	Fbxo30	Missense	NM_027968	c.T2061G	p.F687L	0.66
A1	A1-4-3	chr10	79755182	79755182	G	A	Fgf22	Missense	NM_023304	c.G64A	p.G22R	0.137
A1	A1-4-3	chr10	129912132	129912132	C	T	Olfr816	Missense	NM_146672	c.G145A	p.V49M	0.375
A1	A1-4-3	chr10	94794675	94794675	C	A	Plxnc1	Nonsense	NM_018797	c.G4456T	p.E1486X	0.463
A1	A1-4-3	chr10	51723856	51723856	C	T	Rfx6	Missense	NM_001159389	c.C1799T	p.P600L	0.422
A1	A1-4-3	chr10	26245602	26245602	T	C	Samd3	Missense	NM_001115154	c.T616C	p.F206L	0.476
A1	A1-4-3	chr10	33200204	33200204	C	A	Trdn	Missense	NM_029726	c.C829A	p.H277N	0.444
A1	A1-4-3	chr11	120273314	120273314	G	A	Bahcc1	Splice site	NM_198423	c.2228-1G>A	NA	0.533
A1	A1-4-3	chr11	106813834	106813834	G	A	Cep95	Missense	NM_177088	c.G1514A	p.R505K	0.476
A1	A1-4-3	chr11	54916905	54916905	G	A	Tnip1	Missense	NM_021327	c.C1709T	p.A570V	0.475
A1	A1-4-3	chr12	104864940	104864940	C	G	Clmn	Missense	NM_053155	c.G12C	p.Q4H	0.114
A1	A1-4-3	chr13	73268858	73268858	G	A	Irx4	Missense	NM_018885	c.G1372A	p.G458S	0.464
A1	A1-4-3	chr14	37057443	37057443	A	T	Lrit1	Missense	NM_146245	c.A332T	p.E111V	0.5
A1	A1-4-3	chr14	79770248	79770248	C	T	Pcdh8	Missense	NM_021543	c.G874A	p.E292K	0.515
A1	A1-4-3	chr14	57643200	57643200	G	A	Xpo4	Missense	NM_020506	c.C199T	p.L67F	0.591
A1	A1-4-3	chr14	55063007	55063007	C	T	Zfhx2	Missense	NM_001039198	c.G7286A	p.S2429N	0.116
A1	A1-4-3	chr15	76106727	76106727	C	T	Eppk1	Missense	NM_144848	c.G5953A	p.G1985S	0.418
A1	A1-4-3	chr15	52335160	52335160	G	T	Slc30a8	Missense	NM_172816	c.G1000T	p.A334S	0.489
A1	A1-4-3	chr17	65256583	65256583	C	T	Tmem232	Missense	NM_001008973	c.G1814A	p.R605Q	0.375
A1	A1-4-3	chr18	61849326	61849326	C	T	Ablim3	Missense	NM_001164491	c.G547A	p.V183I	0.137
A1	A1-4-3	chr18	34863349	34863349	G	T	Egr1	Missense	NM_007913	c.G1183T	p.A395S	0.497
A1	A1-4-3	chr19	29717713	29717713	G	A	9930021J03Rik	Missense	NM_172836	c.C4580T	p.P1527L	0.464
A1	A1-4-3	chr19	54047310	54047310	G	A	Adra2a	Missense	NM_007417	c.G1096A	p.E366K	0.425
A1	A1-4-3	chrX	94538510	94538510	T	C	Maged1	Splice site	NM_019791	c.1414-2A>G	NA	0.458
A1	A1-4-3	chrX	139824102	139824102	C	T	Morc4	Missense	NM_001193309	c.G2213A	p.G738E	0.556
A1	A1-4-5	chr1	110925165	110925165	G	A	Cdh19	Missense	NM_001081386	c.C1039T	p.H347Y	0.471
A1	A1-4-5	chr2	154202621	154202621	G	A	Bpifb1	Missense	NM_001012392	c.G7A	p.G3S	0.391
A1	A1-4-5	chr2	93696152	93696152	G	A	Ext2	Missense	NM_010163	c.C2138T	p.P713L	0.445
A1	A1-4-5	chr2	155421512	155421512	G	A	Ncoa6	Missense	NM_019825	c.C1001T	p.P334L	0.459
A1	A1-4-5	chr2	87418099	87418099	C	T	Olfr1123	Missense	NM_146350	c.C50T	p.S17L	0.34
A1	A1-4-5	chr2	124664020	124664020	G	A	Sema6d	Splice site	NM_199241	c.1934-1G>A	NA	0.465
A1	A1-4-5	chr2	59838975	59838975	G	A	Tanc1	Missense	NM_198294	c.G3803A	p.G1268E	0.333
A1	A1-4-5	chr3	106149750	106149750	T	C	Chi3l3	Missense	NM_009892	c.A877G	p.K293E	0.232
A1	A1-4-5	chr4	63225586	63225586	T	G	Col27a1	Missense	NM_025685	c.T1510G	p.L504V	0.39
A1	A1-4-5	chr5	120609628	120609628	G	A	1110008J03Rik	Missense	NM_029096	c.C604T	p.P202S	0.509
A1	A1-4-5	chr5	114064898	114064898	C	T	Svop	Nonsense	NM_026805	c.G252A	p.W84X	0.083
A1	A1-4-5	chr5	137403232	137403232	G	C	Zan	Missense	NM_011741	c.C12224G	p.A4075G	0.126
A1	A1-4-5	chr6	38196439	38196439	G	A	D630045J12Rik	Missense	NM_194061	c.C793T	p.L265F	0.411
A1	A1-4-5	chr6	85337030	85337030	G	A	Rab11fip5	Missense	NM_001003955	c.C3950T	p.P1317L	0.514
A1	A1-4-5	chr7	119575707	119575707	G	A	Acsm2	Missense	NM_001177978	c.G694A	p.E232K	0.204
A1	A1-4-5	chr7	128133275	128133275	T	C	Itgax	Missense	NM_021334	c.T572C	p.L191P	0.408
A1	A1-4-5	chr7	102752989	102752989	T	A	Olfr560	Missense	NM_147113	c.A939T	p.E313D	0.43
A1	A1-4-5	chr7	25081644	25081644	C	T	Zfp574	Missense	NM_001168506	c.C2090T	p.P697L	0.498
A1	A1-4-5	chr8	27154308	27154308	G	A	Rab11fip1	Missense	NM_001080813	c.C1448T	p.P483L	0.425
A1	A1-4-5	chr9	14572677	14572677	C	T	Amotl1	Missense	NM_001081395	c.G1589A	p.R530Q	0.344
A1	A1-4-5	chr9	54335830	54335830	T	A	Gldn	Nonsense	NM_177350	c.T1095A	p.Y365X	0.388
A1	A1-4-5	chr10	11295240	11295240	T	G	Fbxo30	Missense	NM_027968	c.T2061G	p.F687L	0.542
A1	A1-4-5	chr10	129912132	129912132	C	T	Olfr816	Missense	NM_146672	c.G145A	p.V49M	0.368
A1	A1-4-5	chr10	94794675	94794675	C	A	Plxnc1	Nonsense	NM_018797	c.G4456T	p.E1486X	0.353
A1	A1-4-5	chr10	51723856	51723856	C	T	Rfx6	Missense	NM_001159389	c.C1799T	p.P600L	0.487
A1	A1-4-5	chr10	26245602	26245602	T	C	Samd3	Missense	NM_001115154	c.T616C	p.F206L	0.388
A1	A1-4-5	chr10	33200204	33200204	C	A	Trdn	Missense	NM_029726	c.C829A	p.H277N	0.567
A1	A1-4-5	chr10	79148865	79148865	T	C	Vmn2r80	Missense	NM_001103368	c.T50C	p.F17S	0.165
A1	A1-4-5	chr11	120273314	120273314	G	A	Bahcc1	Splice site	NM_198423	c.2228-1G>A	NA	0.457
A1	A1-4-5	chr11	106813834	106813834	G	A	Cep95	Missense	NM_177088	c.G1514A	p.R505K	0.468
A1	A1-4-5	chr11	50853217	50853217	G	A	Grm6	Missense	NM_173372	c.G514A	p.A172T	0.247
A1	A1-4-5	chr11	54916905	54916905	G	A	Tnip1	Missense	NM_021327	c.C1709T	p.A570V	0.502
A1	A1-4-5	chr13	58382465	58382465	G	A	2210016F16Rik	Missense	NM_027335	c.C730T	p.L244F	0.266
A1	A1-4-5	chr13	73268858	73268858	G	A	Irx4	Missense	NM_018885	c.G1372A	p.G458S	0.489
A1	A1-4-5	chr14	123980462	123980462	A	G	Fgf14	Missense	NM_010201	c.T628C	p.S210P	0.41
A1	A1-4-5	chr14	37057443	37057443	A	T	Lrit1	Missense	NM_146245	c.A332T	p.E111V	0.52
A1	A1-4-5	chr14	79770248	79770248	C	T	Pcdh8	Missense	NM_021543	c.G874A	p.E292K	0.559
A1	A1-4-5	chr14	57643200	57643200	G	A	Xpo4	Missense	NM_020506	c.C199T	p.L67F	0.649
A1	A1-4-5	chr15	76106727	76106727	C	T	Eppk1	Missense	NM_144848	c.G5953A	p.G1985S	0.413
A1	A1-4-5	chr15	83119999	83119999	G	A	Rrp7a	Missense	NM_029101	c.C235T	p.L79F	0.308
A1	A1-4-5	chr15	101039945	101039945	G	A	Scn8a	Missense	NM_001077499	c.G5194A	p.G1732R	0.266
A1	A1-4-5	chr15	52335160	52335160	G	T	Slc30a8	Missense	NM_172816	c.G1000T	p.A334S	0.448
A1	A1-4-5	chr16	88506266	88506266	C	T	Cldn17	Missense	NM_181490	c.G574A	p.E192K	0.309
A1	A1-4-5	chr17	40880058	40880058	A	T	9130008F23Rik	Missense	NM_027834	c.T479A	p.M160K	0.29
A1	A1-4-5	chr17	65256583	65256583	C	T	Tmem232	Missense	NM_001008973	c.G1814A	p.R605Q	0.538
A1	A1-4-5	chr18	34863349	34863349	G	T	Egr1	Missense	NM_007913	c.G1183T	p.A395S	0.509

A1	A1-4-5	chr19	29717713	29717713	G	A	9930021J03Rik	Missense	NM_172836	c.C4580T	p.P1527L	0.427
A1	A1-4-5	chr19	54047310	54047310	G	A	Adra2a	Missense	NM_007417	c.G1096A	p.E366K	0.477
A1	A1-4-5	chrX	94538510	94538510	T	C	Maged1	Splice site	NM_019791	c.1414-2A>G	NA	0.432
A1	A1-4-5	chrX	139824102	139824102	C	T	Morc4	Missense	NM_001193309	c.G2213A	p.G738E	0.673
A1	A1-4-5	chrX	96160175	96160175	C	T	Msn	Missense	NM_010833	c.C883T	p.R295C	0.07
A1	A1-4-5	chrX	37217843	37217843	C	T	Rhox1	Missense	NM_001025084	c.G388A	p.E130K	0.294
A1	A1-4-5	chrX	42211151	42211151	G	T	Stag2	Missense	NM_001077712	c.G197T	p.G66V	0.3
A1	A1-4-7	chr1	110925165	110925165	G	A	Cdh19	Missense	NM_001081386	c.C1039T	p.H347Y	0.559
A1	A1-4-7	chr2	93696152	93696152	G	A	Ext2	Missense	NM_010163	c.C2138T	p.P713L	0.384
A1	A1-4-7	chr2	155421512	155421512	G	A	Ncoa6	Missense	NM_019825	c.C1001T	p.P334L	0.5
A1	A1-4-7	chr2	101641954	101641954	C	T	Rag1	Missense	NM_009019	c.G2842A	p.E948K	0.169
A1	A1-4-7	chr2	124664020	124664020	G	A	Sema6d	Splice site	NM_199241	c.1934-1G>A	NA	0.643
A1	A1-4-7	chr2	59838975	59838975	G	A	Tanc1	Missense	NM_198294	c.G3803A	p.G1268E	0.455
A1	A1-4-7	chr3	106149750	106149750	T	C	Chi3l3	Missense	NM_009892	c.A877G	p.K293E	0.252
A1	A1-4-7	chr3	5401735	5401735	A	G	Zfhx4	Missense	NM_030708	c.A6952G	p.S2318G	0.411
A1	A1-4-7	chr4	63225586	63225586	T	G	Col27a1	Missense	NM_025685	c.T1510G	p.L504V	0.519
A1	A1-4-7	chr4	28947638	28947638	C	T	Epha7	Missense	NM_010141	c.C1909T	p.R637C	0.276
A1	A1-4-7	chr5	120609628	120609628	G	A	1110008J03Rik	Missense	NM_029096	c.C604T	p.P202S	0.512
A1	A1-4-7	chr5	35042599	35042599	C	T	Hgfac	Missense	NM_019447	c.C323T	p.P108L	0.133
A1	A1-4-7	chr5	121168003	121168003	C	T	Ptpn11	Missense	NM_011202	c.G178A	p.G60R	0.417
A1	A1-4-7	chr6	38196439	38196439	G	A	D630045J12Rik	Missense	NM_194061	c.C793T	p.L265F	0.527
A1	A1-4-7	chr6	85337030	85337030	G	A	Rab11fip5	Missense	NM_001003955	c.C3950T	p.P1317L	0.397
A1	A1-4-7	chr7	82574004	82574004	G	A	Adamtsl3	Missense	NM_001190374	c.G2992A	p.G998S	0.129
A1	A1-4-7	chr7	128133275	128133275	T	C	Itgax	Missense	NM_021334	c.T572C	p.L191P	0.566
A1	A1-4-7	chr7	102752989	102752989	T	A	Olfr560	Missense	NM_147113	c.A939T	p.E313D	0.612
A1	A1-4-7	chr7	25081644	25081644	C	T	Zfp574	Missense	NM_001168506	c.C2090T	p.P697L	0.464
A1	A1-4-7	chr8	27154308	27154308	G	A	Rab11fip1	Missense	NM_001080813	c.C1448T	p.P483L	0.439
A1	A1-4-7	chr9	14572677	14572677	C	T	Amotl1	Missense	NM_001081395	c.G1589A	p.R530Q	0.292
A1	A1-4-7	chr9	15959858	15959858	G	T	Fat3	Missense	NM_001080814	c.C11236A	p.Q3746K	0.469
A1	A1-4-7	chr9	54335830	54335830	T	A	Gldn	Nonsense	NM_177350	c.T1095A	p.Y365X	0.595
A1	A1-4-7	chr10	11295240	11295240	T	G	Fbxo30	Missense	NM_027968	c.T2061G	p.F687L	0.419
A1	A1-4-7	chr10	129912132	129912132	C	T	Olfr816	Missense	NM_146672	c.G145A	p.V49M	0.455
A1	A1-4-7	chr10	94794675	94794675	C	A	Plxnc1	Nonsense	NM_018797	c.G4456T	p.E1486X	0.495
A1	A1-4-7	chr10	51723856	51723856	C	T	Rfx6	Missense	NM_001159389	c.C1799T	p.P600L	0.447
A1	A1-4-7	chr10	26245602	26245602	T	C	Samd3	Missense	NM_001115154	c.T616C	p.F206L	0.667
A1	A1-4-7	chr10	33200204	33200204	C	A	Trdn	Missense	NM_029726	c.C829A	p.H277N	0.491
A1	A1-4-7	chr11	120273314	120273314	G	A	Bahcc1	Splice site	NM_198423	c.2228-1G>A	NA	0.556
A1	A1-4-7	chr11	106813834	106813834	G	A	Cep95	Missense	NM_177088	c.G1514A	p.R505K	0.667
A1	A1-4-7	chr11	54916905	54916905	G	A	Tnip1	Missense	NM_021327	c.C1709T	p.A570V	0.489
A1	A1-4-7	chr11	51070644	51070644	C	T	Zfp354a	Missense	NM_009329	c.C1678T	p.L560F	0.091
A1	A1-4-7	chr13	73268858	73268858	G	A	Irx4	Missense	NM_018885	c.G1372A	p.G458S	0.453
A1	A1-4-7	chr14	37057443	37057443	A	T	Lrit1	Missense	NM_146245	c.A332T	p.E111V	0.525
A1	A1-4-7	chr14	79770248	79770248	C	T	Pcdh8	Missense	NM_021543	c.G874A	p.E292K	0.522
A1	A1-4-7	chr14	57643200	57643200	G	A	Xpo4	Missense	NM_020506	c.C199T	p.L67F	0.407
A1	A1-4-7	chr15	76106727	76106727	C	T	Eppk1	Missense	NM_144848	c.G5953A	p.G1985S	0.508
A1	A1-4-7	chr15	52335160	52335160	G	T	Slc30a8	Missense	NM_172816	c.G1000T	p.A334S	0.519
A1	A1-4-7	chr16	33196120	33196120	C	T	Osbpl11	Missense	NM_176840	c.C244T	p.L82F	0.196
A1	A1-4-7	chr17	65256583	65256583	C	T	Tmem232	Missense	NM_001008973	c.G1814A	p.R605Q	0.448
A1	A1-4-7	chr18	34863349	34863349	G	T	Egr1	Missense	NM_007913	c.G1183T	p.A395S	0.509
A1	A1-4-7	chr19	29717713	29717713	G	A	9930021J03Rik	Missense	NM_172836	c.C4580T	p.P1527L	0.424
A1	A1-4-7	chr19	54047310	54047310	G	A	Adra2a	Missense	NM_007417	c.G1096A	p.E366K	0.494
A1	A1-4-7	chrX	134806689	134806690	AG	GA	Armcx2	Missense	NM_001166397	c.CT193_194TC	p.L65S	0.175
A1	A1-4-7	chrX	94538510	94538510	T	C	Maged1	Splice site	NM_019791	c.1414-2A>G	NA	0.425
A1	A1-4-7	chrX	139824102	139824102	C	T	Morc4	Missense	NM_001193309	c.G2213A	p.G738E	0.462
A1	A1-4-7	chrX	89405846	89405846	C	T	Pet2	Missense	NM_008821	c.G676A	p.V226I	0.081
A2	A2-1-1	chr2	35354896	35354896	T	A	4930568D16Rik	Missense	NM_029463	c.A443T	p.K148I	0.589
A2	A2-1-1	chr2	88411871	88411871	T	A	Olfr1180	Missense	NM_146918	c.A786T	p.E262D	0.44
A2	A2-1-1	chr2	153243656	153243656	C	T	Pofut1	Missense	NM_080463	c.C158T	p.A53V	0.531
A2	A2-1-1	chr3	131293658	131293658	C	T	Cyp2u1	Missense	NM_027816	c.G1273A	p.G425S	0.4
A2	A2-1-1	chr4	135750763	135750763	G	A	Il22ra1	Missense	NM_178257	c.G1144A	p.G382R	0.5
A2	A2-1-1	chr6	23115156	23115156	C	T	Aass	Splice site	NM_013930	c.473-1G>A	NA	0.571
A2	A2-1-1	chr6	83652425	83652425	C	T	Clec4f	Missense	NM_016751	c.G1150A	p.E384K	0.538
A2	A2-1-1	chr6	140593564	140593577	TGTCCCCGTCTCCT	-	Plekha5	Frameshift deletion	NM_144920	c.3725_3738del	p.L1242fs	0.118
A2	A2-1-1	chr6	47820441	47820441	T	C	Zfp786	Missense	NM_177882	c.A1562G	p.E521G	0.436
A2	A2-1-1	chr7	119595998	119595998	G	A	Acsm2	Missense	NM_001177978	c.G1732A	p.G578R	0.5
A2	A2-1-1	chr7	130503754	130503754	G	A	Ate1	Nonsense	NM_001136054	c.C904T	p.Q302X	0.31
A2	A2-1-1	chr7	81974048	81974048	G	A	Bnc1	Missense	NM_007562	c.C1430T	p.P477L	0.42
A2	A2-1-1	chr9	78414233	78414233	G	A	Ddx43	Missense	NM_001191044	c.G1183A	p.E395K	0.464
A2	A2-1-1	chr9	44263885	44263885	G	A	Nlrx1	Missense	NM_001163742	c.C593T	p.S198F	0.508
A2	A2-1-1	chr10	56162043	56162043	A	C	D630037F22Rik	Missense	NM_001033385	c.T1897G	p.L633V	0.393
A2	A2-1-1	chr10	51590152	51590152	G	A	Fam162b	Missense	NM_029894	c.C175T	p.P59S	0.514
A2	A2-1-1	chr11	87871456	87871456	G	A	Epx	Missense	NM_007946	c.C968T	p.A323V	0.189
A2	A2-1-1	chr11	69400041	69400041	C	T	Kdm6b	Missense	NM_001017426	c.G4801A	p.E1601K	0.49
A2	A2-1-1	chr15	94283837	94283837	T	-	Adamts20	Frameshift deletion	NM_177431	c.4974delA	p.K1658fs	0.333
A2	A2-1-1	chr15	98544040	98544040	G	A	Ccnt1	Missense	NM_009833	c.C1346T	p.P449L	0.464
A2	A2-1-1	chr18	60270062	60270062	G	A	Gm4841	Missense	NM_001034859	c.C958T	p.L320F	0.491
A2	A2-1-1	chr19	47665872	47665872	C	T	Col17a1	Missense	NM_007732	c.G1945A	p.E649K	0.5
A2	A2-1-1	chr19	33168452	33168452	G	T	Rnls	Missense	NM_001167818	c.C620A	p.P207Q	0.424
A2	A2-1-1	chrX	6083060	6083060	A	C	AU022751	Missense	NM_001166433	c.T263G	p.L88R	0.23
A2	A2-2-1	chr2	35354896	35354896	T	A	4930568D16Rik	Missense	NM_029463	c.A443T	p.K148I	0.453
A2	A2-2-1	chr2	88411871	88411871	T	A	Olfr1180	Missense	NM_146918	c.A786T	p.E262D	0.516
A2	A2-2-1	chr2	153243656	153243656	C	T	Pofut1	Missense	NM_080463	c.C158T	p.A53V	0.42
A2	A2-2-1	chr3	131293658	131293658	C	T	Cyp2u1	Missense	NM_027816	c.G1273A	p.G425S	0.389
A2	A2-2-1	chr4	135750763	135750763	G	A	Il22ra1	Missense	NM_178257	c.G1144A	p.G382R	0.534
A2	A2-2-1	chr6	83652425	83652425	C	T	Clec4f	Missense	NM_016751	c.G1150A	p.E384K	0.5
A2	A2-2-1	chr6	140593564	140593577	TGTCCCCGTCTCCT	-	Plekha5	Frameshift deletion	NM_144920	c.3725_3738del	p.L1242fs	0.163
A2	A2-2-1	chr6	48835525	48835525	G	A	Tmem176b	Missense	NM_001164208	c.C505T	p.P169S	0.074
A2	A2-2-1	chr6	47820441	47820441	T	C	Zfp786	Missense	NM_177882	c.A1562G	p.E521G	0.464
A2	A2-2-1	chr7	119595998	119595998	G	A	Acsm2	Missense	NM_001177978	c.G1732A	p.G578R	0.682
A2	A2-2-1	chr7	130503754	130503754	G	A	Ate1	Nonsense	NM_001136054	c.C904T	p.Q302X	0.517
A2	A2-2-1	chr7	81974048	81974048	G	A	Bnc1	Missense	NM_007562	c.C1430T	p.P477L	0.475
A2	A2-2-1	chr9	78414233	78414233	G	A	Ddx43	Missense	NM_001191044	c.G1183A	p.E395K	0.342
A2	A2-2-1	chr9	44263885	44263885	G	A	Nlrx1	Missense	NM_001163742	c.C593T	p.S198F	0.513
A2	A2-2-1	chr10	56162043	56162043	A	C	D630037F22Rik	Missense	NM_001033385	c.T1897G	p.L633V	0.462
A2	A2-2-1	chr10	51590152	51590152	G	A	Fam162b	Missense	NM_029894	c.C175T	p.P59S	0.446
A2	A2-2-1	chr11	87871456	87871456	G	A	Epx	Missense	NM_007946	c.C968T	p.A323V	0.241
A2	A2-2-1	chr11	69400041	69400041	C	T	Kdm6b	Missense	NM_001017426	c.G4801A	p.E1601K	0.494
A2	A2-2-1	chr15	94283837	94283837	T	-	Adamts20	Frameshift deletion	NM_177431	c.4974delA	p.K1658fs	0.286
A2	A2-2-1	chr15	98544040	98544040	G	A	Ccnt1	Missense	NM_009833	c.C1346T	p.P449L	0.555
A2	A2-2-1	chr18	60270062	60270062	G	A	Gm4841	Missense	NM_001034859	c.C958T	p.L320F	0.618
A2	A2-2-1	chr19	47665872	47665872	C	T	Col17a1	Missense	NM_007732	c.G1945A	p.E649K	0.479
A2	A2-2-1	chr19	33168452	33168452	G	T	Rnls	Missense	NM_001167818	c.C620A	p.P207Q	0.375
A2	A2-2-1	chrX	6083060	6083060	A	C	AU022751	Missense	NM_001166433	c.T263G	p.L88R	0.204
A2	A2-2-2	chr2	35354896	35354896	T	A	4930568D16Rik	Missense	NM_029463	c.A443T	p.K148I	0.542
A2	A2-2-2	chr2	88411871	88411871	T	A	Olfr1180	Missense	NM_146918	c.A786T	p.E262D	0.49
A2	A2-2-2	chr2	153243656	153243656	C	T	Pofut1	Missense	NM_080463	c.C158T	p.A53V	0.508
A2	A2-2-2	chr3	131293658	131293658	C	T	Cyp2u1	Missense	NM_027816	c.G1273A	p.G425S	0.4
A2	A2-2-2	chr4	135750763	135750763	G	A	Il22ra1	Missense	NM_178257	c.G1144A	p.G382R	0.494
A2	A2-2-2	chr6	23115156	23115156	C	T	Aass	Splice site	NM_013930	c.473-1G>A	NA	0.647
A2	A2-2-2	chr6	83652425	83652425	C	T	Clec4f	Missense	NM_016751	c.G1150A	p.E384K	0.496
A2	A2-2-2	chr6	140593564	140593577	TGTCCCCGTCTCCT	-	Plekha5	Frameshift deletion	NM_144920	c.3725_3738del	p.L1242fs	0.143
A2	A2-2-2	chr6	47820441	47820441	T	C	Zfp786	Missense	NM_177882	c.A1562G	p.E521G	0.5
A2	A2-2-2	chr7	119595998	119595998	G	A	Acsm2	Missense	NM_001177978	c.G1732A	p.G578R	0.447
A2	A2-2-2	chr7	130503754	130503754	G	A	Ate1	Nonsense	NM_001136054	c.C904T	p.Q302X	0.425
A2	A2-2-2	chr7	81974048	81974048	G	A	Bnc1	Missense	NM_007562	c.C1430T	p.P477L	0.439
A2	A2-2-2	chr9	78414233	78414233	G	A	Ddx43	Missense	NM_001191044	c.G1183A	p.E395K	0.526
A2	A2-2-2	chr9	44263885	44263885	G	A	Nlrx1	Missense	NM_001163742	c.C593T	p.S198F	0.477
A2	A2-2-2	chr10	56162043	56162043	A	C	D630037F22Rik	Missense	NM_001033385	c.T1897G	p.L633V	0.462
A2	A2-2-2	chr10	51590152	51590152	G	A	Fam162b	Missense	NM_029894	c.C175T	p.P59S	0.573
A2	A2-2-2	chr11	87871456	87871456	G	A	Epx	Missense	NM_007946	c.C968T	p.A323V	0.181
A2	A2-2-2	chr11	69400041	69400041	C	T	Kdm6b	Missense	NM_001017426	c.G4801A	p.E1601K	0.5
A2	A2-2-2	chr15	94283837	94283837	T	-	Adamts20	Frameshift deletion	NM_177431	c.4974delA	p.K1658fs	0.291
A2	A2-2-2	chr15	98544040	98544040	G	A	Ccnt1	Missense	NM_009833	c.C1346T	p.P449L	0.424
A2	A2-2-2	chr18	60270062	60270062	G	A	Gm4841	Missense	NM_001034859	c.C958T	p.L320F	0.521

A2	A2-2-2	chr19	47665872	47665872	C	T	Col17a1	Missense	NM_007732	c.G1945A	p.E649K	0.586
A2	A2-2-2	chr19	33168452	33168452	G	T	Rnls	Missense	NM_001167818	c.C620A	p.P207Q	0.417
A2	A2-2-2	chrX	6083060	6083060	A	C	AU022751	Missense	NM_001166433	c.T263G	p.L88R	0.195
B1	B1-1-1	chr3	92868846	92868846	C	A	2310050C09Rik	Nonsense	NM_025621	c.G529T	p.G177X	0.118
B1	B1-1-1	chr11	43776006	43776006	A	G	Adra1b	Missense	NM_007416	c.T1403C	p.L468P	0.38
B1	B1-1-1	chr14	102981774	102981774	C	T	Kctd12	Missense	NM_177715	c.G667A	p.G223R	0.315
B1	B1-1-1	chrX	96160175	96160175	C	T	Msn	Missense	NM_010833	c.C883T	p.R295C	0.477
B1	B1-2-1	chr11	43776006	43776006	A	G	Adra1b	Missense	NM_007416	c.T1403C	p.L468P	0.277
B1	B1-2-1	chr11	70318181	70318181	A	C	Alox12e	Missense	NM_145684	c.T1238G	p.I413S	0.138
B1	B1-2-1	chr14	102981774	102981774	C	T	Kctd12	Missense	NM_177715	c.G667A	p.G223R	0.282
B1	B1-2-1	chrX	96160175	96160175	C	T	Msn	Missense	NM_010833	c.C883T	p.R295C	0.451
B1	B1-2-2	chr1	71047666	71047666	T	G	Bard1	Missense	NM_007525	c.A1748C	p.K583T	0.194
B1	B1-2-2	chr3	92868846	92868846	C	A	2310050C09Rik	Nonsense	NM_025621	c.G529T	p.G177X	0.092
B1	B1-2-2	chr5	137529884	137529884	C	T	Gnb2	Missense	NM_010312	c.G229A	p.G77R	0.11
B1	B1-2-2	chr11	43776006	43776006	A	G	Adra1b	Missense	NM_007416	c.T1403C	p.L468P	0.321
B1	B1-2-2	chr11	70318181	70318181	A	C	Alox12e	Missense	NM_145684	c.T1238G	p.I413S	0.133
B1	B1-2-2	chr14	102981774	102981774	C	T	Kctd12	Missense	NM_177715	c.G667A	p.G223R	0.314
B1	B1-2-2	chrX	96160175	96160175	C	T	Msn	Missense	NM_010833	c.C883T	p.R295C	0.367
B1	B1-2-3	chr1	71047666	71047666	T	G	Bard1	Missense	NM_007525	c.A1748C	p.K583T	0.526
B1	B1-2-3	chr3	92868846	92868846	C	A	2310050C09Rik	Nonsense	NM_025621	c.G529T	p.G177X	0.162
B1	B1-2-3	chr5	137529884	137529884	C	T	Gnb2	Missense	NM_010312	c.G229A	p.G77R	0.165
B1	B1-2-3	chr11	43776006	43776006	A	G	Adra1b	Missense	NM_007416	c.T1403C	p.L468P	0.311
B1	B1-2-3	chr11	70318181	70318181	A	C	Alox12e	Missense	NM_145684	c.T1238G	p.I413S	0.155
B1	B1-2-3	chr14	102981774	102981774	C	T	Kctd12	Missense	NM_177715	c.G667A	p.G223R	0.301
B1	B1-2-3	chrX	96160175	96160175	C	T	Msn	Missense	NM_010833	c.C883T	p.R295C	0.428
B1	B1-3-1	chr5	137529884	137529884	C	T	Gnb2	Missense	NM_010312	c.G229A	p.G77R	0.397
B1	B1-3-1	chr7	79098085	79098085	A	C	Acan	Missense	NM_007424	c.A2603C	p.E868A	0.091
B1	B1-3-1	chr11	43776006	43776006	A	G	Adra1b	Missense	NM_007416	c.T1403C	p.L468P	0.224
B1	B1-3-1	chr14	102981774	102981774	C	T	Kctd12	Missense	NM_177715	c.G667A	p.G223R	0.213
B1	B1-3-1	chrX	96160175	96160175	C	T	Msn	Missense	NM_010833	c.C883T	p.R295C	0.408
B1	B1-3-1	chrX	64272307	64272307	C	A	Slitrk4	Missense	NM_178740	c.G753T	p.R251S	0.12
B1	B1-3-2	chr1	71047666	71047666	T	G	Bard1	Missense	NM_007525	c.A1748C	p.K583T	0.158
B1	B1-3-2	chr5	137529884	137529884	C	T	Gnb2	Missense	NM_010312	c.G229A	p.G77R	0.432
B1	B1-3-2	chr11	43776006	43776006	A	G	Adra1b	Missense	NM_007416	c.T1403C	p.L468P	0.198
B1	B1-3-2	chr14	102981774	102981774	C	T	Kctd12	Missense	NM_177715	c.G667A	p.G223R	0.166
B1	B1-3-2	chr14	76134837	76134837	C	T	Nufip1	Nonsense	NM_013745	c.C1390T	p.R464X	0.07
B1	B1-3-2	chrX	96160175	96160175	C	T	Msn	Missense	NM_010833	c.C883T	p.R295C	0.269
B1	B1-3-4	chr1	71047666	71047666	T	G	Bard1	Missense	NM_007525	c.A1748C	p.K583T	0.367
B1	B1-3-4	chr2	74705975	74705975	A	T	Hoxd8	Missense	NM_008276	c.A428T	p.Q143L	0.104
B1	B1-3-4	chr2	59842945	59842945	G	A	Tanc1	Missense	NM_198294	c.G4393A	p.E1465K	0.112
B1	B1-3-4	chr4	115920216	115920216	T	A	Dmbx1	Missense	NM_001025567	c.A434T	p.E145V	0.094
B1	B1-3-4	chr4	120097199	120097199	T	G	Hivep3	Missense	NM_010657	c.T2711G	p.F904C	0.173
B1	B1-3-4	chr4	81335755	81335755	C	T	Mpdz	Missense	NM_010820	c.G3139A	p.E1047K	0.1
B1	B1-3-4	chr5	90474665	90474665	A	G	Alb	Missense	NM_009654	c.A1751G	p.K584R	0.076
B1	B1-3-4	chr5	137529884	137529884	C	T	Gnb2	Missense	NM_010312	c.G229A	p.G77R	0.229
B1	B1-3-4	chr8	110415777	110415777	G	A	Hydin	Missense	NM_172916	c.G2287A	p.E763K	0.167
B1	B1-3-4	chr11	43776006	43776006	A	G	Adra1b	Missense	NM_007416	c.T1403C	p.L468P	0.222
B1	B1-3-4	chr11	69744685	69744685	G	A	Polr2a	Missense	NM_009089	c.C1583T	p.P528L	0.09
B1	B1-3-4	chr13	104375757	104375757	G	A	Adamts6	Missense	NM_001081020	c.G1486A	p.A496T	0.213
B1	B1-3-4	chr14	102981774	102981774	C	T	Kctd12	Missense	NM_177715	c.G667A	p.G223R	0.277
B1	B1-3-4	chr14	75525901	75525901	C	A	Siah3	Missense	NM_001128093	c.C591A	p.F197L	0.153
B1	B1-3-4	chr15	66919401	66919401	G	A	Wisp1	Missense	NM_018865	c.G1051A	p.D351N	0.087
B1	B1-3-4	chr17	31105597	31105597	C	T	Abcg1	Missense	NM_009593	c.C815T	p.T272I	0.074
B1	B1-3-4	chrX	105875297	105875297	T	G	Atrx	Missense	NM_009530	c.A2936C	p.K979T	0.098
B1	B1-3-4	chrX	96160175	96160175	C	T	Msn	Missense	NM_010833	c.C883T	p.R295C	0.242
B1	B1-3-4	chrX	64272307	64272307	C	A	Slitrk4	Missense	NM_178740	c.G753T	p.R251S	0.116
B1	B1-3-5	chr2	74705975	74705975	A	T	Hoxd8	Missense	NM_008276	c.A428T	p.Q143L	0.092
B1	B1-3-5	chr5	137529884	137529884	C	T	Gnb2	Missense	NM_010312	c.G229A	p.G77R	0.363
B1	B1-3-5	chr9	45752068	45752068	C	T	Dscaml1	Missense	NM_001081270	c.C5588T	p.S1863F	0.097
B1	B1-3-5	chr11	43776006	43776006	A	G	Adra1b	Missense	NM_007416	c.T1403C	p.L468P	0.225
B1	B1-3-5	chr14	102981774	102981774	C	T	Kctd12	Missense	NM_177715	c.G667A	p.G223R	0.221
B1	B1-3-5	chr17	31105597	31105597	C	T	Abcg1	Missense	NM_009593	c.C815T	p.T272I	0.076
B1	B1-3-5	chrX	96160175	96160175	C	T	Msn	Missense	NM_010833	c.C883T	p.R295C	0.259
B1	B1-3-5	chrX	64272307	64272307	C	A	Slitrk4	Missense	NM_178740	c.G753T	p.R251S	0.094
B1	B1-4-1	chr1	71047666	71047666	T	G	Bard1	Missense	NM_007525	c.A1748C	p.K583T	0.241
B1	B1-4-1	chr1	34278049	34278049	A	T	Dst	Missense	NM_001276764	c.A20481T	p.E6827D	0.145
B1	B1-4-1	chr3	62493796	62493796	G	A	Dhx36	Nonsense	NM_028136	c.C1048T	p.R350X	0.135
B1	B1-4-1	chr3	92028012	92028012	C	T	Pglyrp3	Missense	NM_207247	c.C568T	p.R190W	0.3
B1	B1-4-1	chr5	137529884	137529884	C	T	Gnb2	Missense	NM_010312	c.G229A	p.G77R	0.771
B1	B1-4-1	chr6	13836698	13836698	C	T	Gpr85	Missense	NM_145066	c.G206A	p.R69K	0.08
B1	B1-4-1	chr6	121358699	121358699	C	G	Slc6a12	Missense	NM_133661	c.C987G	p.C329W	0.154
B1	B1-4-1	chr7	140297699	140297699	C	T	5830411N06Rik	Missense	NM_001128146	c.C2671T	p.L891F	0.079
B1	B1-4-1	chr9	108084849	108084849	G	A	Mst1	Missense	NM_008243	c.G2065A	p.V689M	0.155
B1	B1-4-1	chr10	94181503	94181503	C	T	Nr2c1	Missense	NM_011629	c.C1102T	p.P368S	0.08
B1	B1-4-1	chr11	43776006	43776006	A	G	Adra1b	Missense	NM_007416	c.T1403C	p.L468P	0.141
B1	B1-4-1	chr12	76575914	76575914	C	T	Plekhg3	Missense	NM_153804	c.C1930T	p.P644S	0.078
B1	B1-4-1	chr13	93677100	93677100	G	A	Dmgdh	Missense	NM_028772	c.G152A	p.G51D	0.135
B1	B1-4-1	chr14	102981774	102981774	C	T	Kctd12	Missense	NM_177715	c.G667A	p.G223R	0.137
B1	B1-4-1	chr14	76134837	76134837	C	T	Nufip1	Nonsense	NM_013745	c.C1390T	p.R464X	0.083
B1	B1-4-1	chr18	20536959	20536959	G	A	Dsg3	Missense	NM_030596	c.G2029A	p.E677K	0.157
B1	B1-4-1	chr19	37685837	37685837	C	T	Cyp26c1	Missense	NM_001105201	c.C158T	p.S53F	0.077
B1	B1-4-1	chrX	96160175	96160175	C	T	Msn	Missense	NM_010833	c.C883T	p.R295C	0.116
B1	B1-4-5	chr1	172239796	172239796	C	T	Atp1a4	Missense	NM_013734	c.G1768A	p.E590K	0.161
B1	B1-4-5	chr1	171237964	171237964	G	A	Ndufs2	Nonsense	NM_153064	c.C967T	p.R323X	0.11
B1	B1-4-5	chr2	65022586	65022586	G	A	Grb14	Missense	NM_016719	c.C59T	p.S20L	0.159
B1	B1-4-5	chr3	62493796	62493796	G	A	Dhx36	Nonsense	NM_028136	c.C1048T	p.R350X	0.137
B1	B1-4-5	chr5	137529884	137529884	C	T	Gnb2	Missense	NM_010312	c.G229A	p.G77R	0.718
B1	B1-4-5	chr5	142776308	142776308	G	A	Tnrc18	Missense	NM_001122730	c.C2110T	p.L704F	0.071
B1	B1-4-5	chr6	121358699	121358699	C	G	Slc6a12	Missense	NM_133661	c.C987G	p.C329W	0.183
B1	B1-4-5	chr7	6277419	6277419	G	A	Gm6792	Splice site	NM_001177416	c.277-1G>A	NA	0.114
B1	B1-4-5	chr7	104704475	104704475	G	A	Olfr663	Missense	NM_001011757	c.G907A	p.E303K	0.133
B1	B1-4-5	chr8	4210997	4210997	G	A	BC068157	Missense	NM_207203	c.C2819T	p.S940F	0.083
B1	B1-4-5	chr9	4299348	4299348	G	A	Aasdhppt	Missense	NM_026276	c.C715T	p.H239Y	0.091
B1	B1-4-5	chr9	108084849	108084849	G	A	Mst1	Missense	NM_008243	c.G2065A	p.V689M	0.121
B1	B1-4-5	chr11	43776006	43776006	A	G	Adra1b	Missense	NM_007416	c.T1403C	p.L468P	0.107
B1	B1-4-5	chr11	12249810	12249810	G	A	Cobl	Missense	NM_172496	c.C3836T	p.P1279L	0.087
B1	B1-4-5	chr13	93677100	93677100	G	A	Dmgdh	Missense	NM_028772	c.G152A	p.G51D	0.166
B1	B1-4-5	chr13	64921751	64921751	T	A	Spata31	Missense	NM_030047	c.T1712A	p.M571K	0.079
B1	B1-4-5	chr14	76134837	76134837	C	T	Nufip1	Nonsense	NM_013745	c.C1390T	p.R464X	0.174
B1	B1-4-5	chr17	37151568	37151568	G	A	Olfr93	Missense	NM_001011813	c.C403T	p.H135Y	0.176
B1	B1-4-5	chr18	13717655	13717655	C	A	Zfp521	Missense	NM_145492	c.G3751T	p.G1251W	0.148
B1	B1-4-9	chr2	74705975	74705975	A	T	Hoxd8	Missense	NM_008276	c.A428T	p.Q143L	0.246
B1	B1-4-9	chr2	59842945	59842945	G	A	Tanc1	Missense	NM_198294	c.G4393A	p.E1465K	0.208
B1	B1-4-9	chr3	36870118	36870118	C	T	4932438A13Rik	Missense	NM_172679	c.C91T	p.L31F	0.286
B1	B1-4-9	chr4	115920216	115920216	T	A	Dmbx1	Missense	NM_001025567	c.A434T	p.E145V	0.288
B1	B1-4-9	chr4	120097199	120097199	T	G	Hivep3	Missense	NM_010657	c.T2711G	p.F904C	0.381
B1	B1-4-9	chr4	81335755	81335755	C	T	Mpdz	Missense	NM_010820	c.G3139A	p.E1047K	0.333
B1	B1-4-9	chr5	90474665	90474665	A	G	Alb	Missense	NM_009654	c.A1751G	p.K584R	0.281
B1	B1-4-9	chr5	137529884	137529884	C	T	Gnb2	Missense	NM_010312	c.G229A	p.G77R	0.155
B1	B1-4-9	chr8	80496720	80496720	C	T	Gypa	Missense	NM_010369	c.C124T	p.L42F	0.339
B1	B1-4-9	chr8	110415777	110415777	G	A	Hydin	Missense	NM_172916	c.G2287A	p.E763K	0.35
B1	B1-4-9	chr9	45752068	45752068	C	T	Dscaml1	Missense	NM_001081270	c.C5588T	p.S1863F	0.191
B1	B1-4-9	chr9	62696892	62696892	G	C	Itga11	Missense	NM_176922	c.G61C	p.D21H	0.269
B1	B1-4-9	chr11	43776006	43776006	A	G	Adra1b	Missense	NM_007416	c.T1403C	p.L468P	0.093
B1	B1-4-9	chr11	69744685	69744685	G	A	Polr2a	Missense	NM_009089	c.C1583T	p.P528L	0.424
B1	B1-4-9	chr12	73941721	73941721	T	G	Hif1a	Missense	NM_010431	c.T1567G	p.F523V	0.205
B1	B1-4-9	chr13	104375757	104375757	G	A	Adamts6	Missense	NM_001081020	c.G1486A	p.A496T	0.282
B1	B1-4-9	chr14	102981774	102981774	C	T	Kctd12	Missense	NM_177715	c.G667A	p.G223R	0.07
B1	B1-4-9	chr14	75525901	75525901	C	A	Siah3	Missense	NM_001128093	c.C591A	p.F197L	0.258
B1	B1-4-9	chr15	77786512	77786512	G	A	Myh9	Missense	NM_022410	c.C1529T	p.P510L	0.279
B1	B1-4-9	chr15	66919401	66919401	G	A	Wisp1	Missense	NM_018865	c.G1051A	p.D351N	0.31
B1	B1-4-9	chr17	31105597	31105597	C	T	Abcg1	Missense	NM_009593	c.C815T	p.T272I	0.33
B1	B1-4-9	chr19	58733459	58733459	T	G	Pnliprp1	Missense	NM_018874	c.T569G	p.I190S	0.302
B1	B1-4-9	chrX	105875297	105875297	T	G	Atrx	Missense	NM_009530	c.A2936C	p.K979T	0.254

B1	B1-4-9	chrX	96160175	96160175	C	T	Msn	Missense	NM_010833	c.C883T	p.R295C	0.126
B1	B1-4-10	chr2	74705975	74705975	A	T	Hoxd8	Missense	NM_008276	c.A428T	p.Q143L	0.206
B1	B1-4-10	chr2	59842945	59842945	G	A	Tanc1	Missense	NM_198294	c.G4393A	p.E1465K	0.203
B1	B1-4-10	chr4	115920216	115920216	T	A	Dmbx1	Missense	NM_001025567	c.A434T	p.E145V	0.227
B1	B1-4-10	chr4	120097199	120097199	T	G	Hivep3	Missense	NM_010657	c.T2711G	p.F904C	0.228
B1	B1-4-10	chr4	81335755	81335755	C	T	Mpdz	Missense	NM_010820	c.G3139A	p.E1047K	0.293
B1	B1-4-10	chr5	90474665	90474665	A	G	Alb	Missense	NM_009654	c.A1751G	p.K584R	0.194
B1	B1-4-10	chr5	137529884	137529884	C	T	Gnb2	Missense	NM_010312	c.G229A	p.G77R	0.145
B1	B1-4-10	chr7	105144072	105144072	T	G	Olfr683	Missense	NM_147045	c.A238C	p.I80L	0.346
B1	B1-4-10	chr8	80496720	80496720	C	T	Gypa	Missense	NM_010369	c.C124T	p.L42F	0.22
B1	B1-4-10	chr8	110415777	110415777	G	A	Hydin	Missense	NM_172916	c.G2287A	p.E763K	0.209
B1	B1-4-10	chr9	45752068	45752068	C	T	Dscaml1	Missense	NM_001081270	c.C5588T	p.S1863F	0.218
B1	B1-4-10	chr9	62696892	62696892	G	C	Itga11	Missense	NM_176922	c.G61C	p.D21H	0.203
B1	B1-4-10	chr11	43776006	43776006	A	G	Adra1b	Missense	NM_007416	c.T1403C	p.L468P	0.193
B1	B1-4-10	chr11	69744685	69744685	G	A	Polr2a	Missense	NM_009089	c.C1583T	p.P528L	0.17
B1	B1-4-10	chr12	73941721	73941721	T	G	Hif1a	Missense	NM_010431	c.T1567G	p.F523V	0.164
B1	B1-4-10	chr14	102981774	102981774	C	T	Kctd12	Missense	NM_177715	c.G667A	p.G223R	0.177
B1	B1-4-10	chr14	75525901	75525901	C	A	Siah3	Missense	NM_001128093	c.C591A	p.F197L	0.15
B1	B1-4-10	chr15	66919401	66919401	G	A	Wisp1	Missense	NM_018865	c.G1051A	p.D351N	0.232
B1	B1-4-10	chr17	31105597	31105597	C	T	Abcg1	Missense	NM_009593	c.C815T	p.T272I	0.255
B1	B1-4-10	chr19	58733459	58733459	T	G	Pnliprp1	Missense	NM_018874	c.T569G	p.I190S	0.352
B1	B1-4-10	chrX	105875297	105875297	T	G	Atrx	Missense	NM_009530	c.A2936C	p.K979T	0.191
B1	B1-4-10	chrX	96160175	96160175	C	T	Msn	Missense	NM_010833	c.C883T	p.R295C	0.126
B1	B1-4-13	chr2	155276972	155276972	G	A	Map1lc3a	Missense	NM_025735	c.G55A	p.E19K	0.093
B1	B1-4-13	chr2	126829167	126829167	T	A	Trpm7	Missense	NM_021450	c.A1894T	p.M632L	0.231
B1	B1-4-13	chr4	115920216	115920216	T	A	Dmbx1	Missense	NM_001025567	c.A434T	p.E145V	0.122
B1	B1-4-13	chr4	120097199	120097199	T	G	Hivep3	Missense	NM_010657	c.T2711G	p.F904C	0.091
B1	B1-4-13	chr4	141683988	141683988	G	T	Rsc1a1	Missense	NM_023544	c.C1612A	p.P538T	0.093
B1	B1-4-13	chr5	90474665	90474665	A	G	Alb	Missense	NM_009654	c.A1751G	p.K584R	0.105
B1	B1-4-13	chr5	114263107	114263107	G	A	Foxn4	Missense	NM_148935	c.C134T	p.S45L	0.102
B1	B1-4-13	chr5	137529884	137529884	C	T	Gnb2	Missense	NM_010312	c.G229A	p.G77R	0.591
B1	B1-4-13	chr8	110415777	110415777	G	A	Hydin	Missense	NM_172916	c.G2287A	p.E763K	0.114
B1	B1-4-13	chr9	92343681	92343681	C	T	1700057G04Rik	Missense	NM_001033184	c.C55T	p.H19Y	0.167
B1	B1-4-13	chr9	62696892	62696892	G	C	Itga11	Missense	NM_176922	c.G61C	p.D21H	0.1
B1	B1-4-13	chr11	43776006	43776006	A	G	Adra1b	Missense	NM_007416	c.T1403C	p.L468P	0.092
B1	B1-4-13	chr11	55532341	55532341	G	A	Glra1	Nonsense	NM_020492	c.C640T	p.Q214X	0.112
B1	B1-4-13	chr11	103208861	103208861	C	T	Spata32	Missense	NM_177801	c.G817A	p.D273N	0.125
B1	B1-4-13	chr14	70638916	70638916	G	C	Fgf17	Missense	NM_008004	c.C136G	p.Q46E	0.09
B1	B1-4-13	chr14	102981774	102981774	C	T	Kctd12	Missense	NM_177715	c.G667A	p.G223R	0.099
B1	B1-4-13	chr15	66919401	66919401	G	A	Wisp1	Missense	NM_018865	c.G1051A	p.D351N	0.194
B1	B1-4-13	chr16	93810163	93810163	C	T	Dopey2	Missense	NM_027293	c.C6704T	p.S2235F	0.083
B1	B1-4-13	chr18	82986882	82986882	C	A	Zfp516	Missense	NM_001177464	c.C1910A	p.S637Y	0.089
B1	B1-4-13	chr19	58733459	58733459	T	G	Pnliprp1	Missense	NM_018874	c.T569G	p.I190S	0.22
B1	B1-4-13	chrX	105875297	105875297	T	G	Atrx	Missense	NM_009530	c.A2936C	p.K979T	0.105
B1	B1-4-13	chrX	96160175	96160175	C	T	Msn	Missense	NM_010833	c.C883T	p.R295C	0.124
C1	C1-1-1	chr8	110858656	110858656	G	A	Cog4	Missense	NM_133973	c.G952A	p.V318I	0.533
C1	C1-2-1	chr5	121168003	121168003	C	T	Ptpn11	Missense	NM_011202	c.G178A	p.G60R	0.276
C1	C1-2-1	chr8	110858656	110858656	G	A	Cog4	Missense	NM_133973	c.G952A	p.V318I	0.585
C1	C1-2-2	chr2	173568941	173568941	G	A	Ankrd60	Missense	NM_027303	c.C785T	p.T262M	0.085
C1	C1-2-2	chr5	122457255	122457255	C	T	Atp2a2	Missense	NM_001110140	c.G3031A	p.A1011T	0.077
C1	C1-2-2	chr5	121168003	121168003	C	T	Ptpn11	Missense	NM_011202	c.G178A	p.G60R	0.323
C1	C1-2-2	chr8	110858656	110858656	G	A	Cog4	Missense	NM_133973	c.G952A	p.V318I	0.456
C2	C2-1-1	chr6	39627783	39627783	A	T	Braf	Missense	NM_139294	c.T1910A	p.V637E	0.453
C2	C2-1-1	chr7	57408803	57408803	G	A	Gabra5	Missense	NM_176942	c.C1196T	p.A399V	0.439
C2	C2-2-1	chr2	25295931	25295931	A	C	Grin1	Missense	NM_008169	c.T2450G	p.F817C	0.078
C2	C2-2-1	chr6	39627783	39627783	A	T	Braf	Missense	NM_139294	c.T1910A	p.V637E	0.394
C2	C2-2-1	chr7	57408803	57408803	G	A	Gabra5	Missense	NM_176942	c.C1196T	p.A399V	0.524
C2	C2-2-2	chr6	39627783	39627783	A	T	Braf	Missense	NM_139294	c.T1910A	p.V637E	0.63
C2	C2-2-2	chr7	57408803	57408803	G	A	Gabra5	Missense	NM_176942	c.C1196T	p.A399V	0.423
D1	D1-1-1	chr1	43742308	43742308	G	A	1500015O10Rik	Missense	NM_024283	c.G349A	p.D117N	0.575
D1	D1-1-1	chr1	58233915	58233915	G	A	Aox4	Missense	NM_023631	c.G988A	p.E330K	0.381
D1	D1-1-1	chr1	160577443	160577443	T	A	Gpr52	Missense	NM_001146330	c.A311T	p.Y104F	0.58
D1	D1-1-1	chr2	83879660	83879660	A	G	Fam171b	Missense	NM_175514	c.A1675G	p.R559G	0.481
D1	D1-1-1	chr2	41449323	41449323	T	A	Lrp1b	Missense	NM_053011	c.A2507T	p.N836I	0.5
D1	D1-1-1	chr2	87753589	87753589	C	T	Olfr1141	Missense	NM_146637	c.G403A	p.D135N	0.496
D1	D1-1-1	chr3	87975556	87975556	A	G	Nes	Missense	NM_016701	c.A1121G	p.K374R	0.5
D1	D1-1-1	chr3	102941078	102941078	A	G	Nr1h5	Missense	NM_198658	c.T1310C	p.L437P	0.442
D1	D1-1-1	chr3	109827654	109827654	C	T	Ntng1	Missense	NM_030699	c.G1363A	p.G455R	0.514
D1	D1-1-1	chr3	129811729	129811729	G	A	Rrh	Missense	NM_009102	c.C602T	p.P201L	0.422
D1	D1-1-1	chr3	5412423	5412423	G	A	Zfhx4	Missense	NM_030708	c.G10022A	p.S3341N	0.481
D1	D1-1-1	chr4	101814920	101814920	C	T	Lepr	Missense	NM_146146	c.C3140T	p.S1047L	0.47
D1	D1-1-1	chr4	43101826	43101826	T	A	Unc13b	Nonsense	NM_021468	c.T420A	p.Y140X	0.517
D1	D1-1-1	chr5	101814467	101814467	C	T	Cds1	Missense	NM_173370	c.C947T	p.S316F	0.436
D1	D1-1-1	chr5	4757484	4757484	C	T	Fzd1	Missense	NM_021457	c.G97A	p.G33R	0.419
D1	D1-1-1	chr5	106845458	106845458	C	T	Hfm1	Missense	NM_177873	c.G3898A	p.D1300N	0.468
D1	D1-1-1	chr5	33668158	33668158	C	T	Tacc3	Missense	NM_001040435	c.C1231T	p.L411F	0.5
D1	D1-1-1	chr6	142449487	142449487	A	T	Gys2	Missense	NM_145572	c.T1251A	p.N417K	0.446
D1	D1-1-1	chr7	103451408	103451408	G	A	Olfr606	Missense	NM_147094	c.G70A	p.E24K	0.286
D1	D1-1-1	chr7	35584548	35584548	C	A	Rgs9bp	Missense	NM_145840	c.G673T	p.V225L	0.51
D1	D1-1-1	chr8	25739570	25739570	C	T	Ddhd2	Missense	NM_028102	c.G1141A	p.A381T	0.25
D1	D1-1-1	chr8	33572140	33572140	C	T	Tex15	Missense	NM_031374	c.C1597T	p.L533F	0.5
D1	D1-1-1	chr9	43279886	43279886	G	A	D630033O11Rik	Missense	NM_001243261	c.G348A	p.M116I	0.688
D1	D1-1-1	chr9	3634462	3634462	G	A	Gucy1a2	Missense	NM_001033322	c.G505A	p.E169K	0.382
D1	D1-1-1	chr9	38403692	38403692	G	T	Olfr147	Nonsense	NM_146869	c.G817T	p.G273X	0.399
D1	D1-1-1	chr9	78313067	78313067	G	A	Omt2a	Missense	NM_001164523	c.C49T	p.L17F	0.449
D1	D1-1-1	chr9	48832304	48832304	G	A	Zbtb16	Missense	NM_001033324	c.C707T	p.P236L	0.564
D1	D1-1-1	chr10	89714952	89714952	G	A	Actr6	Missense	NM_025914	c.C979T	p.P327S	0.615
D1	D1-1-1	chr10	128953950	128953950	C	A	Itga7	Missense	NM_008398	c.C3059A	p.P1020Q	0.534
D1	D1-1-1	chr10	88548796	88548796	G	A	Mybpc1	Missense	NM_001252372	c.C1625T	p.P542L	0.49
D1	D1-1-1	chr11	85178450	85178450	G	A	1700125H20Rik	Missense	NM_028589	c.G623A	p.G208E	0.532
D1	D1-1-1	chr11	75432578	75432578	C	T	Serpinf2	Missense	NM_008878	c.G1301A	p.R434K	0.421
D1	D1-1-1	chr12	3367043	3367043	C	T	Kif3c	Missense	NM_008445	c.C1063T	p.L355F	0.515
D1	D1-1-1	chr12	69722113	69722113	A	T	L2hgdh	Missense	NM_145443	c.T224A	p.L75H	0.537
D1	D1-1-1	chr13	92752398	92752398	G	A	Thbs4	Missense	NM_011582	c.C2786T	p.S929F	0.381
D1	D1-1-1	chr14	28522675	28522675	C	T	Wnt5a	Missense	NM_001256224	c.C818T	p.S273F	0.456
D1	D1-1-1	chr15	20666089	20666089	C	T	Acot10	Missense	NM_022816	c.G565A	p.D189N	0.43
D1	D1-1-1	chr15	98594461	98594461	G	A	Adcy6	Missense	NM_007405	c.C2921T	p.S974L	0.53
D1	D1-1-1	chr17	36128961	36128961	C	T	Gm7030	Missense	NM_001177467	c.G280A	p.E94K	0.458
D1	D1-1-1	chr17	71504803	71504803	A	C	Ndc80	Missense	NM_023294	c.T1427G	p.L476R	0.58
D1	D1-1-1	chr18	38254491	38254491	C	T	0610009O20Rik	Missense	NM_024179	c.C682T	p.L228F	0.445
D1	D1-1-1	chr18	7129517	7129517	T	A	Armc4	Missense	NM_001081393	c.A2660T	p.N887I	0.444
D1	D1-1-1	chr19	36057177	36057177	C	A	Htr7	Missense	NM_008315	c.G77T	p.R26L	0.51
D1	D1-1-1	chrX	100573860	100573860	G	A	Awat1	Nonsense	NM_001081136	c.G215A	p.W72X	0.477
D1	D1-1-1	chrX	129972594	129972594	C	T	Diap2	Missense	NM_172493	c.C2038T	p.P680S	0.495
D1	D1-1-1	chrX	152209512	152209512	C	T	Iqsec2	Missense	NM_001114664	c.C2144T	p.S715F	0.492
D1	D1-1-1	chrX	66654019	66654019	G	A	Slitrk2	Missense	NM_001161431	c.G115A	p.E39K	0.576
D1	D1-2-1	chr1	43742308	43742308	G	A	1500015O10Rik	Missense	NM_024283	c.G349A	p.D117N	0.415
D1	D1-2-1	chr1	58233915	58233915	G	A	Aox4	Missense	NM_023631	c.G988A	p.E330K	0.429
D1	D1-2-1	chr1	160577443	160577443	T	A	Gpr52	Missense	NM_001146330	c.A311T	p.Y104F	0.508
D1	D1-2-1	chr2	83879660	83879660	A	G	Fam171b	Missense	NM_175514	c.A1675G	p.R559G	0.502
D1	D1-2-1	chr2	41449323	41449323	T	A	Lrp1b	Missense	NM_053011	c.A2507T	p.N836I	0.515
D1	D1-2-1	chr2	87753589	87753589	C	T	Olfr1141	Missense	NM_146637	c.G403A	p.D135N	0.544
D1	D1-2-1	chr3	87975556	87975556	A	G	Nes	Missense	NM_016701	c.A1121G	p.K374R	0.505
D1	D1-2-1	chr3	102941078	102941078	A	G	Nr1h5	Missense	NM_198658	c.T1310C	p.L437P	0.574
D1	D1-2-1	chr3	109827654	109827654	C	T	Ntng1	Missense	NM_030699	c.G1363A	p.G455R	0.481
D1	D1-2-1	chr3	129811729	129811729	G	A	Rrh	Missense	NM_009102	c.C602T	p.P201L	0.443
D1	D1-2-1	chr3	5412423	5412423	G	A	Zfhx4	Missense	NM_030708	c.G10022A	p.S3341N	0.507
D1	D1-2-1	chr4	101814920	101814920	C	T	Lepr	Missense	NM_146146	c.C3140T	p.S1047L	0.459
D1	D1-2-1	chr4	43101826	43101826	T	A	Unc13b	Nonsense	NM_021468	c.T420A	p.Y140X	0.541
D1	D1-2-1	chr5	101814467	101814467	C	T	Cds1	Missense	NM_173370	c.C947T	p.S316F	0.545
D1	D1-2-1	chr5	4757484	4757484	C	T	Fzd1	Missense	NM_021457	c.G97A	p.G33R	0.466
D1	D1-2-1	chr5	106845458	106845458	C	T	Hfm1	Missense	NM_177873	c.G3898A	p.D1300N	0.408
D1	D1-2-1	chr5	33668158	33668158	C	T	Tacc3	Missense	NM_001040435	c.C1231T	p.L411F	0.407
D1	D1-2-1	chr6	142449487	142449487	A	T	Gys2	Missense	NM_145572	c.T1251A	p.N417K	0.485

D1	D1-2-1	chr7	108346731	108346732	AT	TA	Olfr493	Missense	NM_146310	c.AT248_249TA	p.N83I	0.643
D1	D1-2-1	chr7	103451408	103451408	G	A	Olfr606	Missense	NM_147094	c.G70A	p.E24K	0.451
D1	D1-2-1	chr7	35584548	35584548	C	A	Rgs9bp	Missense	NM_145840	c.G673T	p.V225L	0.48
D1	D1-2-1	chr8	104931069	104931069	T	C	Ces2e	Missense	NM_172759	c.T919C	p.F307L	0.433
D1	D1-2-1	chr8	33572140	33572140	C	T	Tex15	Missense	NM_031374	c.C1597T	p.L533F	0.356
D1	D1-2-1	chr9	43279886	43279886	G	A	D630033O11Rik	Missense	NM_001243261	c.G348A	p.M116I	0.491
D1	D1-2-1	chr9	3634462	3634462	G	A	Gucy1a2	Missense	NM_001033322	c.G505A	p.E169K	0.473
D1	D1-2-1	chr9	38403692	38403692	G	T	Olfr147	Nonsense	NM_146869	c.G817T	p.G273X	0.446
D1	D1-2-1	chr9	78313067	78313067	G	A	Omt2a	Missense	NM_001164523	c.C49T	p.L17F	0.485
D1	D1-2-1	chr9	48832304	48832304	G	A	Zbtb16	Missense	NM_001033324	c.C707T	p.P236L	0.464
D1	D1-2-1	chr10	89714952	89714952	G	A	Actr6	Missense	NM_025914	c.C979T	p.P327S	0.552
D1	D1-2-1	chr10	128953950	128953950	C	A	Itga7	Missense	NM_008398	c.C3059A	p.P1020Q	0.449
D1	D1-2-1	chr10	88548796	88548796	G	A	Mybpc1	Missense	NM_001252372	c.C1625T	p.P542L	0.515
D1	D1-2-1	chr11	85178450	85178450	G	A	1700125H20Rik	Missense	NM_028589	c.G623A	p.G208E	0.553
D1	D1-2-1	chr11	75432578	75432578	C	T	Serpinf2	Missense	NM_008878	c.G1301A	p.R434K	0.48
D1	D1-2-1	chr12	3367043	3367043	C	T	Kif3c	Missense	NM_008445	c.C1063T	p.L355F	0.583
D1	D1-2-1	chr12	69722113	69722113	A	T	L2hgdh	Missense	NM_145443	c.T224A	p.L75H	0.551
D1	D1-2-1	chr13	92752398	92752398	G	A	Thbs4	Missense	NM_011582	c.C2786T	p.S929F	0.488
D1	D1-2-1	chr14	28522675	28522675	C	T	Wnt5a	Missense	NM_001256224	c.C818T	p.S273F	0.484
D1	D1-2-1	chr15	20666089	20666089	C	T	Acot10	Missense	NM_022816	c.G565A	p.D189N	0.42
D1	D1-2-1	chr15	98594461	98594461	G	A	Adcy6	Missense	NM_007405	c.C2921T	p.S974L	0.506
D1	D1-2-1	chr17	36128961	36128961	C	T	Gm7030	Missense	NM_001177467	c.G280A	p.E94K	0.356
D1	D1-2-1	chr17	71504803	71504803	A	C	Ndc80	Missense	NM_023294	c.T1427G	p.L476R	0.525
D1	D1-2-1	chr18	38254491	38254491	C	T	0610009O20Rik	Missense	NM_024179	c.C682T	p.L228F	0.492
D1	D1-2-1	chr18	7129517	7129517	T	A	Armc4	Missense	NM_001081393	c.A2660T	p.N887I	0.5
D1	D1-2-1	chr19	36057177	36057177	C	A	Htr7	Missense	NM_008315	c.G77T	p.R26L	0.555
D1	D1-2-1	chrX	100573860	100573860	G	A	Awat1	Nonsense	NM_001081136	c.G215A	p.W72X	0.6
D1	D1-2-1	chrX	129972594	129972594	C	T	Diap2	Missense	NM_172493	c.C2038T	p.P680S	0.493
D1	D1-2-1	chrX	152209512	152209512	C	T	Iqsec2	Missense	NM_001114664	c.C2144T	p.S715F	0.545
D1	D1-2-1	chrX	66654019	66654019	G	A	Slitrk2	Missense	NM_001161431	c.G115A	p.E39K	0.45
D1	D1-2-2	chr1	43742308	43742308	G	A	1500015O10Rik	Missense	NM_024283	c.G349A	p.D117N	0.489
D1	D1-2-2	chr1	58233915	58233915	G	A	Aox4	Missense	NM_023631	c.G988A	p.E330K	0.714
D1	D1-2-2	chr1	160577443	160577443	T	A	Gpr52	Missense	NM_001146330	c.A311T	p.Y104F	0.5
D1	D1-2-2	chr2	83879660	83879660	A	G	Fam171b	Missense	NM_175514	c.A1675G	p.R559G	0.495
D1	D1-2-2	chr2	41449323	41449323	T	A	Lrp1b	Missense	NM_053011	c.A2507T	p.N836I	0.409
D1	D1-2-2	chr2	87753589	87753589	C	T	Olfr1141	Missense	NM_146637	c.G403A	p.D135N	0.408
D1	D1-2-2	chr3	87975556	87975556	A	G	Nes	Missense	NM_016701	c.A1121G	p.K374R	0.488
D1	D1-2-2	chr3	102941078	102941078	A	G	Nr1h5	Missense	NM_198658	c.T1310C	p.L437P	0.486
D1	D1-2-2	chr3	109827654	109827654	C	T	Ntng1	Missense	NM_030699	c.G1363A	p.G455R	0.321
D1	D1-2-2	chr3	129811729	129811729	G	A	Rrh	Missense	NM_009102	c.C602T	p.P201L	0.473
D1	D1-2-2	chr3	5412423	5412423	G	A	Zfhx4	Missense	NM_030708	c.G10022A	p.S3341N	0.455
D1	D1-2-2	chr4	101814920	101814920	C	T	Lepr	Missense	NM_146146	c.C3140T	p.S1047L	0.494
D1	D1-2-2	chr4	43101826	43101826	T	A	Unc13b	Nonsense	NM_021468	c.T420A	p.Y140X	0.613
D1	D1-2-2	chr5	101814467	101814467	C	T	Cds1	Missense	NM_173370	c.C947T	p.S316F	0.459
D1	D1-2-2	chr5	4757484	4757484	C	T	Fzd1	Missense	NM_021457	c.G97A	p.G33R	0.457
D1	D1-2-2	chr5	106845458	106845458	C	T	Hfm1	Missense	NM_177873	c.G3898A	p.D1300N	0.551
D1	D1-2-2	chr5	33668158	33668158	C	T	Tacc3	Missense	NM_001040435	c.C1231T	p.L411F	0.463
D1	D1-2-2	chr6	142449487	142449487	A	T	Gys2	Missense	NM_145572	c.T1251A	p.N417K	0.471
D1	D1-2-2	chr7	103451408	103451408	G	A	Olfr606	Missense	NM_147094	c.G70A	p.E24K	0.548
D1	D1-2-2	chr7	35584548	35584548	C	A	Rgs9bp	Missense	NM_145840	c.G673T	p.V225L	0.497
D1	D1-2-2	chr8	104931069	104931069	T	C	Ces2e	Missense	NM_172759	c.T919C	p.F307L	0.342
D1	D1-2-2	chr8	33572140	33572140	C	T	Tex15	Missense	NM_031374	c.C1597T	p.L533F	0.539
D1	D1-2-2	chr9	43279886	43279886	G	A	D630033O11Rik	Missense	NM_001243261	c.G348A	p.M116I	0.523
D1	D1-2-2	chr9	3634462	3634462	G	A	Gucy1a2	Missense	NM_001033322	c.G505A	p.E169K	0.462
D1	D1-2-2	chr9	38403692	38403692	G	T	Olfr147	Nonsense	NM_146869	c.G817T	p.G273X	0.463
D1	D1-2-2	chr9	78313067	78313067	G	A	Omt2a	Missense	NM_001164523	c.C49T	p.L17F	0.515
D1	D1-2-2	chr9	48832304	48832304	G	A	Zbtb16	Missense	NM_001033324	c.C707T	p.P236L	0.422
D1	D1-2-2	chr10	89714952	89714952	G	A	Actr6	Missense	NM_025914	c.C979T	p.P327S	0.433
D1	D1-2-2	chr10	128953950	128953950	C	A	Itga7	Missense	NM_008398	c.C3059A	p.P1020Q	0.483
D1	D1-2-2	chr10	88548796	88548796	G	A	Mybpc1	Missense	NM_001252372	c.C1625T	p.P542L	0.432
D1	D1-2-2	chr11	85178450	85178450	G	A	1700125H20Rik	Missense	NM_028589	c.G623A	p.G208E	0.4
D1	D1-2-2	chr11	75432578	75432578	C	T	Serpinf2	Missense	NM_008878	c.G1301A	p.R434K	0.473
D1	D1-2-2	chr12	3367043	3367043	C	T	Kif3c	Missense	NM_008445	c.C1063T	p.L355F	0.496
D1	D1-2-2	chr12	69722113	69722113	A	T	L2hgdh	Missense	NM_145443	c.T224A	p.L75H	0.568
D1	D1-2-2	chr13	92752398	92752398	G	A	Thbs4	Missense	NM_011582	c.C2786T	p.S929F	0.449
D1	D1-2-2	chr14	28522675	28522675	C	T	Wnt5a	Missense	NM_001256224	c.C818T	p.S273F	0.497
D1	D1-2-2	chr15	20666089	20666089	C	T	Acot10	Missense	NM_022816	c.G565A	p.D189N	0.429
D1	D1-2-2	chr15	98594461	98594461	G	A	Adcy6	Missense	NM_007405	c.C2921T	p.S974L	0.491
D1	D1-2-2	chr17	71504803	71504803	A	C	Ndc80	Missense	NM_023294	c.T1427G	p.L476R	0.61
D1	D1-2-2	chr18	38254491	38254491	C	T	0610009O20Rik	Missense	NM_024179	c.C682T	p.L228F	0.504
D1	D1-2-2	chr18	7129517	7129517	T	A	Armc4	Missense	NM_001081393	c.A2660T	p.N887I	0.429
D1	D1-2-2	chr19	36057177	36057177	C	A	Htr7	Missense	NM_008315	c.G77T	p.R26L	0.508
D1	D1-2-2	chrX	100573860	100573860	G	A	Awat1	Nonsense	NM_001081136	c.G215A	p.W72X	0.489
D1	D1-2-2	chrX	129972594	129972594	C	T	Diap2	Missense	NM_172493	c.C2038T	p.P680S	0.408
D1	D1-2-2	chrX	152209512	152209512	C	T	Iqsec2	Missense	NM_001114664	c.C2144T	p.S715F	0.471
D1	D1-2-2	chrX	66654019	66654019	G	A	Slitrk2	Missense	NM_001161431	c.G115A	p.E39K	0.521
D1	D1-2-3	chr1	43742308	43742308	G	A	1500015O10Rik	Missense	NM_024283	c.G349A	p.D117N	0.436
D1	D1-2-3	chr1	58233915	58233915	G	A	Aox4	Missense	NM_023631	c.G988A	p.E330K	0.55
D1	D1-2-3	chr1	160577443	160577443	T	A	Gpr52	Missense	NM_001146330	c.A311T	p.Y104F	0.479
D1	D1-2-3	chr2	83879660	83879660	A	G	Fam171b	Missense	NM_175514	c.A1675G	p.R559G	0.464
D1	D1-2-3	chr2	41449323	41449323	T	A	Lrp1b	Missense	NM_053011	c.A2507T	p.N836I	0.444
D1	D1-2-3	chr2	87753589	87753589	C	T	Olfr1141	Missense	NM_146637	c.G403A	p.D135N	0.448
D1	D1-2-3	chr3	87975556	87975556	A	G	Nes	Missense	NM_016701	c.A1121G	p.K374R	0.46
D1	D1-2-3	chr3	102941078	102941078	A	G	Nr1h5	Missense	NM_198658	c.T1310C	p.L437P	0.364
D1	D1-2-3	chr3	109827654	109827654	C	T	Ntng1	Missense	NM_030699	c.G1363A	p.G455R	0.522
D1	D1-2-3	chr3	129811729	129811729	G	A	Rrh	Missense	NM_009102	c.C602T	p.P201L	0.44
D1	D1-2-3	chr3	5412423	5412423	G	A	Zfhx4	Missense	NM_030708	c.G10022A	p.S3341N	0.552
D1	D1-2-3	chr4	101814920	101814920	C	T	Lepr	Missense	NM_146146	c.C3140T	p.S1047L	0.425
D1	D1-2-3	chr4	43101826	43101826	T	A	Unc13b	Nonsense	NM_021468	c.T420A	p.Y140X	0.563
D1	D1-2-3	chr5	101814467	101814467	C	T	Cds1	Missense	NM_173370	c.C947T	p.S316F	0.54
D1	D1-2-3	chr5	4757484	4757484	C	T	Fzd1	Missense	NM_021457	c.G97A	p.G33R	0.529
D1	D1-2-3	chr5	106845458	106845458	C	T	Hfm1	Missense	NM_177873	c.G3898A	p.D1300N	0.475
D1	D1-2-3	chr5	33668158	33668158	C	T	Tacc3	Missense	NM_001040435	c.C1231T	p.L411F	0.495
D1	D1-2-3	chr6	142449487	142449487	A	T	Gys2	Missense	NM_145572	c.T1251A	p.N417K	0.418
D1	D1-2-3	chr7	103451408	103451408	G	A	Olfr606	Missense	NM_147094	c.G70A	p.E24K	0.489
D1	D1-2-3	chr7	35584548	35584548	C	A	Rgs9bp	Missense	NM_145840	c.G673T	p.V225L	0.477
D1	D1-2-3	chr8	104931069	104931069	T	C	Ces2e	Missense	NM_172759	c.T919C	p.F307L	0.543
D1	D1-2-3	chr8	33572140	33572140	C	T	Tex15	Missense	NM_031374	c.C1597T	p.L533F	0.533
D1	D1-2-3	chr9	43279886	43279886	G	A	D630033O11Rik	Missense	NM_001243261	c.G348A	p.M116I	0.5
D1	D1-2-3	chr9	3634462	3634462	G	A	Gucy1a2	Missense	NM_001033322	c.G505A	p.E169K	0.459
D1	D1-2-3	chr9	38403692	38403692	G	T	Olfr147	Nonsense	NM_146869	c.G817T	p.G273X	0.475
D1	D1-2-3	chr9	78313067	78313067	G	A	Omt2a	Missense	NM_001164523	c.C49T	p.L17F	0.467
D1	D1-2-3	chr9	48832304	48832304	G	A	Zbtb16	Missense	NM_001033324	c.C707T	p.P236L	0.488
D1	D1-2-3	chr10	89714952	89714952	G	A	Actr6	Missense	NM_025914	c.C979T	p.P327S	0.579
D1	D1-2-3	chr10	128953950	128953950	C	A	Itga7	Missense	NM_008398	c.C3059A	p.P1020Q	0.426
D1	D1-2-3	chr10	88548796	88548796	G	A	Mybpc1	Missense	NM_001252372	c.C1625T	p.P542L	0.437
D1	D1-2-3	chr11	85178450	85178450	G	A	1700125H20Rik	Missense	NM_028589	c.G623A	p.G208E	0.526
D1	D1-2-3	chr11	75432578	75432578	C	T	Serpinf2	Missense	NM_008878	c.G1301A	p.R434K	0.56
D1	D1-2-3	chr12	3367043	3367043	C	T	Kif3c	Missense	NM_008445	c.C1063T	p.L355F	0.481
D1	D1-2-3	chr12	69722113	69722113	A	T	L2hgdh	Missense	NM_145443	c.T224A	p.L75H	0.306
D1	D1-2-3	chr13	92752398	92752398	G	A	Thbs4	Missense	NM_011582	c.C2786T	p.S929F	0.418
D1	D1-2-3	chr14	28522675	28522675	C	T	Wnt5a	Missense	NM_001256224	c.C818T	p.S273F	0.46
D1	D1-2-3	chr15	20666089	20666089	C	T	Acot10	Missense	NM_022816	c.G565A	p.D189N	0.417
D1	D1-2-3	chr15	98594461	98594461	G	A	Adcy6	Missense	NM_007405	c.C2921T	p.S974L	0.51
D1	D1-2-3	chr17	36128961	36128961	C	T	Gm7030	Missense	NM_001177467	c.G280A	p.E94K	0.4
D1	D1-2-3	chr17	71504803	71504803	A	C	Ndc80	Missense	NM_023294	c.T1427G	p.L476R	0.425
D1	D1-2-3	chr18	38254491	38254491	C	T	0610009O20Rik	Missense	NM_024179	c.C682T	p.L228F	0.423
D1	D1-2-3	chr18	7129517	7129517	T	A	Armc4	Missense	NM_001081393	c.A2660T	p.N887I	0.56
D1	D1-2-3	chr19	36057177	36057177	C	A	Htr7	Missense	NM_008315	c.G77T	p.R26L	0.521
D1	D1-2-3	chrX	100573860	100573860	G	A	Awat1	Nonsense	NM_001081136	c.G215A	p.W72X	0.451
D1	D1-2-3	chrX	129972594	129972594	C	T	Diap2	Missense	NM_172493	c.C2038T	p.P680S	0.479
D1	D1-2-3	chrX	152209512	152209512	C	T	Iqsec2	Missense	NM_001114664	c.C2144T	p.S715F	0.494
D1	D1-2-3	chrX	66654019	66654019	G	A	Slitrk2	Missense	NM_001161431	c.G115A	p.E39K	0.46
D2	D2-1-1	chr1	135968785	135968785	C	T	Igfn1	Missense	NM_177642	c.G4042A	p.G1348S	0.25

D2	D2-1-1	chr1	133329529	133329529	C	T	Kiss1	Missense	NM_178260	c.C265T	p.P89S	0.331
D2	D2-1-1	chr1	157175868	157175868	T	G	Rasal2	Missense	NM_177644	c.A1274C	p.K425T	0.143
D2	D2-1-1	chr1	131977494	131977494	G	A	Slc45a3	Missense	NM_001177628	c.G254A	p.R85Q	0.136
D2	D2-1-1	chr1	75532637	75532637	C	A	Stk11ip	Nonsense	NM_027886	c.C2298A	p.C766X	0.132
D2	D2-1-1	chr2	154368820	154368820	G	A	Cdk5rap1	Missense	NM_025876	c.C397T	p.L133F	0.114
D2	D2-1-1	chr2	153622686	153622686	G	A	Commd7	Missense	NM_133850	c.C301T	p.L101F	0.104
D2	D2-1-1	chr2	165292083	165292083	G	A	Elmo2	Missense	NM_207705	c.C1984T	p.L662F	0.298
D2	D2-1-1	chr2	167983186	167983186	C	T	Fam65c	Missense	NM_001080708	c.G2402A	p.G801E	0.137
D2	D2-1-1	chr2	132915191	132915191	C	T	Fermt1	Missense	NM_198029	c.G1718A	p.R573K	0.118
D2	D2-1-1	chr2	71841706	71841706	C	T	Itga6	Missense	NM_008397	c.C2465T	p.S822F	0.342
D2	D2-1-1	chr2	155622647	155622647	G	A	Myh7b	Missense	NM_001085378	c.G1903A	p.E635K	0.174
D2	D2-1-1	chr2	86988709	86988709	G	A	Olfr1101	Missense	NM_146591	c.C466T	p.H156Y	0.092
D2	D2-1-1	chr2	153865380	153865380	C	T	Sun5	Missense	NM_029599	c.G377A	p.G126E	0.242
D2	D2-1-1	chr2	26916799	26916799	C	T	Surf2	Missense	NM_013678	c.C173T	p.S58F	0.124
D2	D2-1-1	chr2	21213075	21213075	G	A	Thnsl1	Missense	NM_177588	c.G1639A	p.G547R	0.361
D2	D2-1-1	chr3	89033297	89033297	C	T	Ash1l	Missense	NM_138679	c.C6127T	p.P2043S	0.324
D2	D2-1-1	chr3	84474415	84474415	G	A	Fhdc1	Missense	NM_001033301	c.C226T	p.P76S	0.131
D2	D2-1-1	chr3	107105483	107105483	A	G	Kcna2	Missense	NM_008417	c.A1379G	p.E460G	0.335
D2	D2-1-1	chr3	36671189	36671189	C	T	Trpc3	Missense	NM_019510	c.G637A	p.D213N	0.326
D2	D2-1-1	chr3	152368254	152368254	C	T	Usp33	Missense	NM_133247	c.C736T	p.R246C	0.222
D2	D2-1-1	chr4	41499553	41499553	G	T	AI464131	Missense	NM_001085515	c.C76A	p.L26I	0.403
D2	D2-1-1	chr4	141308592	141308592	C	T	Epha2	Missense	NM_010139	c.C338T	p.S113L	0.147
D2	D2-1-1	chr4	130718248	130718248	C	T	Pum1	Missense	NM_030722	c.C527T	p.A176V	0.079
D2	D2-1-1	chr4	129080462	129080462	C	T	Rnf19b	Missense	NM_029219	c.C1514T	p.P505L	0.248
D2	D2-1-1	chr4	129294260	129294260	G	A	Sync	Missense	NM_023485	c.G1084A	p.E362K	0.303
D2	D2-1-1	chr4	120931266	120931266	G	A	Zfp69	Missense	NM_001005788	c.C851T	p.P284L	0.25
D2	D2-1-1	chr5	92941071	92941071	C	T	Shroom3	Missense	NM_015756	c.C1679T	p.S560L	0.274
D2	D2-1-1	chr5	137463449	137463449	G	C	Zan	Missense	NM_011741	c.C3467G	p.S1156C	0.141
D2	D2-1-1	chr6	146952391	146952391	C	T	1700034J05Rik	Nonsense	NM_028509	c.G767A	p.W256X	0.125
D2	D2-1-1	chr6	125463701	125463701	T	A	Cd9	Missense	NM_007657	c.A326T	p.Y109F	0.114
D2	D2-1-1	chr6	136043944	136043944	G	A	Grin2b	Missense	NM_008171	c.C358T	p.L120F	0.197
D2	D2-1-1	chr6	141817717	141817717	C	T	Slco1a4	Splice site	NM_030687	c.1271+1G>A	NA	0.131
D2	D2-1-1	chr6	108144689	108144689	G	A	Sumf1	Missense	NM_145937	c.C850T	p.P284S	0.188
D2	D2-1-1	chr7	126582813	126582813	G	A	Cln3	Missense	NM_009907	c.C76T	p.P26S	0.09
D2	D2-1-1	chr7	119916240	119916240	C	A	Lyrm1	Missense	NM_029610	c.C352A	p.H118N	0.209
D2	D2-1-1	chr7	141866900	141866900	G	T	Muc5b	Missense	NM_028801	c.G12503T	p.C4168F	0.285
D2	D2-1-1	chr7	45054552	45054552	C	T	Prrg2	Missense	NM_022999	c.G545A	p.G182E	0.14
D2	D2-1-1	chr8	95242266	95242266	G	A	Cngb1	Missense	NM_001195413	c.C3613T	p.L1205F	0.288
D2	D2-1-1	chr8	27199371	27199371	G	A	Got1l1	Missense	NM_029674	c.C659T	p.S220F	0.203
D2	D2-1-1	chr9	49419478	49419478	C	T	Ankk1	Missense	NM_172922	c.G762A	p.M254I	0.151
D2	D2-1-1	chr9	46242403	46242403	G	A	Apoa4	Missense	NM_007468	c.G301A	p.E101K	0.275
D2	D2-1-1	chr9	45854694	45854694	G	A	Bace1	Missense	NM_011792	c.G418A	p.E140K	0.388
D2	D2-1-1	chr9	108190201	108190201	C	T	Bsn	Missense	NM_007567	c.G58A	p.G20S	0.168
D2	D2-1-1	chr9	108829695	108829695	C	T	Celsr3	Missense	NM_080437	c.C3376T	p.R1126C	0.152
D2	D2-1-1	chr9	58199740	58199740	G	A	Islr2	Missense	NM_001161541	c.C236T	p.S79L	0.335
D2	D2-1-1	chr9	66970932	66970932	G	A	Lactb	Missense	NM_030717	c.C614T	p.S205F	0.258
D2	D2-1-1	chr9	7447297	7447297	G	T	Mmp3	Missense	NM_010809	c.G386T	p.R129I	0.325
D2	D2-1-1	chr9	38855552	38855552	G	A	Olfr26	Missense	NM_146783	c.G489A	p.M163I	0.25
D2	D2-1-1	chr9	110861292	110861292	C	A	Prss50	Missense	NM_146227	c.C502A	p.L168I	0.146
D2	D2-1-1	chr9	107752115	107752115	G	A	Rbm5	Missense	NM_148930	c.C1037T	p.T346I	0.196
D2	D2-1-1	chr9	44683054	44683054	G	A	Treh	Missense	NM_021481	c.G614A	p.G205E	0.246
D2	D2-1-1	chr10	80341782	80341782	G	A	Adamtsl5	Missense	NM_001113548	c.C1045T	p.P349S	0.147
D2	D2-1-1	chr10	127193169	127193169	G	A	Dtx3	Missense	NM_030714	c.C190T	p.P64S	0.186
D2	D2-1-1	chr10	127370372	127370372	G	A	Inhbc	Missense	NM_010565	c.C25T	p.L9F	0.139
D2	D2-1-1	chr10	7801956	7801956	G	T	Ppil4	Missense	NM_026141	c.G790T	p.G264W	0.3
D2	D2-1-1	chr10	89776005	89776005	C	T	Uhrf1bp1l	Missense	NM_029166	c.C313T	p.P105S	0.378
D2	D2-1-1	chr11	5130674	5130674	G	A	Emid1	Missense	NM_080595	c.C775T	p.P259S	0.12
D2	D2-1-1	chr11	97334964	97334964	G	A	Gpr179	Nonsense	NM_001081220	c.C6364T	p.Q2122X	0.372
D2	D2-1-1	chr11	100145811	100145811	C	T	Krt19	Missense	NM_008471	c.G49A	p.G17S	0.528
D2	D2-1-1	chr11	86286608	86286608	G	A	Med13	Missense	NM_001080931	c.C4661T	p.S1554L	0.202
D2	D2-1-1	chr11	78015583	78015583	A	T	Phf12	Missense	NM_174852	c.A922T	p.M308L	0.136
D2	D2-1-1	chr11	20314058	20314058	C	T	Slc1a4	Missense	NM_018861	c.G658A	p.G220R	0.109
D2	D2-1-1	chr12	80759527	80759527	C	T	Ccdc177	Splice site	NM_001008423	c.-28-1G>A	NA	0.282
D2	D2-1-1	chr12	104101802	104101802	C	T	Serpina5	Missense	NM_172953	c.C121T	p.P41S	0.155
D2	D2-1-1	chr12	75899152	75899152	A	T	Syne2	Missense	NM_001005510	c.A997T	p.S333C	0.344
D2	D2-1-1	chr13	61353808	61353809	AT	TA	Cts7	Missense	NM_019539	c.AT876_877TA	p.KY292_293	0.152
D2	D2-1-1	chr13	112626334	112626334	G	T	Ddx4	Missense	NM_001145885	c.C487A	p.L163I	0.337
D2	D2-1-1	chr13	21468185	21468185	G	T	Nkapl	Missense	NM_025719	c.C257A	p.S86Y	0.182
D2	D2-1-1	chr14	35569565	35569565	C	T	Grid1	Nonsense	NM_008166	c.C2428T	p.R810X	0.345
D2	D2-1-1	chr14	50892697	50892697	-	G	Klhl33	Frameshift insertion	NM_001166651	c.559_560insC	p.D187fs	0.123
D2	D2-1-1	chr14	50361709	50361709	G	A	Tlr11	Missense	NM_205819	c.G1151A	p.G384E	0.204
D2	D2-1-1	chr15	39842076	39842076	G	A	Dpys	Missense	NM_022722	c.C640T	p.P214S	0.434
D2	D2-1-1	chr15	85821661	85821661	G	T	Pkdrej	Missense	NM_011105	c.C73A	p.P25T	0.159
D2	D2-1-1	chr16	8600949	8600950	CC	TT	Abat	Missense	NM_172961	c.CC406_407TT	p.P136L	0.107
D2	D2-1-1	chr16	90951887	90951887	G	A	Synj1	Missense	NM_001164483	c.C3280T	p.P1094S	0.326
D2	D2-1-1	chr17	35637937	35637937	C	T	Dpcr1	Missense	NM_001033366	c.G769A	p.E257K	0.216
D2	D2-1-1	chr17	56394823	56394823	C	T	Kdm4b	Missense	NM_172132	c.C1831T	p.P611S	0.252
D2	D2-1-1	chr17	14955115	14955115	T	A	Phf10	Missense	NM_024250	c.A458T	p.Y153F	0.392
D2	D2-1-1	chr18	61220938	61220938	C	T	Pde6a	Missense	NM_146086	c.C334T	p.L112F	0.333
D2	D2-1-1	chr19	47648228	47648228	C	T	Col17a1	Splice site	NM_007732	c.3977-1G>A	NA	0.318
D2	D2-1-1	chr19	44532612	44532612	C	T	Sec31b	Nonsense	NM_001033343	c.G864A	p.W288X	0.357
D2	D2-1-1	chrX	112243346	112243346	G	T	4933403O08Rik	Nonsense	NM_001177389	c.G808T	p.G270X	0.368
D2	D2-1-1	chrX	20517159	20517159	C	T	Phf16	Missense	NM_199317	c.C1735T	p.P579S	0.305
D2	D2-1-1	chrX	150398538	150398538	G	A	Tmem29	Missense	NM_001164683	c.C256T	p.P86S	0.084
E1	E1-1-1	chr15	35073217	35073217	G	A	Stk3	Missense	NM_019635	c.C356T	p.T119I	0.385
E1	E1-1-1	chrX	96160175	96160175	C	T	Msn	Missense	NM_010833	c.C883T	p.R295C	0.331
E1	E1-1-1	chrX	162784385	162784385	T	G	Txlng	Missense	NM_178935	c.A1089C	p.E363D	0.386
E1	E1-2-1	chr14	73322031	73322031	C	T	Rb1	Missense	NM_009029	c.G232A	p.V78M	0.188
E1	E1-2-1	chrX	96160175	96160175	C	T	Msn	Missense	NM_010833	c.C883T	p.R295C	0.49
E1	E1-2-1	chrX	162784385	162784385	T	G	Txlng	Missense	NM_178935	c.A1089C	p.E363D	0.414
E1	E1-2-2	chr15	35073217	35073217	G	A	Stk3	Missense	NM_019635	c.C356T	p.T119I	0.475
E1	E1-2-2	chrX	96160175	96160175	C	T	Msn	Missense	NM_010833	c.C883T	p.R295C	0.61
E1	E1-2-2	chrX	162784385	162784385	T	G	Txlng	Missense	NM_178935	c.A1089C	p.E363D	0.515

Abbreviations: NA, not applicable.
